# Chemoenzymatic Synthesis and Biological Recognition of a Sulfonate Isostere of 6‑Sulfo-sialyl Lewis^x^

**DOI:** 10.1021/jacsau.5c00443

**Published:** 2025-06-15

**Authors:** Yunfei Wu, Julia Weber, Anne L. M. Kimpel, Luca Unione, Elif Uslu, Robert P. de Vries, Geert-Jan Boons

**Affiliations:** 1 Chemical Biology and Drug Discovery, Utrecht Institute for Pharmaceutical Sciences, 8125 Utrecht University, Universiteitsweg 99, Utrecht 3584 CG, The Netherlands; 2 Center for Cooperative Research in Biosciences (CIC bioGUNE), Basque Research and Technology Alliance (BRTA), Derio 48160, Spain; 3 Ikerbasque, Basque Foundation for Science, Euskadi Plaza 5, Bizkaia, Bilbao 48009, Spain; 4 Complex Carbohydrate Research Center, 1355 University of Georgia, 315 Riverbend Road, Athens, Georgia 30602, United States; 5 Bijvoet Center for Biomolecular Research, 8125 Utrecht University, Utrecht 3584 CG, The Netherlands; 6 Department of Chemistry, 1355 University of Georgia, Athens, Georgia 30602, United States

**Keywords:** chemoenzymatic synthesis, glycomimetic, glycosyltransferases, glycan microarray, influenza A, Siglec

## Abstract

Sulfation of *N*-acetylglucosamine (GlcNAc) moieties of glycans is a common modification that has been implicated in many biological and disease processes. Glycans having sulfate replaced by a stable analogue may find use as glycomimetic drugs. Here, we describe the synthesis of analogues of UDP-GlcNAc in which C-6 hydroxyl is replaced by a thiol or disulfide-protected thiol. It was found that UDP-GlcNAc-6-deoxy-6-SH is a donor substrate for GCNT1 and UDP-GlcNAc-6-deoxy-6-S-SMe for B3GnT2, allowing the preparation of glycopeptides and oligo-LacNAc derivatives having a GlcNAc-6-deoxy-6-S-*R* moiety, respectively. The disulfide can be reduced to a thiol, which can easily be oxidized to the corresponding sulfonates. Furthermore, oligosaccharides having a sulfonate or disulfide at C-6 are appropriate substrates for glycosyltransferases, providing access to a panel of glycomimetics. The sulfonates and several reference glycans and glycopeptides were printed as a glycan microarray that was used to examine binding selectivities of several lectins, Siglecs, and hemagglutinins of influenza A viruses. It was found that sulfonates can either be tolerated, enhance binding as in the case of Siglec-4, or abolish recognition as for Siglec-9. Molecular modeling studies of the complex of Siglec-4 with sulfated and sulfonated sialyl LacNAc indicate that plasticity of the binding site of the protein and a great charge on oxygens of a sulfonate are responsible for the higher binding affinity. Introduction of a 6-sulfonate gives better step economy than conventional enzymatic sulfation, is simpler to operate, provides compounds resistant to hydrolysis by sulfatases, and can modulate binding selectivities.

## Graphical abstract

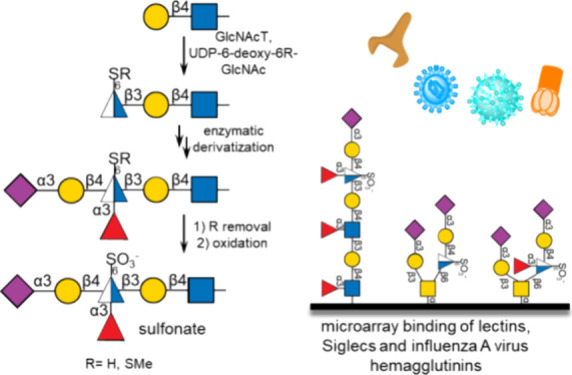

## Introduction

Sulfation is a common modification of glycans that is found in many species ranging from bacteria, plants, to higher animals.^1–3^ Sulfates impart negative charge to glycans, which is associated with the unique structural features and determines many biological functions.^4^ Sulfation of GlcNAc is prevalent and an important determinant of many biological processes in eukaryotic cells.^4^ For example, 6-*O*-sulfation of GlcNAc of sialyl Lewis^x^ [6-sulfo-SLe^x^; Neu5Acα­(2,3)­Galβ­(1,4)-[Fucα­(1,3)]­GlcNAc­(6-SO_3_^–^)­β-OR; Figure 1a] is a ligand for l-selectin that is important for lymphocyte extravasation and inflammatory response.^5–7^ Furthermore, distinct sulfation patterns of sialylated β(1→3)-linked *N*-acetyllactosamine [Galβ­(1,4)­GlcNAc] can serve as ligands for human sialic acid binding immunoglobulin-like lectins (Siglecs), such as Siglec-8 and Siglec-9.^8^ In addition, GlcNAc-6-*O*-sulfates are constituents of glycosaminoglycans (GAG), including keratan sulfate (KS; Figure 1a) and heparan sulfate, which ubiquitously decorate mammalian cells where they regulate extracellular cell signaling, growth factor signaling, innate immunity, inflammation, and homeostasis.^9–11^

**Figure 1:**
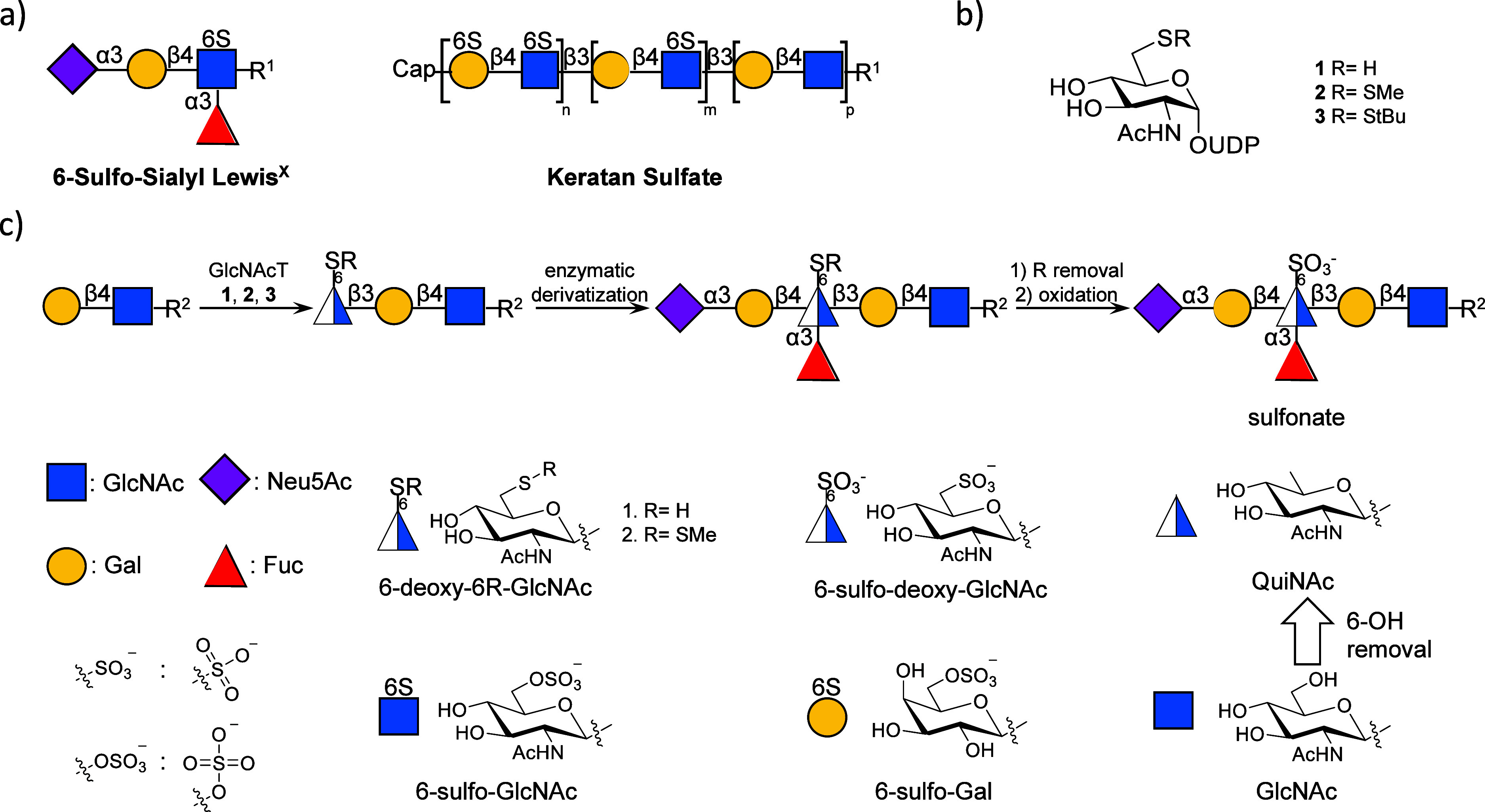
UDP-6-deoxy-6SR-GlcNAc used to mimic 6-*O*-sulfated GlcNAc oligosaccharides. (a) Some 6-*O*-sulfate GlcNAc glycans, R^1^ = N- or O-glycan. (b) UDP-GlcNAc-6-deoxy-6-thiol analogues. (c) Synthetic route of 6-deoxy-6-sulfonate-GlcNAc oligosaccharide mimics. R^2^ = linear- or O-glycan.

Sulfation of oligosaccharides occurs in the lumen of the Golgi apparatus where a family of membrane-bound sulfotransferases catalyzes the transfer of sulfate from 3′-phosphoadenosine-5′-phosphosulfate (PAPS) to glycan substrates.^1,2^ There are multiple types of carbohydrate sulfotransferases, and some of these can be expressed as a number of isoforms that have different substrate preferences.^12^ The biosynthesis of sulfated glycans is highly orchestrated; for example, the biosynthesis of 6-sulfo-sialyl Lewis^x^ is initiated by 6-*O*-sulfation of β-GlcNAc followed by β-galactosylation, sialylation, and finally fucosylation.^13–15^

To decipher the glycan “sulfation code”, which is proposed to mediate carbohydrate–protein interactions, panels of sulfated glycans have been prepared through chemical and chemoenzymatic approaches to establish structure–activity relationships (SAR).^10,16–18^ For example, we recently reported a biomimetic approach to prepare a panel of keratan sulfate (KS) oligosaccharides in which inherent enzyme selectivities were exploited to give poly-LacNAc derivatives having different patterns of fucosylation, sialylation, and sulfation.^19^

The preparation of bioisosteres is a common strategy to improve pharmaceutical products.^20,21^ Sulfonated polymers have been reported as heparin mimetics,^22–24^ and in these approaches, the chemically introduced sulfonates mimic negatively charged functional groups of heparin while minimizing heterogeneity and desulfation *in vivo*. Also, hydrolytically labile tyrosine sulfates have been replaced by stable sulfonate analogues for the development of glycosulfopeptide mimics of the *N*-terminus of PSGL-1 as a therapeutic.^25^

We were compelled to prepare oligosaccharides having GlcNAc-6-sulfate replaced by a sulfonate (Figure 1c) to give the corresponding isosteres. The synthetic approach exploits the use of analogues of UDP-GlcNAc in which C-6 hydroxyl is replaced by a thiol (**1**) or disulfide-protected thiol (**2**,**3**). It was found that derivative **1** is a donor substrate for GCNT1 and compound **2** for B3GnT2, allowing the preparation of glycopeptides and oligo-LacNAc derivatives having a terminal GlcNAc-6-deoxy-6-*S*-*R* moiety, respectively. Thiol-containing compounds could easily be oxidized to the corresponding sulfonate, which could be further extended by galactosyltransferases and other subsequently acting glycosyltransferases to give complex glycomimetics. Compounds, having a GlcNAc-6-deoxy-6-*S*-SMe moiety, are also appropriate substrates for glycosyltransferases and could at a late stage of synthesis be converted into a sulfonate by reduction of the disulfide followed by oxidation of the resulting thiol.

The sulfonates and several reference glycans and glycopeptides were printed as a glycan microarray, which was examined for binding of several lectins, Siglecs, and hemagglutinins of influenza A viruses. It was found that sulfonates can either be tolerated, enhance binding (Siglec-4), or abolish recognition (Siglec-9). *In silico* studies of Siglec-4 in complex with sulfated and sulfonated sialyl LacNAc indicate that the plasticity of the binding site of the protein and a great charge on oxygens of a sulfonate are responsible for higher binding affinity. Introduction of a 6-sulfonate gives better step economy than conventional enzymatic sulfation and is simpler to operate.^26^ Furthermore, 6-sulfonate-GlcNAc is not a substrate for sulfatases, which enhances biological stability.

## Results and Discussion

### Synthesis of UDP-GlcNAc-6-deoxy-6-thiol Analogues

It is well known that glycosyltransferases tolerate sugar nucleotides having particular chemical modifications in the sugar component.^27^ This feature has, for example, been exploited for the introduction of bio-orthogonal groups in glycoconjugates such as azido and alkyne moieties.^28^ Modified sugar nucleotides have also been used in chemoenzymatic synthesis of complex glycans; for example, we introduced a stop-and-go strategy in which a modified sugar nucleotide is employed that can be transferred by a glycosyltransferase to give a product in which the newly introduced monosaccharide temporarily disables further enzymatic modification.^29–32^

The preparation of UDP-GlcNAc-6-deoxy-6-SH (**1**) has been reported by a chemoenzymatic strategy for inhibition of the glycosyltransferase complex PgaCD in *Escherichia coli*.^33,34^ However, the preparation of this compound required a laborious procedure resulting in only milligram quantities. Furthermore, thiols have limited stability and can undergo disulfide formation. Therefore, we aimed to develop a scalable chemical procedure for UDP-GlcNAc analogues **2** and **3**, having the thiol at C-6 of GlcNAc protect as a methanethiol or *tert*-butyl group, respectively (Scheme 1). Thus, a one-pot tosylation of the 6-hydroxyl of *N*-acetylglucosamine **4** with tosyl chloride (TsCl) in pyridine followed by acetylation of the remaining hydroxyls by the addition of acetic anhydride gave, after purification by silica gel column chromatography, compound **5** in a yield of 88%. The anomeric acetate of compound **5** was selectively removed by hydrazine acetate and the crude product **6** was immediately phosphitylated with dibenzyl *N*,*N*-diisopropyl phosphoramidite and then oxidated with mCPBA to give anomeric phosphate **7**. Hydrogenolysis of the latter compound over Pd/C in methanol to remove the benzyl esters of the phosphate gave the resulting product, which was treated with potassium thioacetate to displace the tosylate to give compound **8** having a thioacetyl ester at C-6. Treatment of **8** with sodium methoxide in methanol resulted in the removal of the acetyl and thioacetyl esters, and the C-6 thiol of the resulting compound was protected as *tert*-butyl-disulfane by reaction with *tert*-butylmethanethiosulfonate to give compound **9**. Next, monophosphate **9** was treated with UMP-morpholidate in the presence of tetrazole in pyridine to generate **3** on a scale of several hundreds of milligrams. Some starting material (**9**) was observed, which is attributed to the slow rate of the reaction, which could easily be removed by flash column chromatography over silica gel. Finally, compound **3** was easily converted into thiol **1** by reduction with tris­(2-carboxyethyl)­phosphine (TCEP), which could be further reacted with *S*-methylmethanethiosulfonate to give derivative **2** in good yield.

**Scheme 1:**
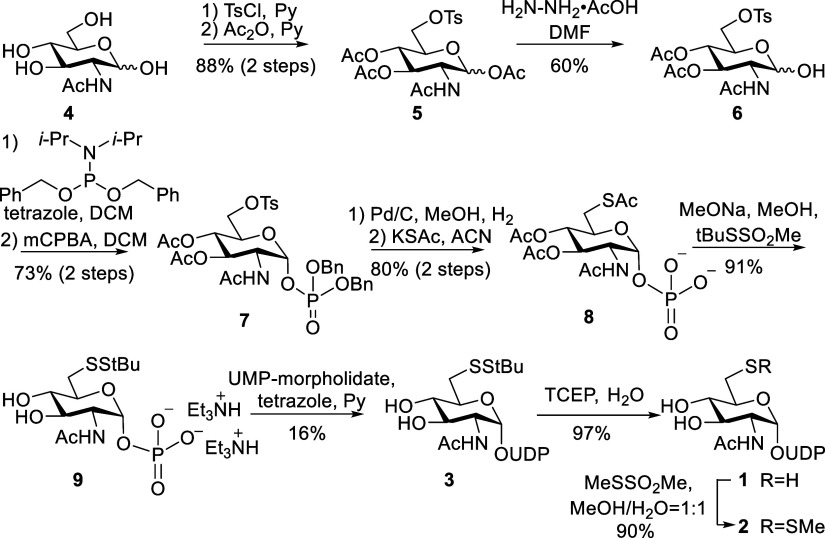
Chemical Synthesis of UDP-6-deoxy-6SR-GlcNAc Analogues **1**, **2**, and **3**.

### Enzymatic Transfer of UDP-GlcNAc-6-deoxy-6-S-R Analogues

To examine the influence of 6-thio derivatives of UDP-GlcNAc on enzymatic transformations, unnatural sugar nucleotides **1**, **2**, and **3** were examined as donor substrates for three *N*-acetylglucosaminyltransferases (Hpβ3GlcNAcT, GCNT1, and B3GnT2) using appropriate acceptors (Scheme 2). Unfortunately, compound **3** could not be transferred by any of the enzymes, which is attributed to the large size of the *tert*-Bu-S group at the C-6 position. None of the sugar nucleotides was transferred by the bacterial glycosyltransferase from *Helicobacter pylori* (Hpβ3GlcNAcT) either. However, compound **1** was tolerated to install a modified GlcNAc moiety at the upper branch of glycopeptide acceptor **10** by human β-1,6-*N*-acetylglucosaminyltransferase (GCNT1). Meanwhile, mammalian β-1,3-*N*-acetylglucosaminyltransferase (B3GnT2) could readily transfer **2** to LacNAc acceptor **17** to form pentasaccharide **18b**. It implies that GCNT1 and B3GnT2 accept small unnatural substitution at the C-6 position of the GlcNAc enabling transfer of modified sugar nucleotides.

**Scheme 2:**
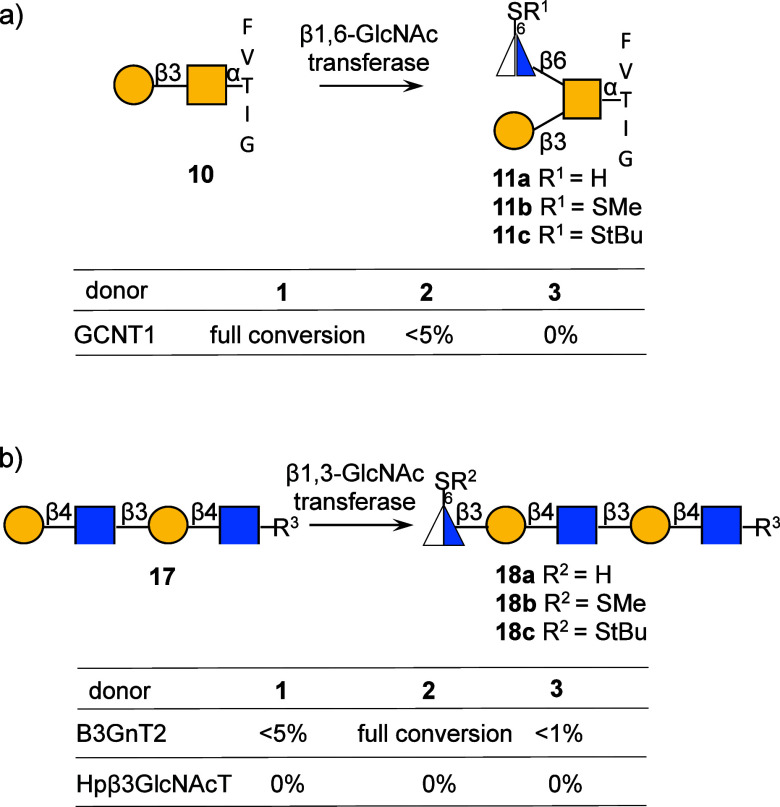
Enzymatic Transfer of UDP-6-Deoxy-6SR-GlcNAc Analogues **1**, **2**, and 3^a^

a R^3^= O­(CH_2_)_5_NHCbz. (a) Enzymatic transfer of UDP-6-deoxy-6SR-GlcNAc analogues to the glycopeptide acceptor. (b) Enzymatic transfer of UDP-6-deoxy-6SR-GlcNAc analogues to the linear glycan acceptor. (c) Conversion measured by LC-MS. (d) The test reactions were run in the 10 mM substrate solution with 15 mM (1.5 equiv) sugar nucleotide under 37 °C; the reaction was checked after 16 h.

### Enzymatic Synthesis of *O*-Linked Glycopeptides Having a Sulfonate

The side chain of serine and threonine residues of glycoproteins and mucins can be glycosylated with α-*N*-acetyl galactosamine (GalNAc).^35,36^ Core 2 type GalNAc glycans share a common trisaccharide (GlcNAcβ1,6­(Galβ1,3)­GalNAc) that can be further galactosylated, sialylated, fucosylated, and sulfated to generate a diverse set of glycan structures.^37^ GCNT1 is a core 2-branching glycosyltransferase that catalyzes the transfer of GlcNAc from UDP-GlcNAc to Galβ1–3GalNAc of *O*-linked glycoproteins resulting in the conversion of core 1 (Galβ1–3GalNAc) into core 2 (Galβ1–3­(GlcNAcβ1–6)­GalNAc).^38^ We found that GCNT1 is compatible with the unnatural sugar nucleotide **1**, having free thiol at C-6 of GlcNAc, and therefore set out to prepare core 2 type sulfonates **13**–**16** to demonstrate that GlcNAc-6-sulfonate can act as an acceptor for subsequent glycosyltransferase-catalyzed modifications (Scheme 3).

**Scheme 3:**
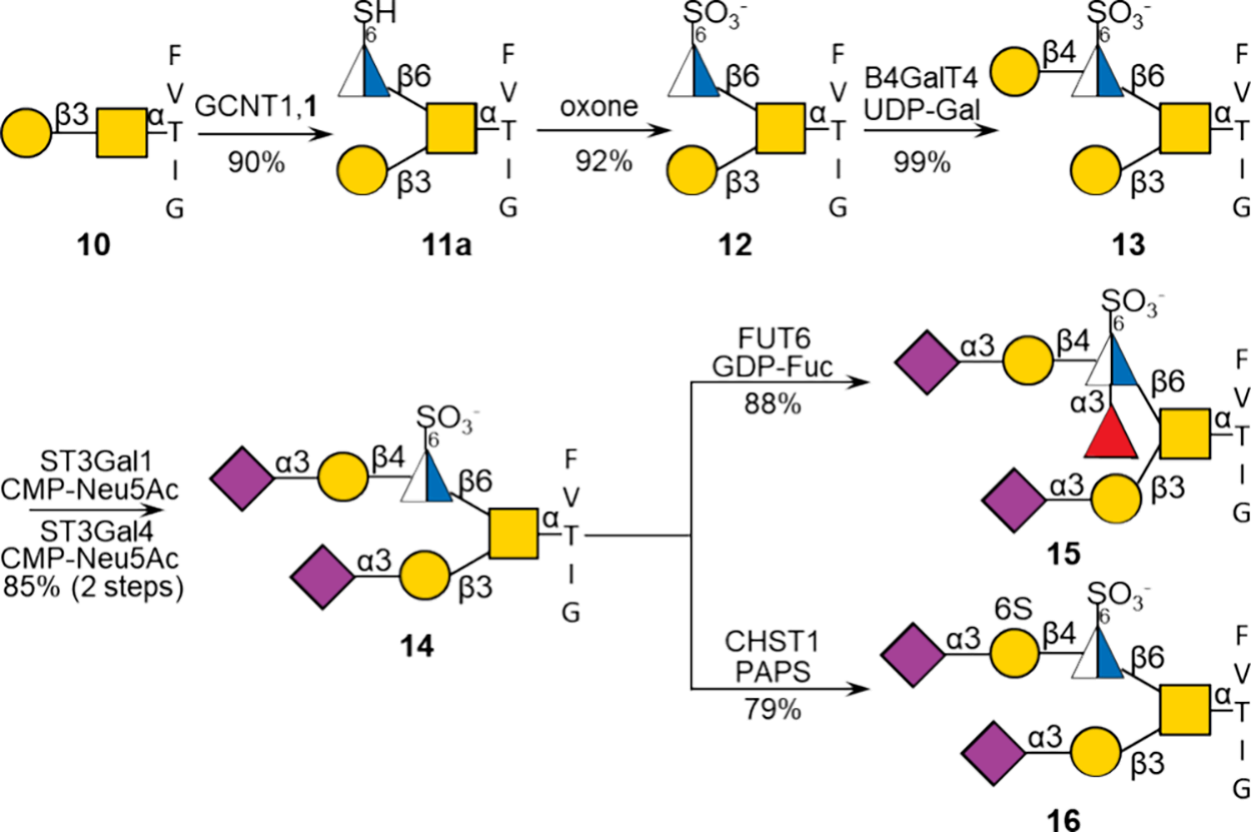
Enzymatic Synthesis of *O*-Glycan Sulfonate Mimics

Treatment of core 1 glycopeptide **10** with modified UDP-GlcNAc derivative **1** in the presence of GCNT1 gave, after purification by P2 size-exclusion column chromatography followed by preparative HPLC over a SeQuant ZIC-HILIC column, core 2 type glycopeptide **11a** having a GlcNAc-6-deoxy-6SH residue via a β1,6-linkage. The thiol of the latter derivative could readily be oxidized to a sulfonate using oxone to provide **12**. Compound **12** was purified by size-exclusion column chromatography over Bio-Gel P2 using 50 mM NH_4_HCO_3_ as an eluent.

It is known that the galactosyl transferase B4GalT4 can attach a β1,4-linked galactoside to a 6-sulfo-GlcNAc residue.^39^ Therefore, we treated **12** with UDP-Gal and B4GalT4, which resulted in the facile formation of **13**, and thus this enzyme tolerates an unnatural sulfonate at C-6 of GlcNAc as an acceptor. Subsequent treatment of **13** with ST3Gal1 and ST3Gal4 in the presence of CMP-Neu5Ac resulted in sialylation of core 1- and 2-arm providing disialoside **14**. Compound **14** was subjected to FUT6 in the presence of GDP-fucose (GDP-Fuc) resulting in 6-sulfo sialyl Lewis^x^ glycopeptide mimetic **15**. It has been reported that CHST1 can modify internal galactosides of oligo-LacNAc moieties and that the presence of a neighboring GlcNAc6S enhances this transformation.^40^ Compound **14** was incubated with CHST1 in the presence of PAPS resulting in the facile formation of compound **16** as a single product. Thus, the sulfonate of **14** also appears to facilitate sulfation of the neighboring Gal moiety. Detailed NMR analysis confirmed the structural integrity of the compounds, particularly the conversion of the C-6 position from the S-substitution in 6-deoxy-GlcNAc. For example, 1D ^1^H NMR and 2D ^1^H–^13^C HSQC spectra of compounds **11a** and **12** made it possible to assign all of the proton and carbon signals. The C-6 carbon of the thiol of the 6-deoxy-GlcNAc moiety in compound **11a** had a substantially upfield shift (δ 40.5), and the corresponding protons also exhibited a special chemical shift (H6a 3.33 and H6b 2.90). After oxidization, the C-6 carbon of the sulfonate of the 6-deoxy-GlcNAc moiety in compound **12** had substantially shifted downfield (δ 40.5 → δ 52.5), and the corresponding protons also exhibited a chemical shift difference (H6a 3.33 and H6b 2.90 → H6a 3.41 and H6b 3.07).

### Enzymatic Synthesis of Oligo-LacNAc Mimics

The sulfonate methodology was also exploited for the preparation of oligo-LacNAc derivative **22**. In this case, the GlcNAc moiety modified by a disulfide at C-6 was carried through several enzymatic steps and then unmasked to give a thiol that could be oxidized to give the corresponding sulfonate (Scheme 4). Thus, tetrasaccharide **17** was prepared starting from chemically synthesized LacNAc having a benzyloxycarbonyl (CBz)-protected amino pentenyl linker at the anomeric center by the consecutive action of B3GnT2 and B4GalT1.^41^ The GlcNAc-6-S-SMe moiety of compound **2** was readily transferred to acceptor **17** by B3GnT2, and the resulting pentasaccharide **18b** could be extended with a β­(1,4)-Gal moiety by B4GalT1 in the presence of UDP-Gal to provide hexasaccharide **19**. The latter compounds, having a protected 6-thiol at GlcNAc, could readily be sialylated by ST3Gal4 resulting in the formation of sialoside **20**, which was then exposed to FUT5 and GDP-Fuc to give trifucoside **21**. These transformations show that 6-thiol-protected GlcNAc can readily be modified by B4GalT1, ST3Gal4, and FUT5 to install β1,4-galactose, α2,3-sialic acid, and α1,3-fucose, respectively. Finally, compound **21** was subjected to dithiothreitol (DTT) to reveal a thiol, which in the same pot was oxidized by oxone to give sulfonate **22**. Compound **22** was purified by size-exclusion column chromatography over Bio-Gel P2 using 50 mM NH_4_HCO_3_ buffer as the eluent. Finally, the Cbz-protecting group was removed by hydrogenation over Pd­(OH)_2_ to give the corresponding aminopentenyl derivative **23**.

**Scheme 4:**
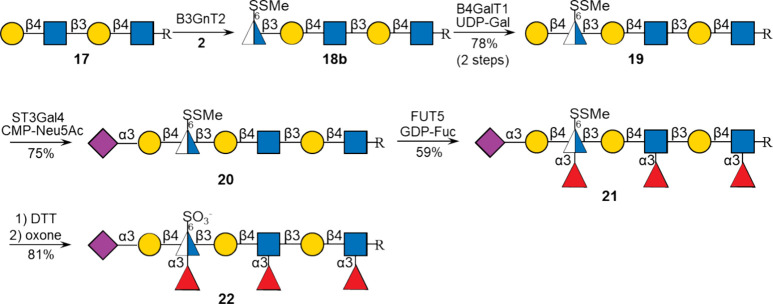
Enzymatic Synthesis of Linear Glycan Mimics^a^

a R= O­(CH_2_)_5_NHCbz.

### Glycan Microarray Development and Screening

To establish molecular recognition of the sulfonates, the newly synthesized glycans (**F**, **I**, **L**, **N**, and **Q**) and several reference compounds (**A**–**E**, **G, H**, **J, K, M**, **O**, and **P**), which include *N*- and *O*-glycans presenting relevant epitopes, were printed on succinimate-activated glass slides to construct a glycan microarray. The array was probed by several plant lectins (Figure 2b), Siglecs (Figure 2c), and recombinant hemagglutinins (HAs) derived from a variety of influenza A viruses isolated from ducks (Figure 2d,e).

**Figure 2:**
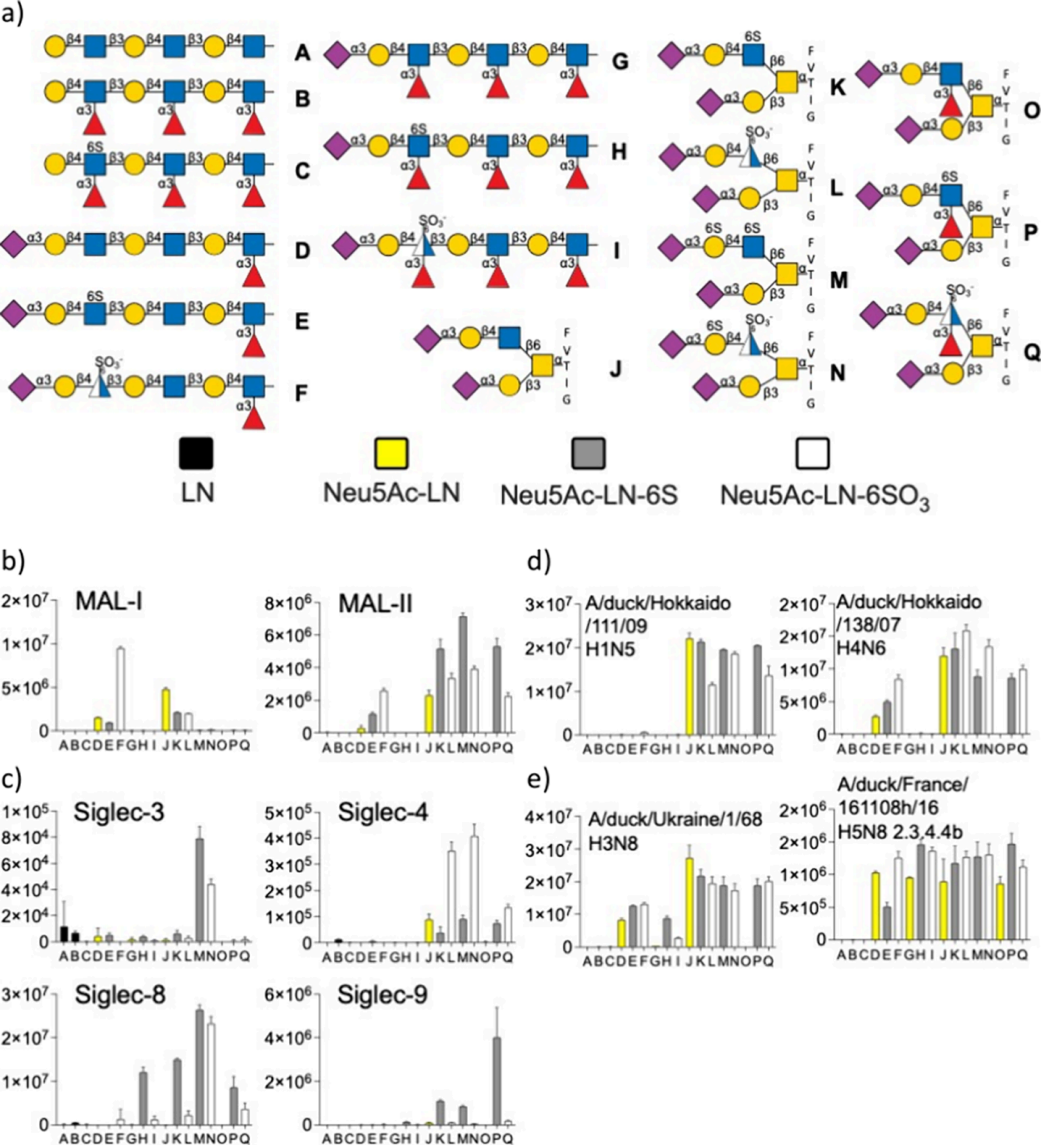
Probing glycan binding properties of lectins, glycan binding proteins, and influenza A hemagglutinins. (a) Collection of glycans printed on succinimide-reactive microarray slides. Glycan binding data of (b) lectins, (c) Siglecs, and (d, e) HAs of various influenza viruses. Bars represent the background-subtracted average relative fluorescence units (RFU) of four replicates ± SD.

As expected, MAL-I and MAL-II bound 2,3-sialylated LacNAc structures stronger than the 6-GlcNAc sulfated and sulfonated analogues (**D**–**F** and **J**–**L**). Fucosyl substitutions inhibited MAL-I binding. 6-Galactosyl sulfation was also accepted by MAL-II. For both the MAL-I and MAL-II lectins, sulfonylation substantially increased binding in extended SLN structures and was well tolerated in the shortest structures. In case of MAL-I, sulfation at GlcNAc (**E** and **K**) abrogated responsiveness, whereas it was tolerated by MAL-II. MAL-I preferred the linear structure **F** and glycopeptide **L** displaying a sulfonate. On the other hand, MAL-II bound sulfate and sulfonate-containing structures with similar responsiveness but did not recognize linear structures having multiple fucosides (**G**–**I**) as for MAL-I.

Siglecs are found on immune cells and can bind sialic acid-containing glycoconjugates^8^ to distinguish self from nonself. The Siglec–sialoside axis has been implicated in many diseases such as cancer, autoimmunity, and allergies.^42^

Siglec-3 (CD33) plays a role in regulating microglial activation, but in Alzheimer’s disease, its activation is increased because of the accumulation of amyloid and tau proteins. Siglec-3 displays substantial responsiveness only to two structures (**M** and **N**) that are derived from *O*-glycans. Compound **M** has sulfates on both Gal and GlcNAc and is a reported ligand for Siglec-3.^41^ Interestingly, the 6-sulfonate analogue **N** showed similar binding. Siglec-4, which is also known as myelin-associated glycoprotein (MAG), is exclusively expressed by myelinating cells of the nervous system. Unlike other Siglecs, MAG is not involved in immune regulations and inhibits neurite outgrowth and collapses axonal growth cones in a sialic acid binding-dependent manner.^43^ We observed Siglec-4 binding to control nonsulfated glycopeptides **J**, and sulfated glycopeptides **K**, **M**, and **P**, which is consistent with earlier observations that Siglec-4 has a binding preference for sialylated and sulfated O-glycans.^44^ Unexpectedly, a substantially greater responsiveness for glycans **L**, **N**, and **Q** was observed. The marked preference for the unnatural sulfonated compounds represents an attractive opportunity in the development of Siglec-4 inhibitors. Siglec-8 is reported to require a sulfate on GlcNAc and tolerates sulfation of Gal.^41,45^ Consistently, our glycan microarray reports binding to GlcNAc sulfated and GlcNAc and Gal sulfated structures, compounds **H**, **K**, **M**–**N**, and **P**-**Q**. Comparison of the Siglec-8 binding profile between **M** and **N**, and between **P** and **Q**, indicates that sulfonate-containing compounds bind equally well to their natural counterparts. It has been reported that Siglec-9 binds sulfated sialyl Lewis^x^ and sialyl-T and -Tn structures.^46^ On the array, this protein showed an exquisite specificity for the sulfated compounds **K**, **M**, and **P**, over the unnatural sulfonated counterparts, compounds **L**, **N**, and **Q**.

HA of influenza A viruses binds sialoglycans of the host for cell attachment and entry. Avian viruses preferentially bind 2,3-linked sialic acids, which are found in the duck enteric and chicken upper respiratory tract. On the other hand, human influenza A viruses (IAVs) recognize α2,6-linked sialic acids, which are predominantly found in the upper respiratory tract of humans. A notion is emerging that the specificity of HA for glycans is much more complex and that modifications such as fucosylation can influence receptor specificity, which can impact the host range and pathogenesis.^47–49^ We used the glycan microarray to examine binding selectivities of several recombinant HAs that are derived from viruses infecting avian and other species including H1N5, H4N6, H3N8, and H5N8. We employed mammalian cells expressing recombinant HA trimers, which were precomplexed with an antibody to the strep-tag and another one to the human Fc of the primary antibody.^50^

The HA of A/duck/Hokaido/11/09 (H1N5) bound only glycopeptide compounds (**J**–**N** and **P**–**Q**), which have a sialyl LacNAc moiety at the β1,6-arm that can be further sulfated or sulfonated. Fucosylation of sialyl LacNAc to yield sialyl Lewis^x^ (**O**) abolished binding while further sulfation or sulfonylation of SLe^x^ restored responsiveness. Sulfation of Gal as in compounds **M** and **N** is tolerated. The HA of another avian virus, A/duck/Hokaido/138/07 (H4N6), exhibited broader binding selectivity and recognized several oligo-LacNAc derivatives having a 2,3-sialyl-LacNAc moiety (**D**) as well as the corresponding sulfated (**E**) and sulfonated (**F**) derivatives. In the context of these compounds, fucosylation (**G**, **H**, and **I**) was not tolerated. A/duck/Ukraine/1/68 (H3N8) gave a similar binding pattern as H4N6; however, this HA recognized oligo-LacNAc derivatives having a sulfo-sialyl Lewis^x^ (**H**) and the corresponding sulfonated derivative (**I**). H5 viruses of the 2.3.4.4b subtype, which are currently in circulation, have a remarkable ability to infect many mammalian species, and there are indications that these H5 viruses prefer sulfated sialosides.^51^ The 2.3.4.4b representative A/duck/France/161108*h*/16 bound all sialylated structures and has the broadest receptor specificity of the examined HAs, which may explain its broad host range.

Collectively, microarray screening indicates that sulfonate-modified glycans can be recognized by many glycan binding proteins. In case of MAL-II, Siglecs-3 and 8, and several HAs, sulfonates display similar binding compared to the sulfate in binding preference. A surprising finding is that a sulfonate can increase the level of binding for Siglec-4.

### Molecular Dynamic Simulations of the Binding of Siglec-4 to Sulfate- and Sulfonate-Modified Sialyl LacNAc

Molecular dynamics (MD) simulations were performed of complexes of Siglec-4 with sulfate- and sulfonate-modified sialyl LacNAc to unravel the molecular basis by which the sulfonate of **L** and **N** enhances the binding to Siglec-4 compared to the sulfated counterparts **K** and **M**. The MD-derived models (Figure 3) recapitulated key intermolecular interactions observed in the X-ray crystal structure of Siglec-4 in complex with Neu5Acα­(2,3)­Galβ­(1,4)­GlcNAc.^43^ Specifically, both the sulfated and sulfonated trisaccharides bind in the sialic acid binding site that is formed by the CC′ loop and the F and G β-strands of the *N*-terminal V-type Ig1 domain.^52,53^ The sialic acid residue interacts with the protein via conserved arginine in the F β-strands (R118) by establishing a salt bridge between the carboxylate group of Neu5Ac and the guanidinium group of arginine. In β-strand G, the backbone carbonyl of Q126 forms a hydrogen bond with the amide of Neu5Ac, while T128 engages the sialic acid by establishing two additional hydrogen bonds with OH-9 and OH-8. Finally, Y127 provides van der Waals contact with the glycerol chain (C7–C9) of sialic acid. In the CC′ loop, Y65 accommodates the galactosyl ring through CH-pi interactions and a hydrogen bond of O9 of the Neu5Ac. Previous studies have documented a key role of the CC′ loop in modulation of ligand binding of the Siglec family of proteins.^54–56^ In the case of Siglec-4, the CC′ loop can adopt several conformations in unliganded crystal forms.^43^ After ligand binding, this loop adopts a single conformation. The amino acid of position 67 of the CC′ loop is occupied by lysine. The MD simulations reveal that this positively charged residue is properly placed to establish a salt bridge with the sulfate and sulfonate at C-6 of the GlcNAc residue (Figure 3). Since this lysine moiety is part of a flexible loop, it is well adapted for interaction with both the sulfate and the sulfonate. The MD trajectory confirms this electrostatic interaction is very stable and is present during the entire simulation. To shed light onto the binding preference for the sulfonated structure, as demonstrated by glycan microarray experiments, we examined charge properties of the two ligands by density functional theory (DFT). It revealed that replacement of oxygen by carbon results in a higher negative charge on the remaining oxygens of the sulfonate (Supporting Information Figure S4). This difference likely translates to stronger electrostatic interactions with Lys67 resulting in stronger binding.

**Figure 3:**
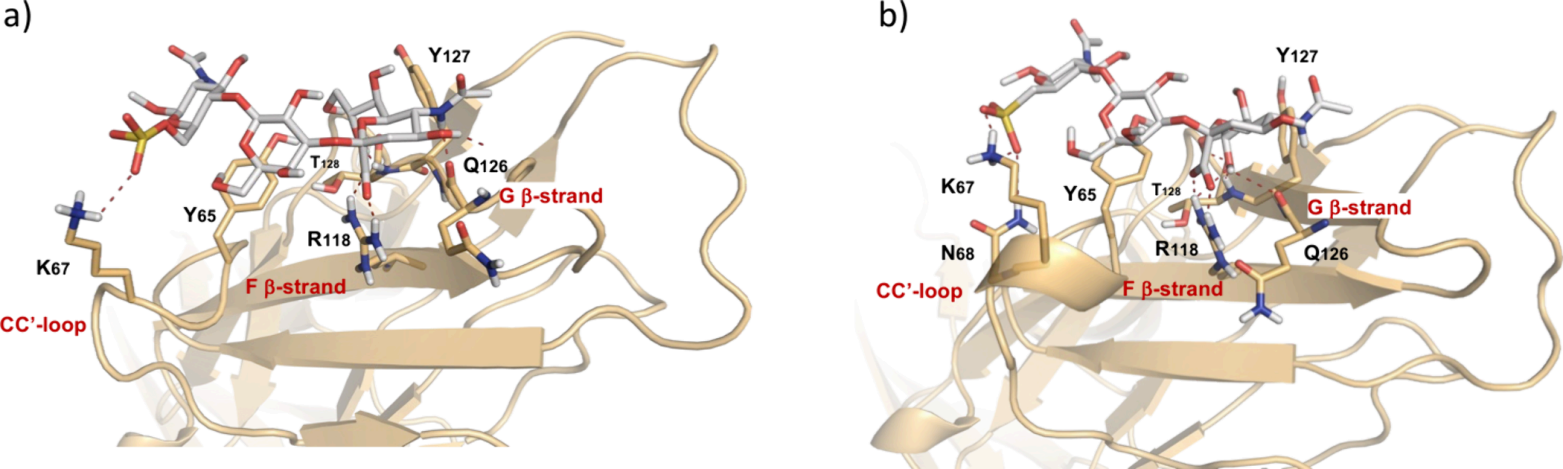
Structural characterization of ligand recognition by N-terminal domain Ig1 of Siglec-4. (A) Binding pose for the interaction of the sulfated Neu5Acα(2→3)­Galβ(1→4)­GlcNAc­(6-SO_3_^–^)­β–OH trisaccharide and for the sulfonated isostere Neu5Acα(2→3)­Galβ(1→4)­GlcNAc­(6-deoxy-SO_3_^–^)­β–OH, as derived from MD simulations.

## Conclusions

A concise and scalable synthetic route for UDP-GlcNAc analogues having a C-6-thiol or corresponding disulfides has been developed. It was found that GCNT1 can employ UDP-GlcNAc-6-deoxy-6-SH (**1**) as a donor to give, after oxidation of the thiol of the newly introduced GlcNAc moiety, entry into glycopeptide mimetics having a sulfonate moiety. Interestingly, B3GNT2 prefers compound **2** as a donor having at C-6 an S-SMe moiety, and the resulting oligosaccharides could be converted into sulfonates by reduction of the disulfide to give a thiol followed by oxidation. Furthermore, it was found that the sulfonate- and disulfide-containing oligosaccharides are appropriate acceptors for various glycosyltransferases, making it possible to prepare a range of glycomimetics. The sulfonates and several reference oligosaccharides and glycopeptides were printed as a glycan microarray, which uncovered binding selectivities of several lectins, glycan binding proteins, and HAs from influenza A viruses. It was found that a sulfonate can either be tolerated, be reduced, or increase binding compared to the corresponding sulfates. The structure-binding relationships highlight that a 6-sulfonate can modulate binding selectivities. Molecular modeling studies of the complex of Siglec-4 with sulfated and sulfonated sialyl LacNAc indicate that the plasticity of the binding site of the protein and a great charge on oxygens of a sulfonate is responsible for higher binding affinity. The compounds have an anomeric aminopentyl linker, which makes it possible to present them at dendrimers or nanoparticles to give multivalent derivatives that will bind with high avidity to a target protein and may serve as a lead compound for therapeutic applications. Introduction of a 6-sulfonate gives better step economy than conventional enzymatic sulfation. The sulfonates are resistant to hydrolysis by sulfatases and can modulate binding selectivities, making this compound attractive for the development of glycomimetics.

## Methods

### General Protocol for Enzymatic Reactions, Monitoring, and Product Purification

A reaction mixture composed of enzyme, additives, glycosyl acceptor, and sugar nucleotide in the appropriate buffer was incubated overnight at 37 °C with gentle shaking. The progress of the reaction was monitored by MALDI-TOF MS or ESI-TOF MS, and if starting material remained after 18 h, another portion of enzyme was added until no starting material was detected. The reaction mixture was centrifuged over a Nanosep Omega ultrafiltration device (10 kDa MWCO) to remove proteins, and the filtrate was lyophilized. The residue was applied to P2 or P6 size-exclusion column chromatography using Milli-Q water as the eluent, providing the desired product. High-performance liquid chromatography (HPLC) using an HILIC column (see the Methods section in the SI) was employed when impurities were detected.

### General Procedure for the Installation of β1,6 6-Deoxy-6SH-GlcNAc Using GCNT1

The glycosyl acceptor (1 equiv) and UDP-6-deoxy-6SH-GlcNAc (**1**, 1.5 equiv) were dissolved to provide a final acceptor concentration of 2–5 mM in a HEPES buffer (50 mM, pH 7.3) containing KCl (25 mM), MgCl_2_ (10 mM), and DTT (1 mM). Calf intestine alkaline phosphotase (CIAP, 1% total volume, 1 kU mL^–1^) and GCNT1 (1% w/w relative to acceptor substrate) were added. The general protocol for enzymatic reaction, monitoring and product purification was applied to give the required compound.

### General Procedure for the Installation of β1,3 6-deoxy-6SSMe-GlcNAc using B3GnT2

Glycosyl acceptor (1 equiv) and UDP-6-deoxy-6SSMe-GlcNAc (**2**, 1.5 equiv) were dissolved to provide a final acceptor concentration of 2–5 mM in a HEPES buffer (50 mM, pH 7.3) containing KCl (25 mM), MgCl_2_ (10 mM), and DTT (1 mM). Calf intestine alkaline phosphotase (CIAP, 1% total volume, 1 kU mL^–1^) and B3GnT2 (1% w/w relative to acceptor substrate) were added. The general protocols for enzymatic incubation, reaction monitoring, and product purification were subsequently applied.

### General Procedure for the Installation of β1,3-GlcNAc Using B3GnT2

The glycosyl acceptor (1 equiv) and UDP-GlcNAc (1.5 equiv) were dissolved to provide a final acceptor concentration of 2–5 mM in a HEPES buffer (50 mM, pH 7.3) containing KCl (25 mM), MgCl_2_ (10 mM), and DTT (1 mM). Calf intestine alkaline phosphotase (CIAP, 1% total volume, 1 kU mL^–1^) and B3GnT2 (1% w/w relative to acceptor substrate) were added. The general protocol for enzymatic reaction, monitoring, and product purification was applied to give the required compound.

### General Procedure for the Installation of a β1,4-Galactoside Using B4GalT1

The glycosyl acceptor (1 equiv) and UDP-Gal (1.5 eq per Gal) were dissolved to provide a final acceptor concentration of 2–5 mM in a Tris buffer buffer (100 mM, pH 7.5) containing MnCl_2_ (10 mM) and BSA (1% total volume). CIAP (1% volume total) and B4GalT1 (1% w/w relative to acceptor substrate) were added. The general protocol for enzymatic reaction, monitoring, and product purification was applied to give the required compound.

### General Procedure for the Installation of a β1,4-Galactoside Using B4GalT4

The glycosyl acceptor (1 equiv) and UDP-Gal (1.5 eq per Gal) were dissolved to provide a final acceptor concentration of 2–5 mM in a Tris buffer buffer (100 mM, pH 7.5) containing MnCl_2_ (10 mM) and BSA (1% total volume). CIAP (1% volume total) and B4GalT4 (1% w/w relative to acceptor substrate) were added. The general protocol for enzymatic reaction, monitoring, and product purification was applied to give the required compound.

### General Procedure for the Installation of α2,3-Neu5Ac Using ST3Gal1

The glycosyl acceptor (1 equiv) and CMP-Neu5Ac (1.5 equiv) were dissolved at a final acceptor concentration of 2–5 mM in a MOPS buffer (50 mM, pH 7.2) containing BSA (1% total volume). CIAP (1% volume total) and ST3Gal1 (1% w/w relative to acceptor substrate) were added. The general protocol for enzymatic reaction, monitoring, and product purification was applied to give the required compound.

### General Procedure for the Installation of α2,3-Neu5Ac Using ST3Gal4

The glycosyl acceptor (1 equiv) and CMP-Neu5Ac (1.5 equiv) were dissolved at final acceptor concentrations of 2–5 mM in a MOPS buffer (50 mM, pH 7.2) containing BSA (1% total volume). CIAP (1% volume total) and ST3Gal4 (1 wt %/wt relative to acceptor substrate) were added. The general protocol for enzymatic reaction, monitoring, and product purification was applied to give the required compound.

### General Procedure for α1,3-Fucosylation Using FUT5

The glycosyl acceptor (1 equiv) and GDP-Fuc (1.5 eq per GlcNAc to be added) were dissolved at a final acceptor concentration of 2–5 mM in a Tris-buffered solution (50 mM, pH 7.3) containing MnCl_2_ (10 mM). CIAP (1% total volume) and FUT5 (1% w/w) were added. The general protocol for enzymatic reaction, monitoring, and product purification was applied to give the required compound.

### General Procedure for α1,3-Fucosylation Using FUT6

The glycosyl acceptor (1 equiv) and GDP-Fuc (1.5 eq per GlcNAc to be added) were dissolved at final acceptor concentrations of 2–5 mM in a Tris-buffered solution (50 mM, pH 7.3) containing MnCl_2_ (10 mM). CIAP (1% total volume) and FUT6 (1% w/w) were added. The general protocol for enzymatic reaction, monitoring, and product purification was applied to give the required compound.

### General Procedure for 6-*O*-Sulfation of an Internal Galactoside Using CHST1

The glycosyl acceptor (1 equiv) and PAPS (1.6 equiv per galactose) were dissolved at final acceptor concentrations of 2–5 mM in a Tris-buffered solution (100 mM, pH 7.5) containing MgCl_2_ (10 mM). CHST1 (10% w/w relative to acceptor substrate) was added, and the reaction mixture was incubated overnight at 37 °C with gentle shaking. The reaction mixture was centrifuged over a Nanosep Omega ultrafiltration device (10 kDa MWCO) to remove proteins, and the filtrate was lyophilized. The residue was applicated to P2 or P6 size-exclusion column chromatography using aqueous NH_4_HCO_3_ (50 mM) as eluent providing the desired product. High-performance liquid chromatography (HPLC) using a HILIC column (see Methods) was employed when impurities were detected.

### General Procedure for the Oxidation of Thiol to Sulfonate

A thiol was dissolved in a solution of Tris buffer (50 mM, pH 7.5) to give a final concentration of 10 mM. The mixture was vortexed until all solids dissolved, and 1 eq of dithiothreitol (4.5 μL, 1 M) was added in one portion. After 10 min, 5 equiv of oxone (225 μL, 100 mM) was added. The reaction mixture was kept at 37 °C with gentle shaking. When full conversion was observed by ESI-TOF-MS, the reaction mixture was lyophilized. Purification by P6 size-exclusion column chromatography then HILIC-HPLC provided the desired product **12**.

### General Procedure for the Conversion of SSMe to Sulfonate Using DTT and Oxone

Intermediate **21** or **24** (1 equiv) was dissolved in a solution of Tris buffer (50 mM, pH 7.5) to give a final concentration of 1 mM. The mixture was vortexed until all solids dissolved, and then dithiothreitol (10 eq, 1 M aqueous solution) was added in one portion. When full conversion of SSMe to SH was observed by ESI-TOF-MS, oxone (10 equiv, 100 mM aqueous solution) was added. The reaction mixture was kept at 37 °C with gentle shaking. When full oxidation was observed by ESI-TOF-MS, the reaction mixture was lyophilized. The residue was purification by P6 size-exclusion column chromatography providing the desired product **22** or **25.**

### General Procedure for Cbz Deprotection

Palladium hydroxide on carbon (Degussa type, 20%, 1.5 times the weight of starting material) was added to a solution of compound **22** or **25** in H_2_O containing 0.1% AcOH as an additive. The mixture was placed under an atmosphere of hydrogen until completion of the reaction, as indicated by ESI-LC-MS. The mixture was filtered through a cotton filter, and the residue was washed with H_2_O. The filtrate was lyophilized to give the final product. P6 size-exclusion column chromatography was used for purification with 50 mM ammonium bicarbonate as eluent, and the fractions containing compound were lyophilized to give the desired compound **23** or **26** as a white powder.

### Computational Approaches

The 3D structures of the sulfated Neu5Acα­(2,3)­Galβ­(1,4)­GlcNAc­(6-SO_3_^–^)­β–OH and of the sulfonated isostere Neu5Acα­(2,3)­Galβ­(1,4)­GlcNAc­(6-Deoxy-SO_3_^–^)­β–OH were built using the Maestro Schrödinger suite of program Maestro version 14.2.118 and energy minimized by the Macromodel minimization tool using the OPLS4 force field in water with an extended cutoff. Structural parameters, such as atomic charges, bond length, and angles, were calculated in Gaussian 09 program, using the HF/6-31G­(d) potential. The crystal structure of Siglec-4 in complex with Neu5Acα­(2,3)­Galβ­(1,3)­GalNAcβ trisaccharide has been reported (pdb code 5lf5).^43^ The 3D structures of the sulfated and sulfonated sialyl LacNAc were placed into the binding pocket of Siglec-4 by molecular alignment with the trisaccharide of the cocrystal structure. The resulting binding poses were energy minimized and subjected to MD simulation in explicit water by using the AMBER molecular simulation program (AMBER 2024). Given the presence of the unnatural sulfonate moiety, the general gaff force field was employed for the glycans, while the 14SB force field was used for the protein. Finally, the water tip 3p model was used to simulate the boundary solvent and ion conditions. Two consecutive minimization stages were performed involving (1) only the water end ions and (2) the whole system using the steepest descent algorithm. The system was subjected to heating and equilibration before the real dynamic simulation. Molecular dynamics (MD) simulations were run at a constant temperature of 300 K and 1 atm pressure. 500 ns molecular dynamics simulations without constraints were recorded, using an NPT ensemble with periodic boundary conditions, a cutoff of 10 Å, and the particle mesh Ewald method. A total of 50,000,000 molecular dynamics steps were run with a time step of 1 fs per step. Coordinates and energy values were recorded every 100,000 steps for a total of 500 MD models. The detailed analysis of the intermolecular hydrogen bonds (HB) and electrostatic and hydrophobic interactions was performed along the MD trajectories using the cpptraj module included in the AmberTools 24 package.

## Supplementary Material



## Notes

Synthetic protocols, compound characterization, experimental procedure for enzyme and protein expression, microarray screening, computational approaches, and copies of NMR spectra (PDF)
