# Supplementary material for: Chemoenzymatic Synthesis and Biological Recognition of a Sulfonate Isostere of 6‑Sulfo-sialyl Lewisx
Source: JACS Au. 2025 Jun 15;5(7):3328–38. doi: 10.1021/jacsau.5c00443 (PMC12308420; doi:10.1021/jacsau.5c00443)
Supplement: Supplementary file 1 [file au5c00443_si_001.pdf]

Supplementary Information

Chemoenzymatic Synthesis and Biological Recognition of a Sulfonate Isostere of 6-Sulfo-Sialyl Lewis^{x}

Yunfei Wu, Julia Weber, Anne L.M. Kimpel, Luca Unione,
Elif Uslu, Robert P. de Vries, and Geert-Jan Boons*

Table of Contents

page

1) Materials and Methods ……………………………………..…… S3

2) Synthesis and Analysis ……………………………………..…... S7

3) Microarray Procedure ………………………………………….. S31

4) Molecular Dynamic Simulations ……………..………………… S33

5) References ……………………………………………...……. S34

6) NMR Spectra ……………………………………..………….. S36

1) Materials and Methods

Glycosyltransferases B3GnT2 and Hpβ3GlcNAcT, B4GalT1 and B4GalT4, ST3Gal1 and ST3Gal4, CHST1, GCNT1, FuT5 and FuT6 and FuT9 were expressed and purified according to published protocols.[1-5] Reagents were purchased from Sigma-Aldrich. Uridine 5'-diphosphogalactose (UDP-Gal), uridine 5'-diphospho-N-acetyl-glucosamine (UDP-GlcNAc) and cytidine-5'-monophospho-N-acetylneuraminic acid (CMP-Neu5Ac) were obtained from Roche Diagnostics [UDP-Gal: Cat# 07703562103; UDP-GlcNAc: Cat# 06369855103; CMPNeu5Ac: Cat# 05974003103]. Adenosine 3′-Phosphate 5′-Phosphosulfate (PAPS) were obtained from Merck [Cat# 118410, Purity≥80% by HPLC]. GDP-Fucose was prepared using L-fucokinase/GDP-fucose pyrophosphorylase.[6] Progress of the reactions was monitored by liquid chromatography mass spectrometry system (LCMS) from Shimadzu (system controller: SCL10A-VP; HPLC pumps: LC10AD-VP; injector: SIL10AD-VP) using a ZIC HILIC column (ZeQuant, PEEK coated guard HPLC column, 3.5 μm particle size, 20x 2.1 mm). The LC system was attached to a Bruker Daltonics micro TOF-Q mass spectrometer. Mass spectra were recorded on either on an Applied Biosystems SCIEX MALDI TOF/TOF 5800 mass spectrometer, a Shimadzu Biotech Axima-CFR MALDI-TOF, or a high-resolution Shimadzu LCMS-IT-TOF mass spectrometer. Reaction mixtures were purified using a size exclusion Biogel (P2) or Biogel (P6) resins from BioRad in Econo glass columns (0.7 x 30 cm / 1.5 x 30 cm / 1.5 x 50 cm/ 1.5 x 120 cm) coupled to a BioFrac fraction collector (BioRad). Carbohydrate-containing fractions were detected by thin layer chromatography and an appropriate staining reagent (15 mL AcOH and 3.5 mL p-Anisaldehyde in 350 mL EtOH and 50 mL H_2SO_4). If needed, further purification was performed by HPLC-MS using a ZIC HILIC column.

Expression and Purification of Recombinant Human Glycosyltransferases and Sulfotransferases

Expression constructs were generated encoding the truncated catalytic domains of human glycosyltransferases (B3GnT2, B4GALT1, B4GALT4, FuT5, FuT6, FuT9, ST3GAL1, ST3GAL4 and GCNT1) and sulfotransferase CHST1 as NH_2-terminal fusion proteins in the pGEn2 expression vector essentially as described in prior studies.[4,7,8] Briefly, the fusion protein coding regions were comprised of a 25-amino acid signal sequence, an His_8 tag, AviTag, the "superfolder" GFP coding region, the 7-amino acid recognition sequence of the tobacco etch virus (TEV) protease followed by the respective catalytic domain regions (for human CHST1 (Uniprot ID: O43916) catalytic domain region comprising of 388 amino acid

residues). The recombinant human glycosyltransferases and sulfotransferases were expressed as a soluble secreted proteins by transient transfection of suspension culture HEK293-F cells (FreeStyle™ 293-F cells, Thermo Fisher Scientific, Waltham MA) and purified by Ni^{2+}-NTA chromatography as previously described.[7,8] Each protein was concentrated to approximately 3 mg/ml using an ultrafiltration pressure cell (Millipore, Billerica, MA) with a 10-kDa molecular mass cutoff membrane. The enzymes were further purified by gel filtration on a Superdex G-75 column (GE Healthcare) preconditioned with a buffer containing 20 mM HEPES, 150 mM NaCl, 0.05% Sodium azide, pH 7.0. Peak fractions of recombinant human enzymes were pooled, respectively, concentrated at 1 mg/ml and buffer exchanged with 20mM HEPES, 100 mM NaCl, 0.05% Sodium azide, pH 7.0, 10% glycerol. The final protein preparations were aliquoted and stored at -80 °C until use.

General Protocols for Enzymatic Reactions

General Procedure for the Installation of α1,3 Fuc using FUT9

Glycosyl acceptor (1 eq) was dissolved at a final acceptor concentration of 2 mM in a Tris buffered solution (50 mM, pH 7.3) containing $MnCl_2$ (10 mM). CIAP (1% total volume) and FUT9 (1% wt/wt) were added. Reaction progress was monitored by ESI-TOF MS. GDP-Fuc (0.9 eq per Fuc to be added) was added in 3 portions (each portion 0.3 eq, added after GDP-Fuc could not be detected) and the reaction mixture was incubated at 37 °C with gentle shaking. After the major starting material consumed, the reaction mixture was centrifuged over a Nanosep® Omega ultrafiltration device (10 kDa MWCO) to remove proteins, and the filtrate was lyophilized. The residue was applicated to P2 or P6 size-exclusion column chromatography using Milli-Q water as eluent, providing the desired product. High performance liquid chromatography (HPLC) using HILIC column (see materials) was employed when the impurities were detected after size exclusion.

General Protocols for HILIC-HPLC Purification

HILIC-HPLC Purification Conditions for Glycosyl Asparagine Containing Compounds

Semi-preparative HILIC-HPLC was applied on a Shimadzu (LC-20AT, SIL-20A, CBM-20A, SPD-20A, FRC-10A) LC-ESI-IT-TOF with a XBridge HILIC column, 5 µm, 10 x 250 mm at a flow rate of 3.6 mL/min, injection volume of 100 µL (10-20 mg/mL), with 0.2% of the flow diverted to the ESI-MS detector using a splitter. The purification was performed using 10% 10 mM NH_4HCO_3 in MeCN (buffer B) and MeCN in 80% 10 mM NH_4HCO_3 (buffer A). General conditions for a linear gradient were used as the eluent:

Time (min)	A (%)	B (%)
0	5	95
90	50	50

LC-MS data for *O*-glycopeptides 14-16

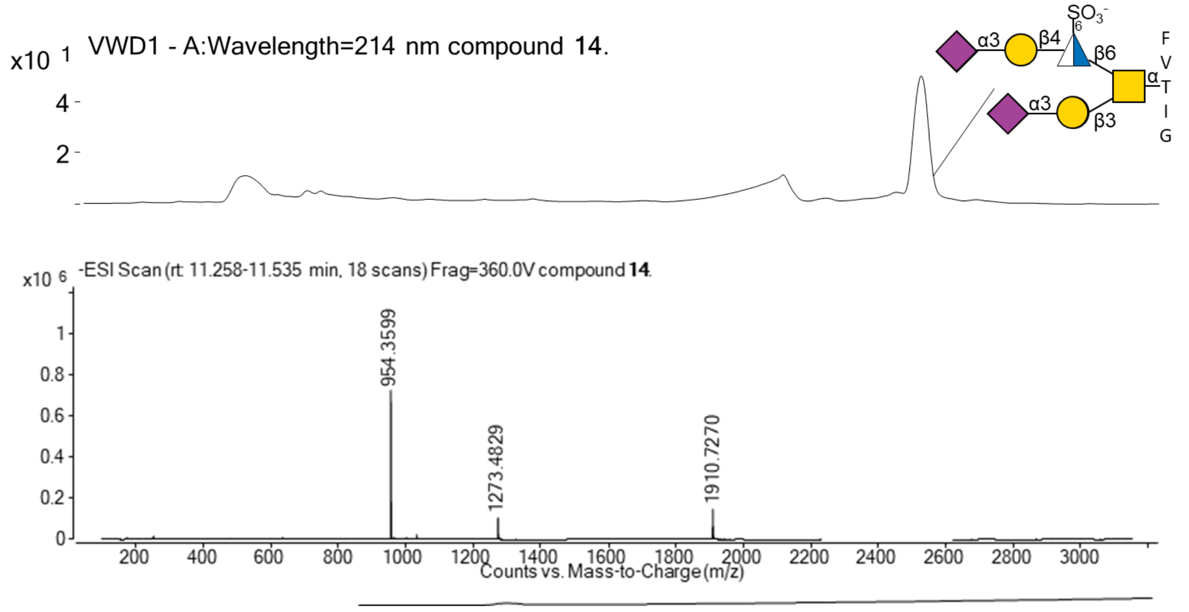

Figure S1. LC-MS profile of *O*-glycopeptide **14**.

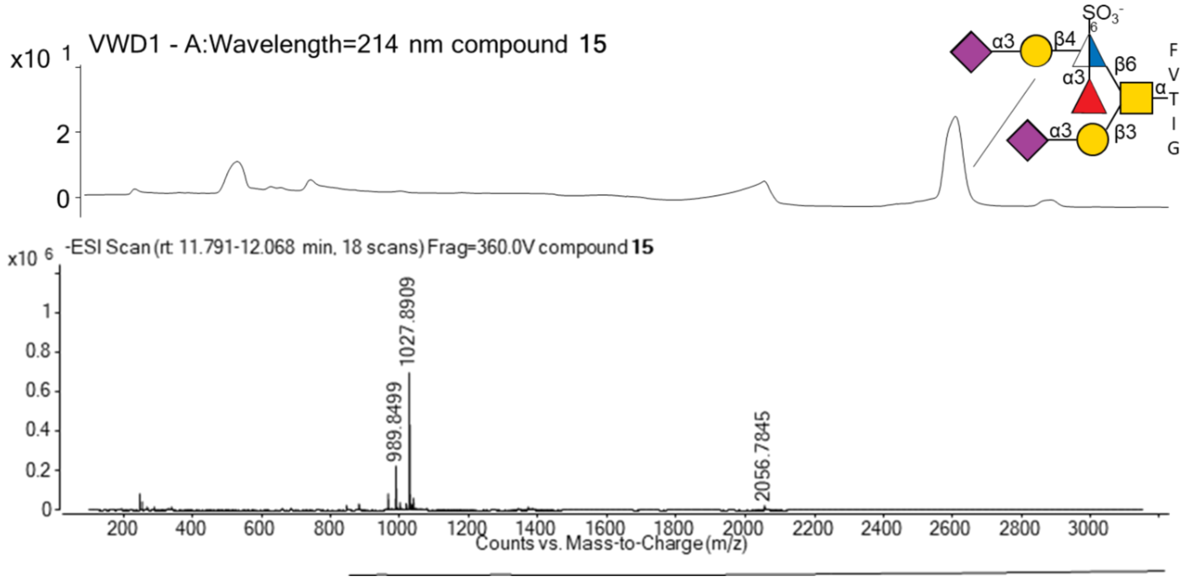

Figure S2. LC-MS profile of *O*-glycopeptide **15**.

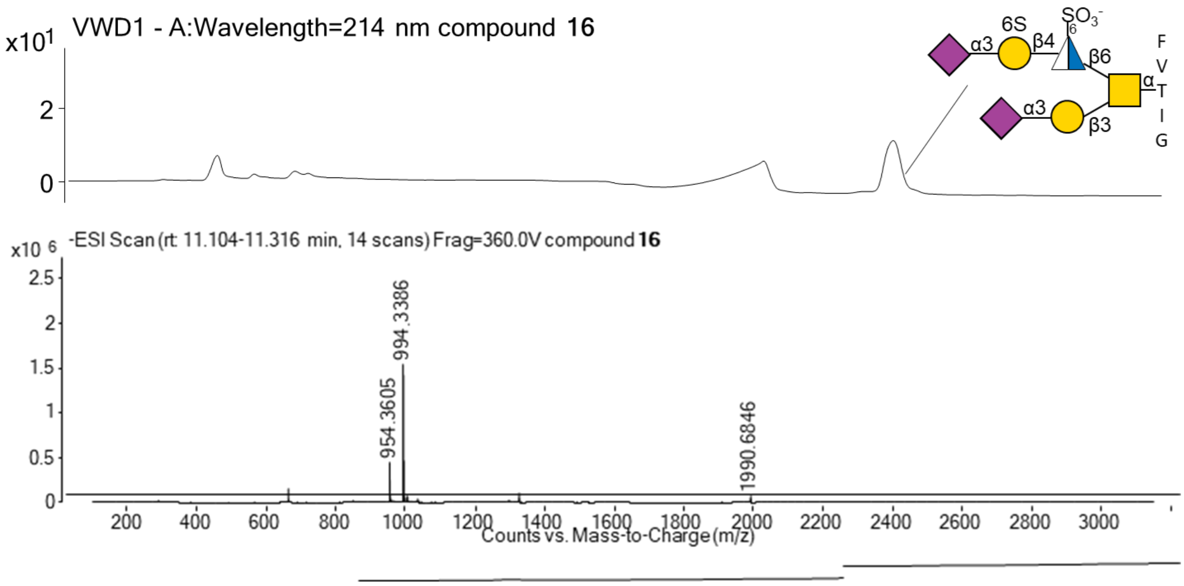

Figure S3. LC-MS profile of *O*-glycopeptide **16**.

2) Synthesis and Analysis

In chemical synthesis part, chemical shifts are reported in parts per million (ppm) relative to $CDCl_3$ as the internal standard. NMR data are presented as follows: Chemical shift, multiplicity (s = singlet, d = doublet, t = triplet, dd = doublet of doublets, m =multiplet and/or multiple resonances, app = apparent); coupling constants are reported in Hertz (Hz).

In enzymatic synthesis part, VnmrJ 4 and TopSpin 4 were used to collect NMR data. NMR data was obtained at room temperature on a 600 MHz instrument from Bruker. The chemical shift δ is given in parts per million (ppm) and refers to tetramethylsilane and the residual solvent peak [^{1}H-NMR: δ(D_2O) =4.79 ppm]. NMR data is given as follows: ^{1}H-NMR: chemical shift (multiplicity, coupling constants, relative integral, functional group); ^{13}C data are extracted from HSQC spectra and given as follows: chemical shift. Multiplicity is defined as follows: s = singlet; d = doublet; t = triplet; m= multiplet. Signals were assigned by numbering the monosacharide units starting at the reducing end of the oligosaccharide. The assignment was done by using corresponding 2D-NMR spectra (COSY, HSQC). The yield/concentration of the final products was determined by NMR spectroscopy, using n-propanol as an internal standard. High resolution masses were measured on an Agilent 6560 Ion Mobility Q-TOF LC-MS system.

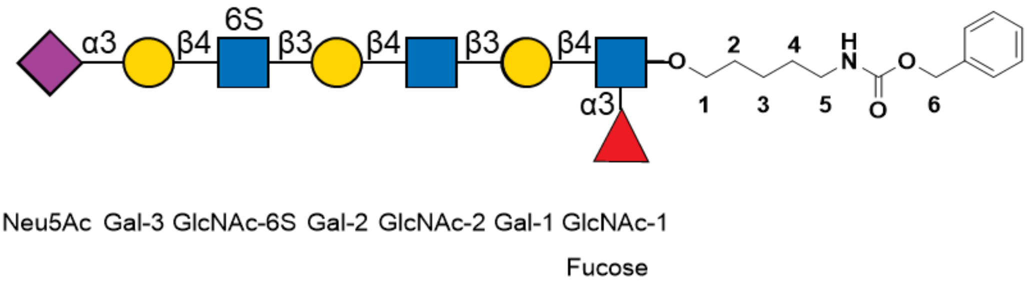

and

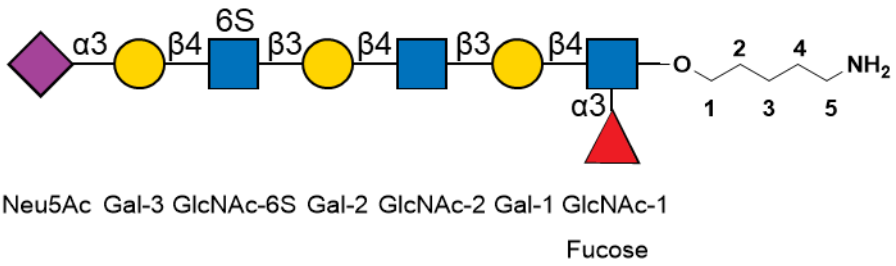

and

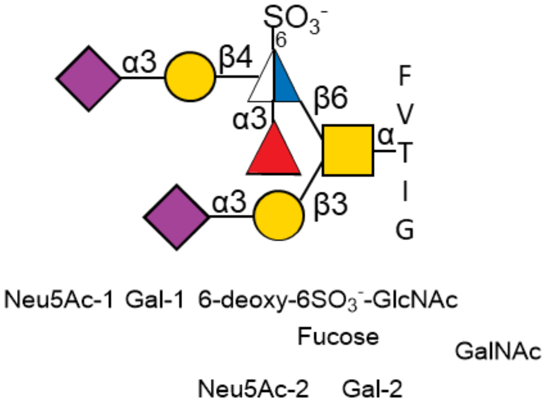

UDP-6-deoxy-6SR-GlcNAc Analogs Preparation

(3R,4R,5S,6R)-3-acetamido-6-((tosyloxy)methyl)tetrahydro-2H-pyran-2,4,5-triyl triacetate (5)

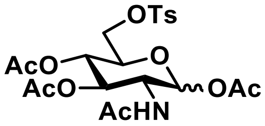

p-Toluene sulfonyl chloride (16.16 g, 84.8 mmol) in anhydrous pyridine (250 mL) was added dropwise over 30 min to a solution of 2-acetamido-2-deoxy-glucose **4** (15.0 g, 67.8 mmol) in dry pyridine (250 mL) at 0 °C. The reaction mixture was stirred at 0 °C for 8 h before acetic anhydride (125 mL) was added and the mixture stirred at RT overnight. The solvent was removed *in vacuo* and the residue was dissolved in ethyl acetate (200 mL), washed with 1 M HCl (2 x 200 mL) and saturated $NaHCO_3$ (2 x 200 mL). The organic fraction was dried over anhydrous Na_2SO_4 and concentrated *in vacuo*. The residue was purified by flash column chromatography on silica gel (ethyl acetate:petroleum ether = 4:1) to afford **5** as a white solid (29.97 g, 88%). Rf 0.35 (100% ethyl acetate). NMR data are as reported.[9]

(2R,3S,4R,5R)-5-acetamido-6-hydroxy-2-((tosyloxy)methyl)tetrahydro-2H-pyran-3,4-diyl diacetate (6)

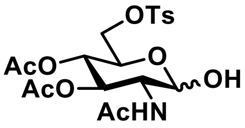

To a solution of **5** (6.59 g, 13.15 mmol) in anhydrous dimethylformamide (80 mL) was added hydrazine acetate (1.39 g, 15.12 mmol) and the resultant mixture was stirred at RT for 3 hours. The dimethylformamide was removed *in vacuo* and the residue dissolved in dichloromethane (100 mL) and washed with saturated $NaHCO_3$ (3 x 100 mL). The organic

fraction was dried over anhydrous Na_2SO_4, the solvent removed *in vacuo* and the residue purified by flash column chromatography on silica gel (ethyl acetate:petroleum ether = 4:1) to afford the desired hemiacetal **6** as a white solid (3.58 g, 60%). NMR data are as reported.[10]

(2R,3S,4R,5R,6R)-5-acetamido-6-((bis(benzyloxy)phosphoryl)oxy)-2-((tosyloxy)methyl)tetrahydro-2H-pyran-3,4-diyl diacetate (7)

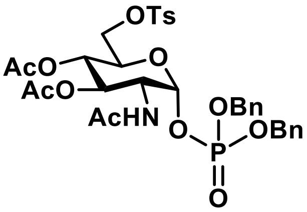

To a solution of a portion of this hemiacetal **6** (3.58 g, 7.79 mmol) in anhydrous dichloromethane (100 mL) was added triazole (1.091 g, 15.6 mmol) and the reaction mixture was cooled to -78 °C. After 5 min, dibenzyl N,N-di-iso-propylphosphoramidite (3.49 mL, 9.35 mmol) was added dropwise and the mixture was stirred at RT for a further 3 h. The mixture was cooled to -78 °C and mCPBA (4.80 g, 19.48 mmol) was added in one portion. The reaction mixture was allowed to warm to RT. After stirring for 2 h at RT the solvent was removed in vacuo and the residue was dissolved in ethyl acetate (200 mL), washed with ice cold saturated $Na_2S_2O_3$ (300 mL), ice cold saturated $NaHCO_3$ (2 x 300 mL) and ice-cold brine (300 mL). The organic layer was dried over $MgSO_4$, filtered, concentrated in vacuo and the residue purified by flash column chromatography on silica gel (ethyl acetate:petroleum ether = 7:3 to 1:0) to afford **7** as a white foam (4.1 g, 73% over 2 steps). Rf 0.44 (100% ethyl acetate). NMR data are as reported.[9]

(2R,3R,4R,5S,6S)-3-acetamido-4,5-diacetoxy-6-((acetylthio)methyl)tetrahydro-2H-pyran-2-yl phosphate (8)

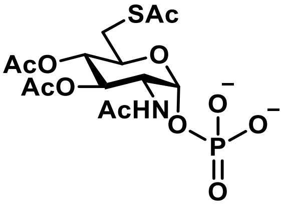

Phosphate **7** (2.63 g, 3.65 mmol) and 10% Pd/C (260 mg) were added to a dry flask. The flask was evacuated and re-charged with H_2 whilst cooling in dry ice. Anhydrous methanol (30 mL) was added. The reaction system was evacuated and charged with hydrogen three times. The reaction mixture was then stirred at RT under an atmosphere of hydrogen for 18 h.

After this time the reaction mixture was filtered through celite, and the filter plug washed with MeOH. The solvent was removed in vacuo to give the deprotected intermediate.

The intermediate was dissolved in dry CH_3CN (60 mL), potassium thioacetate (2.34 g, 20.50 mmol) added and the resultant mixture heated to 70 °C for 18 h. The solvent was removed in vacuo and the residue purified by flash column chromatography on silica gel (water: methanol: ethyl acetate = 1:2:4) to afford **8** (1.3 g, 80% over 2 steps) as a red foam. ^{1}H NMR (400 MHz, MeOD) δ 5.43 (dd, *J* = 6.9, 3.4 Hz, 1H, H-1), 5.26 (dd, *J* = 10.7, 9.3 Hz, 1H, H-3), 4.99 (t, *J* = 9.7 Hz, 1H, H-4), 4.40 (dt, *J* = 10.1, 3.8 Hz, 1H, H-5), 4.28 – 4.18 (m, 1H, H-2), 3.40 – 3.33 (m, 1H, H-6$_a$), 3.17 (dd, *J* = 14.5, 3.3 Hz, 1H, H-6$_b$), 2.31 (s, 3H, SCOC$\underline{H_3}$), 2.02, 1.94 (2xs, 6H, 2xOCOC$\underline{H_3}$), 1.93 (s, 3H, NHCOC$\underline{H_3}$). ^{13}C NMR from HSQC (100 MHz, MeOD) δ 94.91 (C-1), 72.66 (C-3), 71.09 (C-4), 69.78 (C-5), 53.07 (C-2), 30.12 (C-6), 30.04 (SCO$\underline{C}H_3$), 22.20 (NHCO$\underline{C}H_3$), 20.53, 20.36 (2xOCO$\underline{C}H_3$).

(2R,3R,4R,5S,6S)-3-acetamido-6-((tert-butyldisulfaneyl)methyl)-4,5-dihydroxytetrahydro-2H-pyran-2-yl phosphate (9)

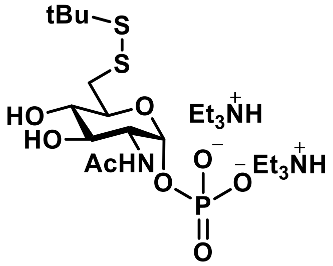

Compound **8** (1.56 g, 3.53 mmol) was dissolved in anhydrous methanol (80 mL) and the solution of MeONa in methanol was added to adjust pH to 11. Then, $tBuSSO_2Me$ (891.2 mg, 5.30 mmol) was added and stirred overnight. Adjust pH back to 7 with DOWAX H^+ resin. After this time the reaction mixture was filtered through celite and the filter plug washed with MeOH. The solvent was removed in vacuo and the residue purified by flash column chromatography on silica gel (water: methanol: ethyl acetate = 1:2:4 with 0.1% triethylamine as additive) to afford **9** (1.96 g, 91%) as a yellow foam. ^{1}H NMR (400 MHz, MeOD) δ 5.43 (dd, *J* = 7.2, 3.3 Hz, 1H, H-1), 4.05 (ddd, *J* = 9.5, 5.9, 3.1 Hz, 1H, H-5), 3.94 (dt, *J* = 10.5, 2.8 Hz, 1H, H-2), 3.67 (dd, *J* = 10.5, 8.8 Hz, 1H, H-3), 3.43 (t, *J* = 9.3 Hz, 1H, H-4), 3.32- 3.29 (m, 1H, H-6$_a$), 3.20 (q, *J* = 7.3 Hz, 6H, NH(C$\underline{H_2}$CH$_3$)$_3^+$), 3.09 (dd, J = 13.1, 5.9 Hz, 1H, H-6$_b$), 2.00 (s, 3H, NHCOC$\underline{H_3}$), 1.34-1.29 (m, 9H, NH(CH$_2$C$\underline{H_3}$)$_3^+$), 1.32 (s, 9H, SC(C$\underline{H_3}$)). ^{13}C NMR (100 MHz, MeOD) δ 173.86 (NH$\underline{C}$OCH$_3$), 95.55, 95.49 (d, C-1), 74.06 (C-4), 73.14 (C-5), 72.93 (C-3), 55.61, 55.54 (d, C-2), 47.74 (NH($\underline{C}$H$_2$CH$_3$)$_3^+$), 45.85 (C-6), 30.28

(SC(C̲H$_3$)), 22.83 (NHCOC̲H$_3$), 9.19 (NH(CH$_2$C̲H$_3$)$_3^+$). HRMS (ESI-MS): m/z calculated for $C_{12}H_{23}NO_8PS_2$ [M-H]$^-$: 404.0608; found: 404.0493.

UDP-6-deoxy-6SStBu-GlcNAc (3)

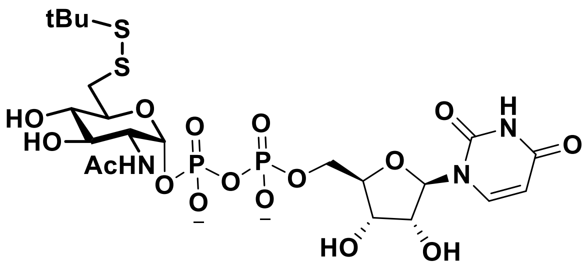

The yellow solid **9** (2.12 g, 3.5 mmol) was dissolved in dry pyridine and evaporated. This process was repeated two more times. Finally, the solid was left under high vacuum overnight to dry. The next day the sugar was dissolved in dry pyridine, UMP-morpholidate (4.66 g, 6.70 mmol) and tetrazole (1.17 g, 16.76 mmol) were added and the solvent was removed once again. The residue was dissolved in dry pyridine (24 mL) and stirred at room temperature. After 4 days, the solvent was removed, the residue dissolved in H_2O (120 mL) and washed with 2 x 100 mL of diethyl ether. The aqueous layer was then purified by flash column chromatography on silica gel (water: methanol: ethyl acetate = 1:2:4). The collected product was still contaminated with some unreacted starting material **9**. The lyophilized mixture was dissolved in Tris buffer (100 mM, pH 8, final volume of 20 mL) containing $MgCl_2$ (10 mM) and was incubated with alkaline pyrophosphatase (200 µL, 1 unit/µL) at 37 °C. All the unreacted starting material **9** was dephosphorylated in 6 h. The mixture was then chromatographed by flash column chromatography on silica gel (water: methanol: ethyl acetate = 1:2:4) to afford **3** (400 mg, 16 %) as a white solid. ^{1}H NMR (600 MHz, D_2O) δ 8.01 (d, *J* = 8.1 Hz, 1H, CO-CH=CH̲-N), 5.99 (d, *J* = 8.1 Hz, 1H, CO-CH̲=CH-N), 5.98 (d, *J* = 3.2 Hz, 1H, H-1^{UDP}), 5.49 (d, *J* = 7.0 Hz, 1H, H-1^{GlcNAc}), 4.39 – 4.34 (m, 2H, H-2^{UDP}, H-3^{UDP}), 4.31 – 4.28 (m, 1H, H-4^{UDP}), 4.28 – 4.18 (m, 2H, H-5a^{UDP}, H-5b^{UDP}), 4.14 (ddd, *J* = 9.7, 5.9, 2.8 Hz, 1H, H-5 ^{GlcNAc}), 4.01 (d, *J* = 10.5 Hz, 1H, H-2 ^{GlcNAc}), 3.80 (t, *J* = 9.8 Hz, 1H, H-3 ^{GlcNAc}), 3.57 (t, *J* = 9.5 Hz, 1H, H-4 ^{GlcNAc}), 3.34 – 3.27 (m, 1H, H-6a ^{GlcNAc}), 3.12 (dd, *J* = 13.9, 6.0 Hz, 1H, H-6b ^{GlcNAc}), 2.08 (s, 3H, COCH̲$_3$), 1.33 (s, 9H, SC(CH̲$_3$)). ^{13}C NMR (150 MHz, D_2O) δ 174.73 (C̲OCH$_3$), 166.25 (C̲O-CH=CH-N), 151.75 (NH-C̲O-N), 141.66 (CO-CH=C̲H-N), 102.68 (CO-C̲H=CH-N), 94.27 (C-1 ^{GlcNAc}), 88.63 (C-1 ^{UDP}), 83.10 (C-4 ^{UDP}), 73.93 (C-2 ^{UDP}), 71.74 (C-5 ^{GlcNAc}), 71.72 (C-4 ^{GlcNAc}), 70.71 (C-3 ^{GlcNAc}), 69.46 (C-3 ^{UDP}), 64.82 (C-5 ^{UDP}), 53.71 (C-2 ^{GlcNAc}), 48.07 (SC̲(CH$_3$)), 42.74 (C-6 ^{GlcNAc}), 28.97 (SC(C̲H$_3$)),

22.04 (CO$\underline{C}H_3$). HRMS (ESI-MS): m/z calculated for $C_{21}H_{34}N_3O_{16}P_2S_2$ $[M-H]^-$: 710.0861; found: 710.0811.

UDP-6-deoxy-6SH-GlcNAc (1)

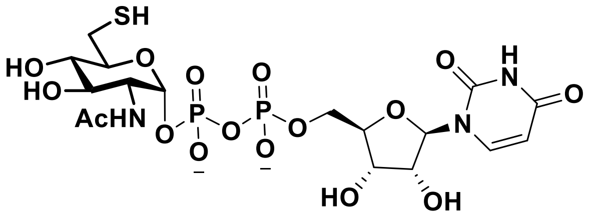

3 (94.1 mg, 0.13 mmol) dissolved in H_2O (3 mL) and TCEP bond breaker solution (317 µL, 0.50 M) were mixed and this mixture was stirred for 18 h. The solvent was removed in vacuo and the residue purified by flash column chromatography on silica gel (water: methanol: ethyl acetate = 1:2:5) to afford **1** (80.0 mg, 97%) as a white foam. ^{1}H NMR (400 MHz, MeOD) δ 7.99 (d, J = 8.1 Hz, 1H, CO-CH=C$\underline{H}$-N), 5.94 (d, J = 4.8 Hz, 1H, H-1^{UDP}), 5.82 (d, J = 8.1 Hz, 1H, CO-C$\underline{H}$=CH-N), 5.57 (d, J = 6.4 Hz, 1H, H-1^{GlcNAc}), 4.35 – 4.29 (m, 1H, H-3^{UDP}), 4.27 – 4.21 (m, 3H, H-2^{UDP}, H-5a^{UDP}, H-5b^{UDP}), 4.16 – 4.12 (m, 1H, H-4^{UDP}), 4.04 – 3.94 (m, 2H, H-2^{GlcNAc}, H-5^{GlcNAc}), 3.73 (t, J = 9.7 Hz, 1H, H-3^{GlcNAc}), 3.56 (t, J = 9.3 Hz, 1H, H-4^{GlcNAc}), 2.96 (dd, J = 14.1, 2.9 Hz, 1H, H-6a^{GlcNAc}), 2.76 (dd, J = 14.1, 4.8 Hz, 1H, H-6b^{GlcNAc}), 2.05 (s, 3H, COC$\underline{H_3}$). ^{13}C NMR from HSQC (100 MHz, MeOD) δ 142.39 (CO-CH=$\underline{C}$H-N), 102.93 (CO-$\underline{C}$H=CH-N), 96.37 (C-1^{GlcNAc}), 89.89 (C-1^{UDP}), 84.85 (C-4^{UDP}), 75.53 (C-2^{UDP}), 73.63 (C-5^{GlcNAc}), 72.69 (C-4^{GlcNAc}), 72.50 (C-3^{GlcNAc}), 70.98 (C-3^{UDP}), 66.07 (C-5^{UDP}), 55.30 (C-2^{GlcNAc}), 26.63 (C-6^{GlcNAc}), 22.76 (CO$\underline{C}H_3$). HRMS (ESI-MS): m/z calculated for $C_{17}H_{26}N_3O_{16}P_2S$ $[M-H]^-$: 622.0514; found: 622.0497.

UDP-6-deoxy-6SSMe-GlcNAc (2)

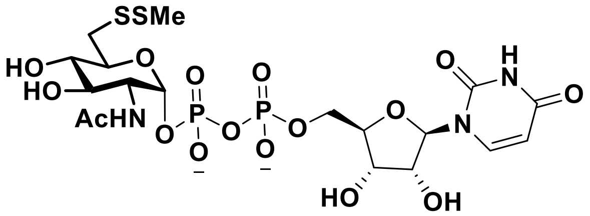

1 (80.0 mg, 0.13 mmol) dissolved in a mixture of MeOH (10 mL) and H_2O (10 mL), and $MeSSO_2Me$ (15 µL, 0.16 mmol) were mixed and this mixture was stirred for 18 h. The solvent was removed in vacuo and the residue purified by flash column chromatography on silica gel (water: methanol: ethyl acetate = 1:2:5) to afford **2** (77.3 mg, 90%) as a white foam. ^{1}H NMR (600 MHz, D_2O) δ 7.97 (d, J = 8.1 Hz, 1H, CO-CH=C$\underline{H}$-N), 5.99 – 5.96 (m, 2H, H-

1[UDP], CO-CH=CH-N), 5.50 (dd, J = 7.6, 3.3 Hz, 1H, H-1[GlcNAc]), 4.40 – 4.35 (m, 2H, H-2[UDP], H-3[UDP]), 4.31 – 4.28 (m, 1H, H-4[UDP]), 4.28 – 4.24 (m, 1H, H-5a[UDP]), 4.22 – 4.18 (m, 1H, H-5b[UDP]), 4.16 (ddd, J = 9.6, 6.4, 2.8 Hz, 1H, H-5 [GlcNAc]), 4.04 – 3.98 (m, 1H, H-2 [GlcNAc]), 3.80 (dd, J = 10.5, 9.1 Hz, 1H, H-3 [GlcNAc]), 3.57 (t, J = 9.5 Hz, 1H, H-4 [GlcNAc]), 3.31 (dd, J = 14.3, 2.8 Hz, 1H, H-6a [GlcNAc]), 3.08 (dd, J = 14.3, 6.4 Hz, 1H, H-6b [GlcNAc]), 2.46 (s, 3H, SCH_3), 2.08 (s, 3H, $COCH_3$). ^{13}C NMR (150 MHz, D_2O) δ 174.73 ($COCH_3$), 166.63 (CO-CH=CH-N), 152.05 (NH-CO-N), 141.60 (CO-CH=CH-N), 102.64 (CO-CH=CH-N), 94.29, 94.25(d, C-1 [GlcNAc]), 88.65 (C-1 [UDP]), 83.11, 83.05 (d, C-4 [UDP]), 73.83 (C-2 [UDP]), 71.95 (C-4 [GlcNAc]), 71.30 (C-5 [GlcNAc]), 70.74 (C-3 [GlcNAc]), 69.49 (C-3 [UDP]), 64.89 (C-5 [UDP]), 53.73, 53.68 (d, C-2 [GlcNAc]), 40.30 (C-6 [GlcNAc]), 22.39 (SCH_3), 22.05 ($COCH_3$). HRMS (ESI-MS): m/z calculated for $C_{18}H_{28}N_3O_{16}P_2S_2$ $[M-H]^-$: 668.0391; found: 668.0349.

Enzymatic Synthesis

Compound 11a

11a was prepared from **10** (3.0 mg, 3.3 µmol) using the general procedure for the installation of β1,6 6-deoxy-6SH-GlcNAc using GCNT1 to full conversion. After purification, **11a** was obtained as a white solid (3.3 mg, 90%).

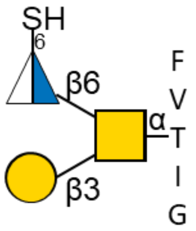

^{1}H (600 MHz, D_2O): δ (ppm)

	H-1	H-2	H-3	H-4	H-5	H-6	NHAc
GalNAc	4.82	4.24	4.00	4.17	4.25	4.00 (dd, J = 11.0, 2.9 Hz, 1H), 3.74	2.09 – 1.97 (m, 6H)
Galactose	4.43 (d, J = 7.8 Hz, 1H)	3.51	3.60 (dd, J = 10.0, 3.3 Hz, 1H)	3.93	n/a	3.77	-
6-deoxy-6SH-GlcNAc	4.55 (d, J = 8.5 Hz, 1H)	3.75	n/a	n/a	3.69	3.33 (d, J = 13.7 Hz, 1H), 2.90 (dd, J = 14.3, 9.1 Hz, 1H)	2.09 – 1.97 (m, 6H)

^{13}C (150 MHz, D_2O): δ (ppm)

	C-1	C-2	C-3	C-4	C-5	C-6	NHAc
GalNAc	99.26	48.58	77.32	69.83	77.29	70.09	22.36
Galactose	104.78	70.73	72.84	68.63	n/a	61.06	-
6-deoxy-6SH-GlcNAc	101.27	55.69	n/a	n/a	74.45	40.47	22.36

HRMS (ESI-MS): m/z calculated for $C_{48}H_{77}N_8O_{20}S$ $[M-H]^-$: 1117.4980; found: 1117.4557.

Compound 12

12 was prepared from **11a** (5.0 mg, 4.5 µmol) using the general procedure for the oxidation of SH to sulfonate using oxone to full conversion. After purification, **12** was obtained as a white solid (4.8 mg, 92%).

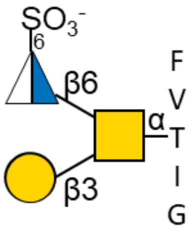

^1H (600 MHz, D_2O): δ (ppm)

	H-1	H-2	H-3	H-4	H-5	H-6	NHAc
GalNAc	4.82	4.23	3.99	n/a	4.25	4.06 (dd, J = 11.2, 2.5 Hz, 1H), 3.77	2.09 – 1.96 (m, 6H)
Galactose	4.39 (d, J = 7.8 Hz, 1H)	3.51	3.60	3.93	n/a	3.80, 3.74	-
6-deoxy-$6SO_3^-$-GlcNAc	4.55 (d, J = 8.5 Hz, 1H)	3.76	n/a	n/a	n/a	3.41, 3.07	2.09 – 1.96 (m, 6H)

^{13}C (150 MHz, D_2O): δ (ppm)

	C-1	C-2	C-3	C-4	C-5	C-6	NHAc
GalNAc	99.30	48.44	77.30	n/a	77.17	70.96	22.41
Galactose	104.90	70.75	n/a	68.84	n/a	61.03	-
6-deoxy-$6SO_3^-$-GlcNAc	101.55	55.69	n/a	n/a	n/a	52.51	22.41

HRMS (ESI-MS): m/z calculated for $C_{48}H_{77}N_8O_{23}S$ $[M-H]^-$: 1165.4827; found: 1165.5056.

Compound 13

13 was prepared from **12** (2.9 mg, 2.5 µmol) using the general procedure for the installation of β1,4 Gal with B4GALT4 to full conversion. After purification, **13** was obtained as a white solid (3.3 mg, 99%).

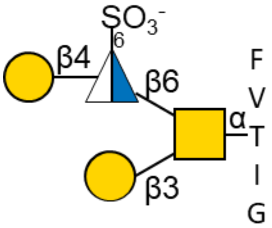

^{1}H (600 MHz, D_2O): δ (ppm)

	H-1	H-2	H-3	H-4	H-5	H-6	NHAc
GalNAc	4.82	4.23	4.00 (dd, J = 11.1, 3.1 Hz, 1H)	4.20	4.25	4.04 (dd, J = 11.3, 2.4 Hz, 1H), 3.79	2.12 – 1.98 (m, 6H)
Galactose-1	4.42 (d, J = 7.8 Hz, 1H)	3.52	3.61	3.92 (d, J = 3.3 Hz, 1H)	n/a	n/a	-
6-deoxy-6SO_3^--GlcNAc	4.57 (d, J = 8.5 Hz, 1H)	3.81	3.67	3.45	3.94	3.58, 3.13 (dd, J = 14.5, 9.9 Hz, 1H)	2.12 – 1.98 (m, 6H)
Galactose-2	4.31 (d, J = 7.8 Hz, 1H)	3.54	3.60	3.89 (d, J = 3.3 Hz, 1H)	n/a	n/a	-

^{13}C (150 MHz, D_2O): δ (ppm)

	C-1	C-2	C-3	C-4	C-5	C-6	NHAc
GalNAc	99.64	48.63	77.49	69.87	77.13	70.78	22.45
Galactose	104.91	70.64	n/a	68.62	n/a	n/a	-
6-deoxy-6SO_3^--GlcNAc	101.66	55.25	72.91	82.26	71.29	51.94	22.45
Galactose-2	103.61	70.89	n/a	68.53	n/a	n/a	-

HRMS (ESI-MS): m/z calculated for $C_{54}H_{87}N_8O_{28}S$ $[M-H]^-$: 1327.5355; found: 1327.5567.

Compound 14

14 was prepared from **13** (1.2 mg, 0.9 µmol) using the general procedure for the installation of α2,3 Neu5Ac with ST3GAL1 then the installation of α2,3 Neu5Ac with ST3GAL4. After purification, **14** was obtained as a white solid (1.5 mg, 85%).

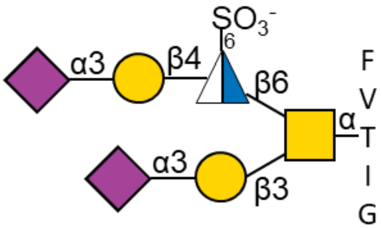

^{1}H (600 MHz, D_2O): δ (ppm)

	H-1	H-2	H-3	H-4	H-5	H-6	H-7	H-8	H-9	NHAc
GalNAc	4.82	4.23	4.02	4.21	4.28	4.03, 3.82	-	-	-	2.11 – 1.99 (m, 12H)
Galactose-1	4.48 (d, J = 7.7 Hz, 1H)	3.52	4.07	3.95	n/a	n/a	-	-	-	-
6-deoxy-6SO_3^--GlcNAc	4.56 (d, J = 9.2 Hz, 1H)	3.81	3.67	3.40	3.94	3.61, 3.09 (dd, J = 14.4, 10.1 Hz, 1H)	-	-	-	2.11 – 1.99 (m, 12H)
Galactose-2	4.31 (d, J = 7.8 Hz, 1H)	3.55	4.02	3.92	n/a	n/a	-	-	-	-
Neu5Ac-1	-	-	2.76, 1.80	3.71	3.86	n/a	n/a	3.90	3.89, 3.65	2.11 – 1.99 (m, 12H)
Neu5Ac-2	-	-	2.76, 1.80	3.71	3.86	n/a	n/a	3.90	3.89, 3.65	2.11 – 1.99 (m, 12H)

^{13}C (150 MHz, D_2O): δ (ppm)

	C-1	C-2	C-3	C-4	C-5	C-6	C-7	C-8	C-9	NHAc
GalNAc	99.41	48.55	77.49	69.43	77.30	70.98	-	-	-	22.28
Galactose	104.75	69.16	75.85	67.49	n/a	n/a	-	-	-	-
6-deoxy-6SO_3^--GlcNAc	101.58	55.30	72.92	82.07	71.35	51.90	-	-	-	22.28
Galactose-2	103.25	69.48	75.71	67.59	n/a	n/a	-	-	-	-
Neu5Ac-1	n/a	n/a	39.89	n/a	51.92	n/a	n/a	71.91	62.76	22.28
Neu5Ac-2	n/a	n/a	39.89	n/a	51.92	n/a	n/a	71.91	62.76	22.28

HRMS (ESI-MS): m/z calculated for $C_{76}H_{120}N_{10}O_{44}S$ $[M-2H]^{2-}$: 954.3596; found: 954.3874.

Compound 15

15 was prepared from **14** (0.9 mg, 0.5 μmol) using the general procedure for the installation of α1,3 Fuc using FUT6 to full conversion. After P6 purification, **15** was obtained as a white solid (0.9 mg, 88%).

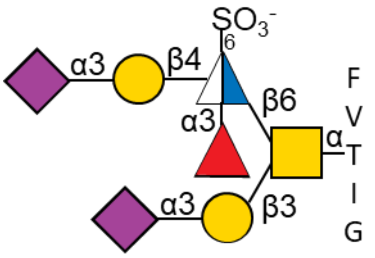

^{1}H (600 MHz, D_2O): δ (ppm)

	H-1	H-2	H-3	H-4	H-5	H-6	H-7	H-8	H-9	NHAc
GalNAc	4.82	4.23	4.02	4.20	4.24	4.08, 3.75	-	-	-	2.09 – 1.99 (m, 12H)
Galactose-1	4.50 (d, J = 7.8 Hz, 1H)	3.52	4.08	3.95	n/a	n/a	-	-	-	-
6-deoxy-6SO_3^--GlcNAc	4.56 (d, J = 8.4 Hz, 1H)	3.99	n/a	n/a	4.02	3.64, 3.18 (dd, J = 14.1, 10.6 Hz, 1H)	-	-	-	2.09 – 1.99 (m, 12H)
Galactose-2	4.45 (d, J = 8.0 Hz, 1H)	3.51	4.07	3.92	n/a	n/a	-	-	-	-
Fucose	5.10 (d, J = 4.1 Hz, 1H)	3.66	3.91	3.80	4.81	1.16 (d, J = 6.5 Hz, 3H)	-	-	-	-
Neu5Ac-1	-	-	2.77 (dd, J = 12.5, 4.7 Hz, 1H), 1.80 (t, J = 12.1 Hz, 1H)	3.70	3.86	n/a	n/a	3.92	3.91, 3.64	2.09 – 1.99 (m, 12H)
Neu5Ac-2	-	-	2.77 (dd, J = 12.5, 4.7 Hz, 1H), 1.80 (t, J = 12.1 Hz, 1H)	3.70	3.86	n/a	n/a	3.92	3.91, 3.64	2.09 – 1.99 (m, 12H)

^{13}C (150 MHz, D_2O): δ (ppm)

	C-1	C-2	C-3	C-4	C-5	C-6	C-7	C-8	C-9	NHAc
GalNAc	99.52	48.35	77.32	70.05	76.78	70.49	-	-	-	22.25
Galactose	104.65	69.15	75.53	67.23	n/a	n/a	-	-	-	-
6-deoxy-6SO_3^--GlcNAc	101.25	55.71	n/a	n/a	71.85	51.61	-	-	-	22.25

Galactose-2	102.60	69.23	75.21	67.15	n/a	n/a	-	-	-	-
Fucose	98.65	n/a	69.25	n/a	66.91	15.24	-	-	-	-
Neu5Ac-1	n/a	n/a	39.89	n/a	51.78	n/a	n/a	72.04	62.63	22.25
Neu5Ac-2	n/a	n/a	39.79	n/a	51.78	n/a	n/a	72.04	62.63	22.25

HRMS (ESI-MS): m/z calculated for $C_{82}H_{130}N_{10}O_{48}S$ $[M-2H]^{2-}$: 1027.3885; found: 1027.4240.

Compound 16

16 was prepared from **14** (0.5 mg, 0.26 µmol) using the general procedure for the 6-O-sulfate installation of internal Galactose with CHST1. When no further product was formed, the reaction was stopped. After purification, **16** was obtained as a white solid (410 µg, 79%).

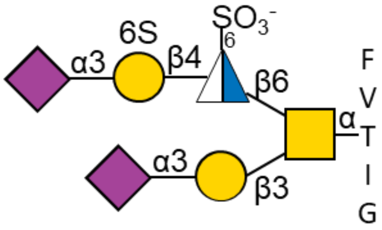

^{1}H (600 MHz, D_2O): δ (ppm)

	H-1	H-2	H-3	H-4	H-5	H-6	H-7	H-8	H-9	NHAc
GalNAc	4.81	4.23	4.02	4.21	4.31	3.98, 3.84	-	-	-	2.11 – 1.95 (m, 12H)
Galactose	4.49 (d, J = 8.0 Hz, 1H)	3.53	4.08	3.97	n/a	n/a	-	-	-	-
6-deoxy-6SO_3^--GlcNAc	4.56	3.81	3.66	3.29	3.93	3.62, 3.07 (dd, J = 14.4, 10.1 Hz, 1H)	-	-	-	2.11 – 1.95 (m, 12H)
Galactose-6S	4.57	3.58	4.14	4.02	3.98	4.18	-	-	-	-
Neu5Ac-1	-	-	2.76, 1.80	3.71	3.86	n/a	n/a	3.90	3.89, 3.65	2.11 – 1.95 (m, 12H)
Neu5Ac-2	-	-	2.76, 1.80	3.71	3.86	n/a	n/a	3.90	3.89, 3.65	2.11 – 1.95 (m, 12H)

^{13}C (150 MHz, D_2O): δ (ppm)

	C-1	C-2	C-3	C-4	C-5	C-6	C-7	C-8	C-9	NHAc
GalNAc	99.27	48.31	77.48	69.32	77.26	70.70	-	-	-	22.17
Galactose	104.66	69.04	75.70	67.41	n/a	n/a	-	-	-	-
6-deoxy-6SO_3^--GlcNAc	101.45	54.91	72.96	82.72	71.75	51.93	-	-	-	22.17

Galactose-6S	103.50	68.66	75.24	67.52	72.72	67.31	-	-	-	-
Neu5Ac-1	n/a	n/a	39.76	n/a	51.83	n/a	n/a	71.87	62.78	22.17
Neu5Ac-2	n/a	n/a	39.76	n/a	51.83	n/a	n/a	71.87	62.78	22.17

HRMS (ESI-MS): m/z calculated for $C_{76}H_{120}N_{10}O_{47}S_2$ $[M-2H]^{2-}$: 994.3380; found: 994.3497.

Compound 19

19 was prepared from reported starting material **17** (6.0 mg, 6.2 μmol) using the general procedure for the installation of β1,3 6-deoxy-6SSMe-GlcNAc using B3GNT2 to get **18b** then the installation of β1,4 Gal using B4GALT1. After purification, **19** was obtained as a white solid (6.7 mg, 78%).

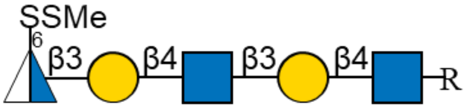

^1H (600 MHz, D_2O): δ (ppm)

	H-1	H-2	H-3	H-4	H-5	H-6	NHAc	SMe
GlcNAc-1	4.51 (d, J = 7.7 Hz, 1H)	3.71	3.68	3.69	3.58	3.97, 3.83	2.07 – 1.97 (m, 9H)	-
Galactose-1	4.46 (d, J = 7.8 Hz, 1H)	3.59	3.73	4.16 (d, J = 3.3 Hz, 1H)	n/a	3.76 (4H)	-	-
GlcNAc-2	4.70 (d, J = 8.4 Hz, 1H)	3.81	3.73	3.74	3.59	3.97, 3.83	2.07 – 1.97 (m, 9H)	-
Galactose-2	4.47 (d, J = 8.0 Hz, 1H)	3.61	3.70	4.27 (d, J = 3.2 Hz, 1H)	n/a	3.76 (4H)	-	-
6-deoxy-6SSMe-GlcNAc	4.68 (d, J = 8.5 Hz, 1H)	3.80	3.59	3.43 (t, J = 9.3 Hz, 1H)	3.69	3.32 (dd, J = 14.3, 2.4 Hz, 1H), 2.88 (dd, J = 14.3, 9.3 Hz, 1H)	2.07 – 1.97 (m, 9H)	2.46 (s, 3H)

^{13}C (150 MHz, D_2O): δ (ppm)

	C-1	C-2	C-3	C-4	C-5	C-6	NHAc	SMe
GlcNAc-1	101.13	55.08	72.55	78.75	74.68	60.07	22.18	-
Galactose-1	103.00	69.77	82.24	68.43	n/a	61.21	-	-
GlcNAc-2	103.21	55.51	72.24	78.37	74.74	60.07	22.18	-
Galactose-2	103.17	69.92	82.24	68.52	n/a	61.21	-	-
6-deoxy-6SSMe-GlcNAc	102.96	55.51	n/a	73.17	n/a	39.12	22.18	22.16

Linker	1	2	3	4	5	6
H	3.87, 3.55	1.58 – 1.52 (m, 2H)	1.35 – 1.25 (m, 2H)	1.49 (p, J = 7.3 Hz, 2H)	3.12 (t, J = 6.7 Hz, 2H)	5.11 (s, 2H)
C	70.54	28.26	22.49	28.49	40.57	66.76

HRMS (ESI-MS): m/z calculated for $C_{50}H_{81}N_4O_{27}S_2$ $[M+H]^+$: 1233.4525; found: 1233.4198.

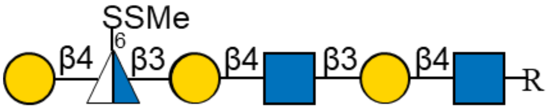

^{1}H (600 MHz, D_2O): δ (ppm)

	H-1	H-2	H-3	H-4	H-5	H-6	NHAc	SMe
GlcNAc-1	4.51	3.72	3.68	3.69	3.58	3.97, 3.83	2.07 – 1.99 (m, 9H)	-
Galactose-1	4.46 (d, J = 7.7 Hz, 1H)	3.59	3.72	4.16 (d, J = 3.3 Hz, 1H)	n/a	3.76 (4H)	-	-
GlcNAc-2	4.70	3.81	3.73	3.74	3.59	3.97, 3.83	2.07 – 1.99 (m, 9H)	-
Galactose-2	4.47 (d, J = 8.0 Hz, 1H)	3.61	3.70	4.27 (d, J = 3.3 Hz, 1H)	n/a	3.77, 3.75	-	-
6-deoxy-6SSMe-GlcNAc	4.70	3.85	n/a	n/a	n/a	3.45 (d, J = 13.9 Hz, 1H), 2.94 (dd, J = 14.1, 9.3 Hz, 1H)	2.07 – 1.99 (m, 9H)	2.45 (s, 3H)
Galactose-3	4.51	3.55	3.68	3.93 (d, J = 3.5 Hz, 1H)	n/a	3.76 (4H)	-	-

^{13}C (150 MHz, D_2O): δ (ppm)

	C-1	C-2	C-3	C-4	C-5	C-6	NHAc	SMe
GlcNAc-1	100.80	55.05	72.55	78.75	74.68	59.96	22.14	-
Galactose-1	102.72	69.77	82.12	68.24	n/a	61.02	-	-
GlcNAc-2	102.80	55.28	72.24	78.37	74.74	59.96	22.14	-
Galactose-2	102.72	69.92	82.12	68.50	n/a	61.02	-	-
6-deoxy-6SSMe-GlcNAc	102.80	55.28	n/a	n/a	n/a	38.64	22.14	22.04
Galactose-3	102.89	71.49	72.58	68.65	n/a	61.02	-	-

Linker	1	2	3	4	5	6
H	3.87, 3.55	1.58 – 1.52 (m, 2H)	1.35 – 1.25 (m, 2H)	1.49 (p, J = 7.3 Hz, 2H)	3.12 (t, J = 6.8 Hz, 2H)	5.11 (s, 2H)
C	70.40	28.36	22.67	28.53	40.44	66.73

HRMS (ESI-MS): m/z calculated for $C_{56}H_{89}N_4O_{32}S_2$ $[M-H]^-$: 1393.4906; found: 1393.4628.

Compound 20

20 was prepared from **19** (2.2 mg, 1.6 μmol) using the general procedure for the installation of α2,3 Neu5Ac using ST3GAL4. After purification, **20** was obtained as a white solid (2.0 mg, 75%).

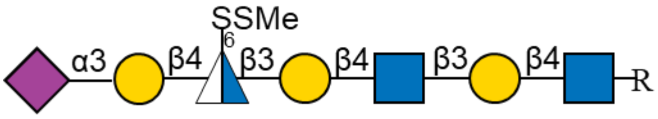

^{1}H (600 MHz, D_2O): δ (ppm)

	H-1	H-2	H-3	H-4	H-5	H-6	H-7	H-8	H-9	NHAc	SMe
GlcNAc-1	4.51 (d, J = 7.6 Hz, 1H)	3.72	3.68	3.69	3.58	3.98, 3.83	-	-	-	2.09 – 1.98 (m, 12H)	-
Galactose-1	4.46	3.58	3.72	4.16 (d, J = 3.3 Hz, 1H)	n/a	3.76 (2H)	-	-	-	-	-
GlcNAc-2	4.70 (d, J = 8.2 Hz, 1H)	3.81	3.73	3.73	3.59	3.96, 3.84	-	-	-	2.09 – 1.98 (m, 12H)	-
Galactose-2	4.48	3.60	3.70	4.27 (d, J = 3.2 Hz, 1H)	n/a	3.77, 3.75	-	-	-	-	-
6-deoxy-6SSMe-GlcNAc	4.69 (d, J = 8.4 Hz, 1H)	3.87	n/a	n/a	3.84	3.50 (d, J = 13.2 Hz, 1H), 2.91 (dd, J = 14.2, 9.4 Hz, 1H)	-	-	-	2.09 – 1.98 (m, 12H)	2.45 (s, 3H)
Galactose-3	4.59 (d, J = 7.9 Hz, 1H)	3.58	4.12 (dd, J = 9.9, 3.2 Hz, 1H)	3.96 (d, J = 3.2 Hz, 1H)	n/a	n/a	-	-	-	-	-
Neu5Ac	-	-	2.77 (dd, J = 12.5, 4.6 Hz, 1H),	3.71	3.87	n/a	n/a	3.90	3.88, 3.66	2.09 – 1.98 (m, 12H)	-

			1.81 (t, J = 12.1 Hz, 1H)								

^{13}C (150 MHz, D_2O): δ (ppm)

	C-1	C-2	C-3	C-4	C-5	C-6	H-7	H-8	H-9	NHAc	SMe
GlcNAc-1	101.21	55.22	72.68	78.81	74.88	60.05	-	-	-	22.23	-
Galactose-1	102.97	69.55	82.29	68.50	n/a	61.10	-	-	-	-	-
GlcNAc-2	103.01	55.34	72.34	78.36	75.11	60.05	-	-	-	22.23	-
Galactose-2	103.21	69.89	82.29	68.63	n/a	61.10	-	-	-	-	-
6-deoxy-6SSMe-GlcNAc	102.81	55.34	n/a	n/a	n/a	38.24	-	-	-	22.23	22.41
Galactose-3	102.93	n/a	75.87	67.75	n/a	n/a	-	-	-	-	-
Neu5Ac	n/a	n/a	39.86	n/a	51.88	n/a	n/a	71.97	62.62	22.23	-

Linker	1	2	3	4	5	6
H	3.87, 3.55	1.58 – 1.52 (m, 2H)	1.35 – 1.25 (m, 2H)	1.49 (p, J = 7.3 Hz, 2H)	3.12 (t, J = 6.8 Hz, 2H)	5.11 (s, 2H)
C	70.40	28.36	22.67	28.53	40.44	66.73

HRMS (ESI-MS): m/z calculated for $C_{67}H_{105}N_5O_{40}S_2$ $[M-2H]^{2-}$: 841.7894; found: 841.8264.

Compound 21

21 was prepared from **20** (2.0 mg, 1.2 μmol) using the general procedure for the installation of α1,3 Fuc using FUT5. After purification, **21** was obtained as a white solid (1.5 mg, 59%).

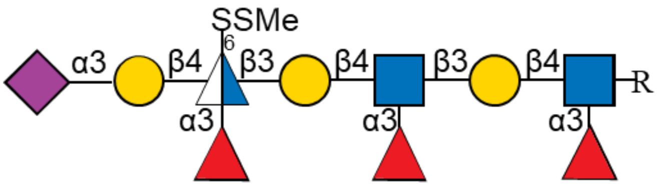

^{1}H (600 MHz, D_2O): δ (ppm)

	H-1	H-2	H-3	H-4	H-5	H-6	H-7	H-8	H-9	NHAc	SMe
GlcNAc-1	4.52 (d, J = 7.9 Hz, 1H)	3.88	n/a	n/a	3.59	3.99, 3.86	-	-	-	2.06 – 1.98 (m, 12H)	-
Galactose-1	4.44 (d, J = 8.0 Hz, 1H)	3.52	3.71	4.11	n/a	3.71 (4H)	-	-	-	-	-
GlcNAc-2	4.71	3.98	n/a	n/a	3.59	3.97, 3.86	-	-	-	2.06 –	-

										1.98 (m, 12H)	
Galactose-2	4.46 (d, J = 7.9 Hz, 1H)	3.53	3.68	4.21 (d, J = 3.3 Hz, 1H)	n/a	3.74 (2H)	-	-	-	-	-
6-deoxy-6SSMe-GlcNAc	4.70	4.02	n/a	n/a	3.87	3.55, 2.94 (dd, J = 13.9, 9.2 Hz, 1H)	-	-	-	2.06 – 1.98 (m, 12H)	2.47 (s, 3H)
Galactose-3	4.58 (d, J = 7.7 Hz, 1H)	3.55	4.11	3.93	n/a	3.71 (4H)	-	-	-	-	-
Fucose-1	5.10 (d, J = 4.0 Hz, 1H)	3.70	n/a	n/a	4.83	1.16 (d, J = 6.6 Hz, 3H)	-	-	-	-	-
Fucose-2	5.12	3.70	n/a	n/a	4.83	1.16 (d, J = 6.6 Hz, 3H)	-	-	-	-	-
Fucose-3	5.14	3.70	n/a	n/a	4.83	1.18 (d, J = 6.6 Hz, 3H)	-	-	-	-	-
Neu5Ac	-	-	2.79 (dd, J = 12.6, 4.7 Hz, 1H), 1.80 (t, J = 12.1 Hz, 1H)	3.70	3.87	n/a	n/a	3.90	3.90, 3.66	2.06 – 1.98 (m, 12H)	-

^{13}C (150 MHz, D_2O): δ (ppm)

	C-1	C-2	C-3	C-4	C-5	C-6	H-7	H-8	H-9	NHAc	SMe
GlcNAc-1	101.10	56.23	n/a	n/a	75.16	60.01	-	-	-	22.25	-
Galactose-1	101.94	70.42	n/a	68.40	n/a	61.78	-	-	-	-	-
GlcNAc-2	102.86	56.10	n/a	n/a	75.02	60.01	-	-	-	22.25	-
Galactose-2	101.94	69.87	n/a	68.23	n/a	61.53	-	-	-	-	-
6-deoxy-6SSMe-GlcNAc	102.65	56.08	n/a	n/a	n/a	38.66	-	-	-	22.25	22.41

Galactose-3	102.51	69.37	75.90	67.34	n/a	61.78	-	-	-	-	-
Fucose-1	98.69	n/a	n/a	n/a	66.82	15.50	-	-	-	-	-
Fucose-2	98.57	n/a	n/a	n/a	66.82	15.50	-	-	-	-	-
Fucose-3	98.67	n/a	n/a	n/a	66.82	15.50	-	-	-	-	-
Neu5Ac	n/a	n/a	39.80	n/a	51.78	n/a	n/a	72.15	62.68	22.25	-

Linker	1	2	3	4	5	6
H	3.88, 3.57	1.58 – 1.52 (m, 2H)	1.35 – 1.27 (m, 2H)	1.49 (p, J = 7.3 Hz, 2H)	3.12 (t, J = 6.8 Hz, 2H)	5.12 (s, 2H)
C	70.67	28.41	22.56	28.53	40.41	66.88

HRMS (ESI-MS): m/z calculated for $C_{85}H_{135}N_5O_{52}S_2$ $[M-2H]^{2-}$: 1060.8763; found: 1060.8639.

Compound 22

22 was prepared from **21** (1.5 mg, 0.7 μmol) using the general procedure for the conversion of SSMe to sulfonate. After purification, **22** was obtained as a white solid (1.2 mg, 81%).

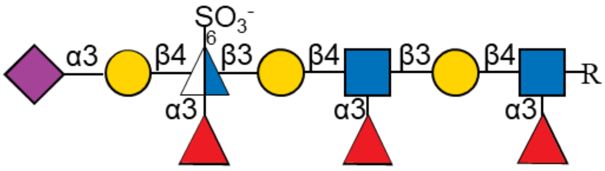

^1H (600 MHz, D_2O): δ (ppm)

	H-1	H-2	H-3	H-4	H-5	H-6	H-7	H-8	H-9	NHAc
GlcNAc-1	4.52	3.88	n/a	n/a	3.58	3.99, 3.84	-	-	-	2.07 – 1.98 (m, 12H)
Galactose-1	4.44 (d, J = 8.1 Hz, 1H)	3.51	3.70	4.11	n/a	3.71 (6H)	-	-	-	-
GlcNAc-2	4.71	3.98	n/a	n/a	3.58	3.97, 3.85	-	-	-	2.07 – 1.98 (m, 12H)
Galactose-2	4.47 (d, J = 7.4 Hz, 1H)	3.50	3.69	4.20	n/a	3.71 (6H)	-	-	-	-
6-deoxy-$6SO_3^-$-GlcNAc	4.70	4.02	n/a	n/a	4.00	3.65, 3.24 (t, J = 12.1 Hz, 1H)	-	-	-	2.07 – 1.98 (m, 12H)
Galactose-3	4.55 (d, J = 7.6 Hz,	3.53	4.12	3.93	n/a	3.71 (6H)	-	-	-	-

	1H)									
Fucose-1	5.10	3.70	n/a	n/a	4.82	1.16 (3H)	-	-	-	-
Fucose-2	5.12	3.70	n/a	n/a	4.82	1.16 (3H)	-	-	-	-
Fucose-3	5.13	3.70	n/a	n/a	4.82	1.18 (3H)	-	-	-	-
Neu5Ac	-	-	2.77, 1.80 (t, J = 12.0 Hz, 1H)	3.70	3.86	n/a	n/a	3.90	3.89, 3.65	2.07 – 1.98 (m, 12H)

^{13}C (150 MHz, D_2O): δ (ppm)

	C-1	C-2	C-3	C-4	C-5	C-6	H-7	H-8	H-9	NHAc
GlcNAc-1	101.18	55.73	n/a	n/a	75.30	59.94	-	-	-	22.32
Galactose-1	101.82	70.60	82.08	68.56	n/a	61.86	-	-	-	-
GlcNAc-2	102.51	55.99	n/a	n/a	75.30	59.94	-	-	-	22.32
Galactose-2	101.58	70.60	82.08	67.74	n/a	61.86	-	-	-	-
6-deoxy-6SO_3^--GlcNAc	102.43	55.88	n/a	n/a	72.18	51.60	-	-	-	22.32
Galactose-3	102.14	69.77	75.54	67.44	n/a	61.86	-	-	-	-
Fucose-1	98.72	n/a	n/a	n/a	66.82	15.50	-	-	-	-
Fucose-2	98.72	n/a	n/a	n/a	66.82	15.50	-	-	-	-
Fucose-3	98.72	n/a	n/a	n/a	66.82	15.50	-	-	-	-
Neu5Ac	n/a	n/a	39.80	n/a	51.78	n/a	n/a	72.34	62.96	22.32

Linker	1	2	3	4	5	6
H	3.87, 3.57	1.58 – 1.52 (m, 2H)	1.35 – 1.27 (m, 2H)	1.52 – 1.44 (m, 2H)	3.19 – 3.07 (m, 2H)	5.12 (s, 2H)
C	70.67	28.41	22.41	28.61	40.52	66.97

HRMS (ESI-MS): m/z calculated for $C_{84}H_{133}N_5O_{55}S$ $[M-2H]^{2-}$: 1061.8748; found: 1061.8271.

Compound 23

23 was prepared from **22** (0.5 mg, 0.23 μmol) using the general procedure for the Cbz deprotection from the linker with $Pd(OH)_2$ reduction. After purification, **23** was obtained as a white solid (310 μg, 67%).

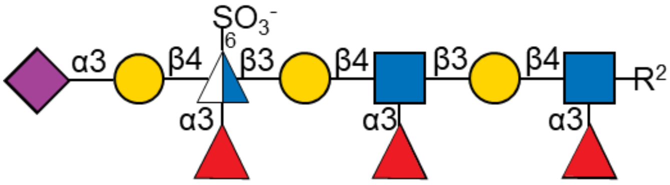

^{1}H (600 MHz, D_2O): δ (ppm)

	H-1	H-2	H-3	H-4	H-5	H-6	H-7	H-8	H-9	NHAc
GlcNAc-1	4.53	3.89	n/a	n/a	3.60	3.99, 3.84	-	-	-	2.05 – 1.99 (m, 12H)
Galactose-1	4.44	3.51	3.70	4.11	n/a	3.71 (6H)	-	-	-	-
GlcNAc-2	4.71	3.98	n/a	n/a	3.60	3.97, 3.85	-	-	-	2.05 – 1.99 (m, 12H)
Galactose-2	4.47	3.50	3.69	4.20	n/a	3.71 (6H)	-	-	-	-
6-deoxy-6SO_3^--GlcNAc	4.70	4.02	n/a	n/a	4.00	3.65, 3.24	-	-	-	2.05 – 1.99 (m, 12H)
Galactose-3	4.55	3.54	4.11	3.94	n/a	3.71 (6H)	-	-	-	-
Fucose-1	5.10	3.70	n/a	n/a	4.82	1.20 – 1.13 (m, 9H)	-	-	-	-
Fucose-2	5.12	3.70	n/a	n/a	4.82	1.20 – 1.13 (m, 9H)	-	-	-	-
Fucose-3	5.13	3.70	n/a	n/a	4.82	1.20 – 1.13 (m, 9H)	-	-	-	-
Neu5Ac	-	-	2.77, 1.80 (t, J = 12.1 Hz, 1H)	3.70	3.86	n/a	n/a	3.90	3.89, 3.64	2.05 – 1.99 (m, 12H)

^{13}C (150 MHz, D_2O): δ (ppm)

	C-1	C-2	C-3	C-4	C-5	C-6	H-7	H-8	H-9	NHAc
GlcNAc-1	101.22	55.89	n/a	n/a	75.11	59.92	-	-	-	22.31
Galactose-1	102.06	70.59	82.05	68.38	n/a	61.72	-	-	-	-
GlcNAc-2	102.61	n/a	n/a	n/a	75.11	59.92	-	-	-	22.31
Galactose-2	101.79	70.59	82.05	67.90	n/a	61.72	-	-	-	-
6-deoxy-6SO_3^--GlcNAc	102.27	56.21	n/a	n/a	n/a	n/a	-	-	-	22.31
Galactose-3	102.30	69.47	75.68	67.48	n/a	61.72	-	-	-	-
Fucose-1	98.72	n/a	n/a	n/a	66.82	15.39	-	-	-	-
Fucose-2	98.72	n/a	n/a	n/a	66.82	15.39	-	-	-	-
Fucose-3	98.72	n/a	n/a	n/a	66.82	15.39	-	-	-	-
Neu5Ac	n/a	n/a	n/a	n/a	51.87	n/a	n/a	72.55	62.73	22.31

Linker	1	2	3	4	5
H	3.90, 3.60	1.64 – 1.55 (m, 2H)	1.46 – 1.37 (m, 2H)	1.73 – 1.64 (m, 2H)	3.00 (t, J = 7.8 Hz, 2H)
C	70.60	28.21	22.16	26.62	39.35

HRMS (ESI-MS): m/z calculated for $C_{76}H_{127}N_5O_{53}S$ $[M-2H]^{2-}$: 994.8564; found: 994.8128.

Compound 24-26 preparation

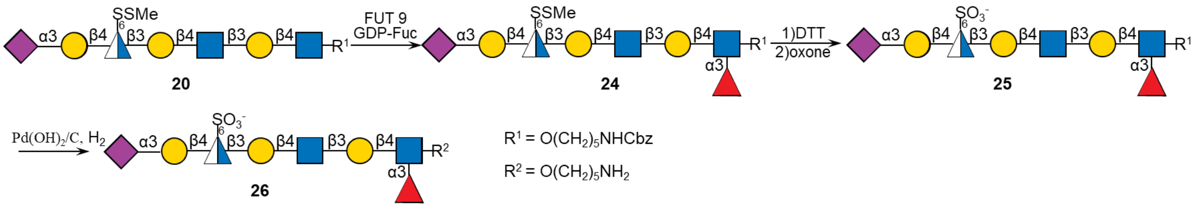

Compound 24

24 was prepared from **20** (3.5 mg, 2.1 μmol) using the general procedure for the installation of α1,3 Fuc using FUT9. After purification, **24** was obtained as a white solid (2.5 mg, 65%).

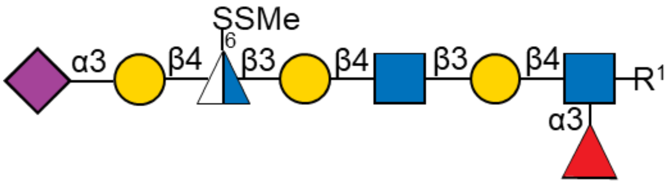

^{1}H (600 MHz, D_2O): δ (ppm)

	H-1	H-2	H-3	H-4	H-5	H-6	H-7	H-8	H-9	NHAc	SMe
GlcNAc-1	4.52 (d, J = 7.9 Hz, 1H)	3.87	n/a	n/a	3.58	3.97, 3.83	-	-	-	2.05 – 1.98 (m, 12H)	-
Galactose-1	4.44 (d, J = 7.8 Hz, 1H)	3.52	3.72	4.10 (d, J = 3.4 Hz, 1H)	n/a	3.71 (2H)	-	-	-	-	-
GlcNAc-2	4.70	3.81	3.73	3.73	3.59	3.97, 3.83	-	-	-	2.05 – 1.98 (m, 12H)	-
Galactose-2	4.48 (d, J = 7.9 Hz, 1H)	3.60	3.70	4.27 (d, J = 3.3 Hz, 1H)	n/a	3.75 (4H)	-	-	-	-	-
6-deoxy-6SSMe-GlcNAc	4.69	3.87	n/a	n/a	3.83	3.51, 2.91 (dd, J = 14.2, 9.2 Hz,	-	-	-	2.05 – 1.98 (m, 12H)	2.45 (s, 3H)

						1H)					
Galactose-3	4.59 (d, J = 7.9 Hz, 1H)	3.58	4.12 (dd, J = 9.8, 3.1 Hz, 1H)	3.95 (d, J = 3.2 Hz, 1H)	n/a	3.75 (4H)	-	-	-	-	-
Fucose	5.09 (d, J = 4.0 Hz, 1H)	3.70	n/a	n/a	4.82	1.16 (d, J = 6.6 Hz, 3H)	-	-	-	-	-
Neu5Ac	-	-	2.77 (dd, J = 12.5, 4.7 Hz, 1H), 1.80 (t, J = 12.2 Hz, 1H)	3.70	3.85	n/a	n/a	3.89	3.88, 3.65	2.05 – 1.98 (m, 12H)	-

^{13}C (150 MHz, D_2O): δ (ppm)

	C-1	C-2	C-3	C-4	C-5	C-6	H-7	H-8	H-9	NHAc	SMe
GlcNAc-1	101.08	55.06	n/a	n/a	75.11	60.01	-	-	-	22.24	-
Galactose-1	101.92	70.65	81.91	68.50	n/a	61.40	-	-	-	-	-
GlcNAc-2	103.06	55.24	72.26	78.33	74.75	60.01	-	-	-	22.24	-
Galactose-2	102.83	69.97	81.91	68.46	n/a	61.31	-	-	-	-	-
6-deoxy-6SSMe-GlcNAc	102.79	55.44	n/a	n/a	73.00	n/a	-	-	-	22.24	22.04
Galactose-3	102.99	69.40	75.91	67.92	n/a	61.31	-	-	-	-	-
Fucose	98.90	n/a	n/a	n/a	66.89	15.38	-	-	-	-	-
Neu5Ac	n/a	n/a	39.78	n/a	51.88	n/a	n/a	72.10	62.63	22.24	-

Linker	1	2	3	4	5	6
H	3.87, 3.55	1.58 – 1.52 (m, 2H)	1.35 – 1.25 (m, 2H)	1.49 (p, J = 7.3 Hz, 2H)	3.12 (t, J = 6.8 Hz, 2H)	5.11 (s, 2H)
C	70.40	28.36	22.67	28.53	40.44	66.73

HRMS (ESI-MS): m/z calculated for $C_{73}H_{115}N_5O_{44}S_2$ $[M-2H]^{2-}$: 914.8183; found: 914.8595.

Compound 25

25 was prepared from **24** (2.5 mg, 1.3 μmol) using the general procedure for the conversion of SSMe to sulfonate. After purification, **25** was obtained as a white solid (1.1 mg, 44%).

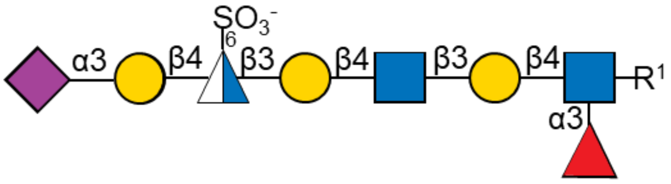

^{1}H (600 MHz, D_2O): δ (ppm)

	H-1	H-2	H-3	H-4	H-5	H-6	H-7	H-8	H-9	NHAc
GlcNAc-1	4.51	3.87	n/a	n/a	3.58	3.97, 3.83	-	-	-	2.07 – 1.93 (m, 12H)
Galactose-1	4.44 (d, J = 7.9 Hz, 1H)	3.51	3.70	4.10 (d, J = 3.2 Hz, 1H)	n/a	3.71 (2H)	-	-	-	-
GlcNAc-2	4.70	3.81	3.73	3.73	3.59	3.97, 3.83	-	-	-	2.07 – 1.93 (m, 12H)
Galactose-2	4.48 (d, J = 7.8 Hz, 1H)	3.58	3.70	4.28	n/a	3.75 (4H)	-	-	-	-
6-deoxy-6SO_3^--GlcNAc	4.69	3.85	n/a	n/a	3.96	3.63(1H), 3.17 (dd, J = 14.6, 10.1 Hz, 1H)	-	-	-	2.07 – 1.93 (m, 12H)
Galactose-3	4.57 (d, J = 7.7 Hz, 1H)	3.58	4.12 (dd, J = 9.8, 3.0 Hz, 1H)	3.97	n/a	3.75 (4H)	-	-	-	-
Fucose	5.09 (d, J = 3.9 Hz, 1H)	3.70	n/a	n/a	4.81	1.15 (d, J = 6.5 Hz, 3H)	-	-	-	-
Neu5Ac	-	-	2.75 (dd, J = 12.5, 4.7 Hz, 1H), 1.80 (t, J = 12.2 Hz, 1H)	3.70	3.85	n/a	n/a	3.90	3.89, 3.63	2.07 – 1.93 (m, 12H)

^{13}C (150 MHz, D_2O): δ (ppm)

	C-1	C-2	C-3	C-4	C-5	C-6	H-7	H-8	H-9	NHAc
GlcNAc-1	101.07	55.34	n/a	n/a	75.13	59.95	-	-	-	22.19
Galactose-1	101.77	70.85	82.36	68.45	n/a	61.36	-	-	-	-
GlcNAc-2	102.58	55.25	72.32	78.52	75.02	59.95	-	-	-	22.19
Galactose-2	103.09	69.73	82.36	67.66	n/a	61.36	-	-	-	-
6-deoxy-	103.01	55.31	n/a	n/a	71.29	51.60	-	-	-	22.19

$6SO_3^-$-GlcNAc										
Galactose-3	103.17	69.73	75.63	67.66	n/a	61.36	-	-	-	-
Fucose	98.72	n/a	n/a	n/a	66.85	15.39	-	-	-	-
Neu5Ac	n/a	n/a	39.79	n/a	51.83	n/a	n/a	72.10	62.72	22.19

Linker	1	2	3	4	5	6
H	3.87, 3.55	1.58 – 1.52 (m, 2H)	1.35 – 1.25 (m, 2H)	1.48 (p, J = 7.3 Hz, 2H)	3.14 – 3.08 (m, 2H)	5.11 (s, 2H)
C	70.40	28.36	22.67	28.53	40.44	66.73

HRMS (ESI-MS): m/z calculated for $C_{72}H_{113}N_5O_{47}S$ $[M-2H]^{2-}$: 915.8169; found: 915.8670.

Compound 26

26 was prepared from **25** (0.5 mg, 0.23 µmol) using the general procedure Cbz deprotection from the linker with $Pd(OH)_2$ reduction. After purification, **26** was obtained as a white solid (197 µg, 50%).

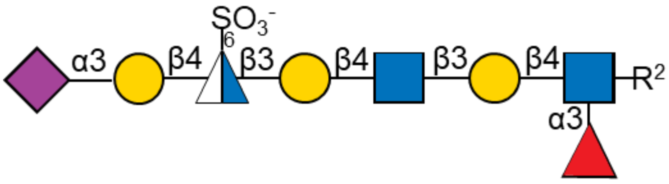

^1H (600 MHz, D_2O): δ (ppm)

	H-1	H-2	H-3	H-4	H-5	H-6	H-7	H-8	H-9	NHAc
GlcNAc-1	4.54 (d, J = 8.1 Hz, 1H)	3.90	n/a	n/a	3.59	3.97, 3.85	-	-	-	2.07 – 2.00 (m, 12H)
Galactose-1	4.45 (d, J = 7.9 Hz, 1H)	3.52	3.73	4.11 (d, J = 3.3 Hz, 1H)	n/a	3.71 (2H)	-	-	-	-
GlcNAc-2	4.71	3.80	3.73	3.74	3.59	3.97, 3.85	-	-	-	2.07 – 2.00 (m, 12H)
Galactose-2	4.49 (d, J = 7.9 Hz, 1H)	3.58	3.73	4.29 (d, J = 3.2 Hz, 1H)	n/a	3.74 (4H)	-	-	-	-
6-deoxy-$6SO_3^-$-GlcNAc	4.69	3.86	n/a	n/a	3.99	3.67(1H), 3.18 (dd, J = 14.6, 10.0 Hz, 1H)	-	-	-	2.07 – 2.00 (m, 12H)
Galactose-3	4.58 (d, J = 7.9 Hz,	3.60	4.13 (dd, J = 10.0,	3.98	n/a	3.74 (4H)	-	-	-	-

	1H)		3.0 Hz, 1H)							
Fucose	5.11 (d, J = 4.0 Hz, 1H)	3.70	n/a	n/a	4.82	1.16 (d, J = 6.5 Hz, 1H)	-	-	-	-
Neu5Ac	-	-	2.76 (dd, J = 12.4, 4.6 Hz, 1H), 1.81 (t, J = 12.1 Hz, 1H)	3.71	3.86	n/a	n/a	3.90	3.89, 3.65	2.07 – 2.00 (m, 12H)

^{13}C (150 MHz, D_2O): δ (ppm)

	C-1	C-2	C-3	C-4	C-5	C-6	H-7	H-8	H-9	NHAc
GlcNAc-1	101.16	56.01	n/a	n/a	74.94	59.93	-	-	-	22.19
Galactose-1	101.99	70.62	82.11	68.42	n/a	61.55	-	-	-	-
GlcNAc-2	103.28	55.24	72.24	78.52	74.94	59.93	-	-	-	22.19
Galactose-2	102.99	69.45	82.11	67.75	n/a	61.20	-	-	-	-
6-deoxy-6SO_3^--GlcNAc	102.22	55.10	n/a	n/a	71.48	51.76	-	-	-	22.19
Galactose-3	103.22	69.18	75.55	67.63	n/a	61.20	-	-	-	-
Fucose	98.82	n/a	n/a	n/a	66.81	15.48	-	-	-	-
Neu5Ac	n/a	n/a	39.68	n/a	51.93	n/a	n/a	71.88	62.72	22.19

Linker	1	2	3	4	5
H	3.91, 3.61	1.60 (p, J = 6.5 Hz, 2H)	1.45 – 1.36 (m, 1H)	1.68 (p, J = 7.7 Hz, 2H)	3.01 – 2.96 (m, 2H)
C	70.37	28.24	22.49	26.61	39.44

HRMS (ESI-MS): m/z calculated for $C_{64}H_{107}N_5O_{45}S$ $[M-2H]^{2-}$: 848.7985; found: 848.8177.

3) Microarray Procedure

Protein Design and Expression

The pA-LS, containing domain B of protein A (pA) of Staphylococcus aureus (amino acid 212-270, UniProt accession number P38507) and 6,7-dimethyl-8-ribityllumazine synthase (LS) of Aquifex aeolicus (GenBank accession number WP_010880027.1), was constructed in a pUC57 plasmid by GenScript USA, Inc. Besides pA and LS, the pA-LS sequence contained a N-terminal Gly-Ser linker and a streptavidin tag II (WSHPQFEK).[11] The pA-LS was ligated into an expression vector, containing a CD5 signal sequence. pCDNA5-Siglec plasmids were kindly provided by Matthew Macauley, University of Alberta.[12]

Recombinant trimeric IAV hemagglutinin ectodomain proteins (HA) were cloned into the pCD5 expression vector (an example is addgene plasmid #182546)[13] in frame with a GCN4 trimerization motif (KQIEDKIEEIESKQKKIENEIARIKK), a superfolder GFP[14] and the Twin-Strep-tag (WSHPQFEKGGGSGGGSWSHPQFEK); IBA, Germany). Mutations in HAs were generated by site-directed mutagenesis.

The proteins were expressed by poly-ethylenimine I (PEI)-transfecting 40-60% confluent HEK293S GnTI(-) cells. Before addition to the cells, the DNA/PEI mix was incubated on Dulbecco's Modified Eagle Medium (DMEM) for 20 min and 1/3 of the medium was removed from the cell dishes. At 6 h post-transfection, the medium was replaced with 293 SFM II medium (Gibco) supplemented with Primatone (3.0 g/L), bicarbonate (3.6 g/L), glucose (2.0 g/L), valproic acid (0.4 g/L), glutaMAX (1%), and DMSO (1.5%). Cells were incubated for 5 days at 37 °C and 5% CO_2 before supernatants were collected. Proteins containing superfolder GFP were quantified by measuring fluorescence (excitation 480 nm; emission 520 nm) with the POLARstar Omega (BMG Labtech). Protein expression was checked by western blotting using a StrepMAB-Classic HRP antibody (IBA Lifesciences). All proteins were purified using Strep-Tactin Sepharose beads (IBA Lifesciences) and subsequently analyzed on SDS-PAGE gels, which were stained with Coomassie blue.

Glycan Microarray Binding Studies

Siglecs, and HAs, both at 50 µg/ml, were either premixed with pA-LS or pre-complexed with human anti-streptag and goat anti-human-Alexa555 (#A21433, Thermo Fisher Scientific) antibodies in a 4:2:1 molar ratio respectively in 50 µL PBS with 0.1% Tween-20. Biotinylated lectins (5 µg/mL) were pre-complexed with streptavidin-Alexa555 (#S32355, Thermo Fisher Scientific) in a 5:1 weight ratio. The following biotinylated lectins from Vector Laboratories were used: MAL-I (B-1315-2), MAL-II (B-1265-1, and SNA (B-1305-2). The mixtures were incubated on ice for 15 min and afterward incubated on the surface of the array for 90 min in a humidified chamber. The siglec-pA-LS complexes where subsequently detected with human anti-streptag (10 µg/mL) and thereafter with goat-anti-human-alexa647 (5 µg/mL) with washes in between as the final was as described next. Slides were rinsed successively with PBS-T (0.1% Tween-20), PBS, and deionized water. After washing successively with PBS-T (0.1% Tween-20), PBS, and deionized water, a mixture of 10 µg/mL goat anti-mouse IgM-HRP (#1021-05, Southern Biotech) and 5 µg/mL donkey anti-goat IgG-Alexa555 (#A21432, Thermo Fisher Scientific) in 40 µL PBS with 0.1% Tween-20 was incubated on the slide for 90 min in a humidified chamber. Afterward, the slides were

rinsed successively with PBS-T (0.1% Tween-20), PBS, and deionized water. The arrays were dried by centrifugation and immediately scanned as described previously.[4] Processing of the six replicates was performed by removing the highest and lowest replicate and subsequently calculating the mean value and standard deviation over the four remaining replicates.

4) Molecular Dynamic Simulations of the Binding of Siglec-4 to Sulfate and Sulfonate Modified Sialyl LacNAc

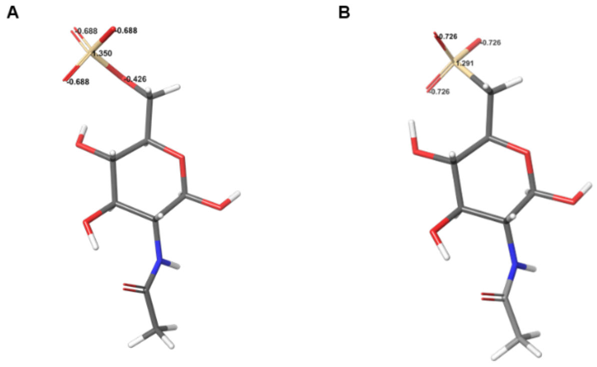

Figure S4. Energy minimized structures of the sulfated GlcNAc moiety of the Neu5Acα(2→3)Galβ(1→4)GlcNAc(6-SO_3^-)β-OH trisaccharide (A) and of the sulfonated isostere GlcNAc of the Neu5Acα(2→3)Galβ(1→4)GlcNAc(6-deoxy-SO_3^-)β-OH, as derived from molecular minimization and DFT calculations.

5) References

(1) Peng, W. J.; Pranskevich, J.; Nycholat, C.; Gilbert, M.; Wakarchuk, W.; Paulson, J. C.; Razi, N. *Helicobacter pylori* beta 1,3-*N*-acetylglucosaminyltransferase for versatile synthesis of type 1 and type 2 poly-LacNAcs on *N*-linked, *O*-linked and I-antigen glycans. *Glycobiology* **2012**, *22*, 1453-1464.

(2) Li, Y.; Xue, M.; Sheng, X.; Yu, H.; Zeng, J.; Thon, V.; Chen, Y.; Muthana, M. M.; Wang, P. G.; Chen, X. Donor substrate promiscuity of bacterial β1–3-N-acetylglucosaminyltransferases and acceptor substrate flexibility of β1–4-galactosyltransferases. *Bioorg. Med. Chem.* **2016**, *24*, 1696-1705.

(3) Prudden, A. R.; Liu, L.; Capicciotti, C. J.; Wolfert, M. A.; Wang, S.; Gao, Z.; Meng, L.; Moremen, K. W.; Boons, G. J. Synthesis of asymmetrical multiantennary human milk oligosaccharides. *Proc. Natl. Acad. Sci. U. S. A.* **2017**, *114*, 6954-6959.

(4) Wu, Y.; Vos, G. M.; Huang, C.; Chapla, D.; Kimpel, A. L. M.; Moremen, K. W.; de Vries, R. P.; Boons, G.-J. Exploiting substrate specificities of 6-O-sulfotransferases to enzymatically synthesize keratan sulfate oligosaccharides. *JACS Au* **2023**, *3*, 3155-3164.

(5) Wu, Y.; Bosman, G. P.; Chapla, D.; Huang, C.; Moremen, K. W.; de Vries, R. P.; Boons, G.-J. A biomimetic synthetic strategy can provide keratan sulfate I and II oligosaccharides with diverse fucosylation and sulfation patterns. *J. Am Chem. Soc.* **2024**, *146*, 9230-9240.

(6) Wang, W.; Hu, T.; Frantom, P. A.; Zheng, T.; Gerwe, B.; Del Amo, D. S.; Garret, S.; Seidel, R. D., III; Wu, P. Chemoenzymatic synthesis of GDP-L-fucose and the Lewis X glycan derivatives. *Proc. Natl. Acad. Sci. U. S. A.* **2009**, *106*, 16096-16101.

(7) Meng, L.; Forouhar, F.; Thieker, D.; Gao, Z.; Ramiah, A.; Moniz, H.; Xiang, Y.; Seetharaman, J.; Milaninia, S.; Su, M.; Bridger, R.; Veillon, L.; Azadi, P.; Kornhaber, G.; Wells, L.; Montelione, G. T.; Woods, R. J.; Tong, L.; Moremen, K. W. Enzymatic basis for *N*-glycan sialylation: structure of rat alpha2,6-sialyltransferase (ST6GAL1) reveals conserved and unique features for glycan sialylation. *J. Biol. Chem.* **2013**, *288*, 34680-34698.

(8) Moremen, K. W.; Ramiah, A.; Stuart, M.; Steel, J.; Meng, L.; Forouhar, F.; Moniz, H. A.; Gahlay, G.; Gao, Z.; Chapla, D.; Wang, S.; Yang, J. Y.; Prabhakar, P. K.; Johnson, R.; Rosa, M. D.; Geisler, C.; Nairn, A. V.; Seetharaman, J.; Wu, S. C.; Tong, L.; Gilbert, H. J.; LaBaer, J.; Jarvis, D. L. Expression system for structural and functional studies of human glycosylation enzymes. *Nat. Chem. Biol.* **2018**, *14*, 156-162.

(9) Liu, F.; Vijayakrishnan, B.; Faridmoayer, A.; Taylor, T. A.; Parsons, T. B.; Bernardes, G. J.; Kowarik, M.; Davis, B. G. Rationally designed short polyisoprenol-linked PglB

substrates for engineered polypeptide and protein N-glycosylation. *J. Am. Chem. Soc.* **2014**, *136*, 566-569.

(10) Chuh, K. N.; Zaro, B. W.; Piller, F.; Piller, V.; Pratt, M. R. Changes in metabolic chemical reporter structure yield a selective probe of O-GlcNAc modification. *J. Am. Chem. Soc.* **2014**, *136*, 12283-12295.

(11) Li, W.; Hulswit, R. J. G.; Widjaja, I.; Raj, V. S.; McBride, R.; Peng, W.; Widagdo, W.; Tortorici, M. A.; van Dieren, B.; Lang, Y.; van Lent, J. W. M.; Paulson, J. C.; de Haan, C. A. M.; de Groot, R. J.; van Kuppeveld, F. J. M.; Haagmans, B. L.; Bosch, B. J. Identification of sialic acid-binding function for the Middle East respiratory syndrome coronavirus spike glycoprotein. *Proc. Natl. Acad. Sci. U. S. A.* **2017**, *114*, E8508-E8517.

(12) Rodrigues, E.; Jung, J.; Park, H.; Loo, C.; Soukhtehzari, S.; Kitova, E. N.; Mozaneh, F.; Daskhan, G.; Schmidt, E. N.; Aghanya, V.; Sarkar, S.; Streith, L.; St Laurent, C. D.; Nguyen, L.; Julien, J. P.; West, L. J.; Williams, K. C.; Klassen, J. S.; Macauley, M. S. A versatile soluble siglec scaffold for sensitive and quantitative detection of glycan ligands. *Nat. Commun.* **2020**, *11*, 5091.

(13) Broszeit, F.; Tzarum, N.; Zhu, X.; Nemanichvili, N.; Eggink, D.; Leenders, T.; Li, Z.; Liu, L.; Wolfert, M. A.; Papanikolaou, A.; Martinez-Romero, C.; Gagarinov, I. A.; Yu, W.; Garcia-Sastre, A.; Wennekes, T.; Okamatsu, M.; Verheije, M. H.; Wilson, I. A.; Boons, G. J.; de Vries, R. P. N-Glycolylneuraminic acid as a receptor for influenza A viruses. *Cell Rep.* **2019**, *27*, 3284-3294 e6.

(14) Nemanichvili, N.; Tomris, I.; Turner, H. L.; McBride, R.; Grant, O. C.; van der Woude, R.; Aldosari, M. H.; Pieters, R. J.; Woods, R. J.; Paulson, J. C.; Boons, G. J.; Ward, A. B.; Verheije, M. H.; de Vries, R. P. Fluorescent trimeric hemagglutinins reveal multivalent receptor binding properties. *J. Mol. Biol.* **2019**, *431*, 842-856.

6) NMR Spectra

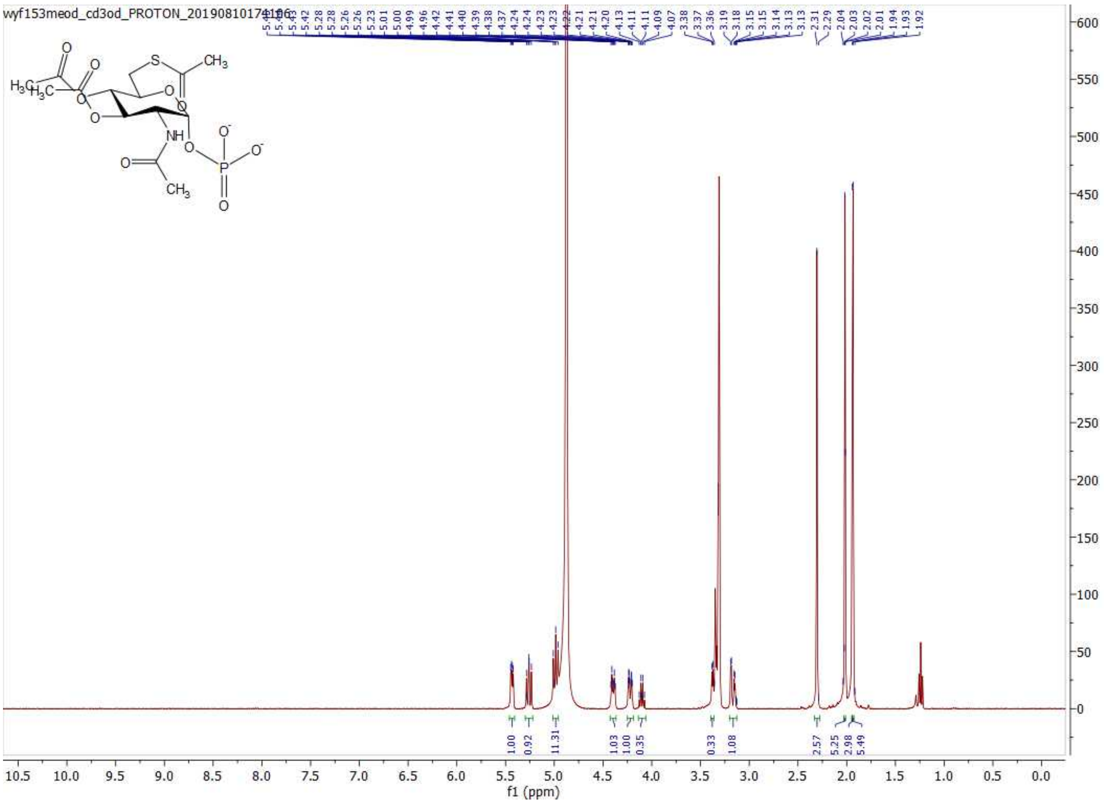

^1H NMR of 8; 600 MHz; MeOD

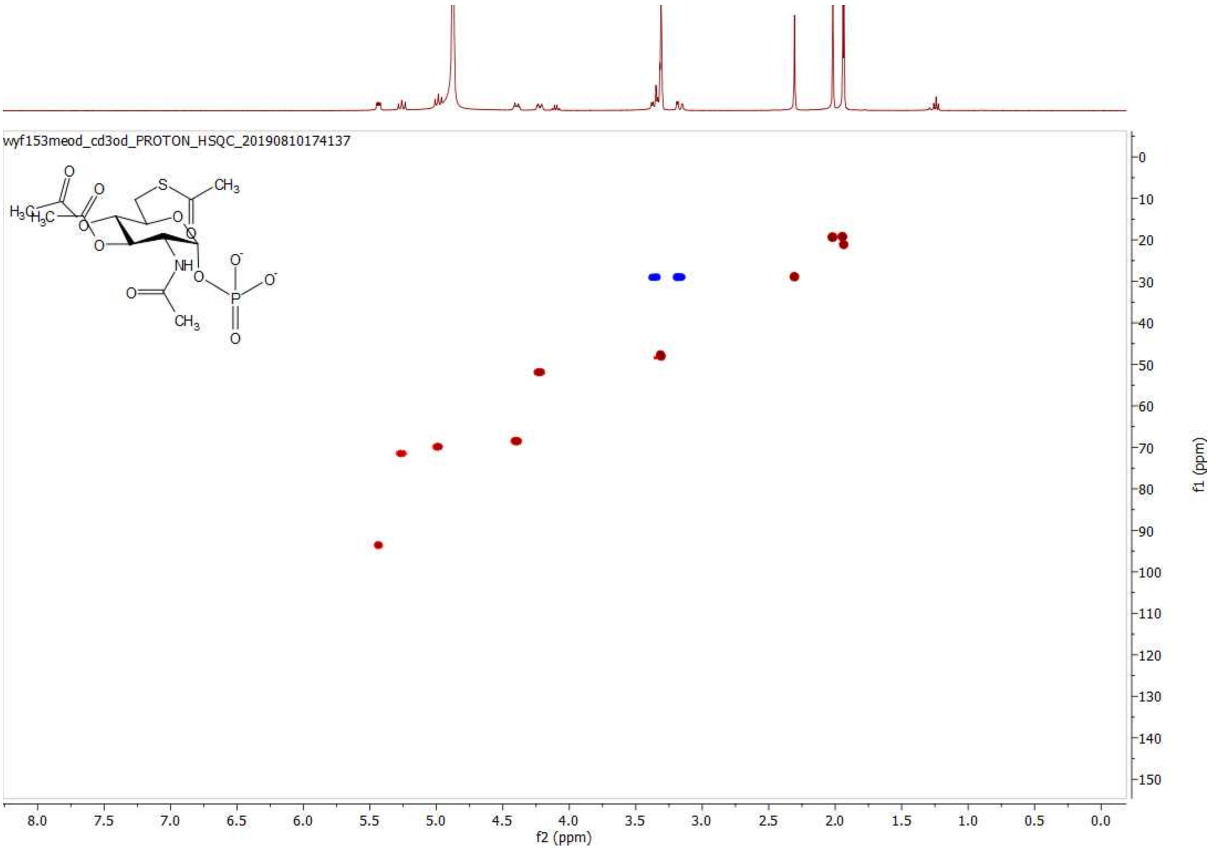

HSQC of 8; 600 MHz/150 MHz, MeOD

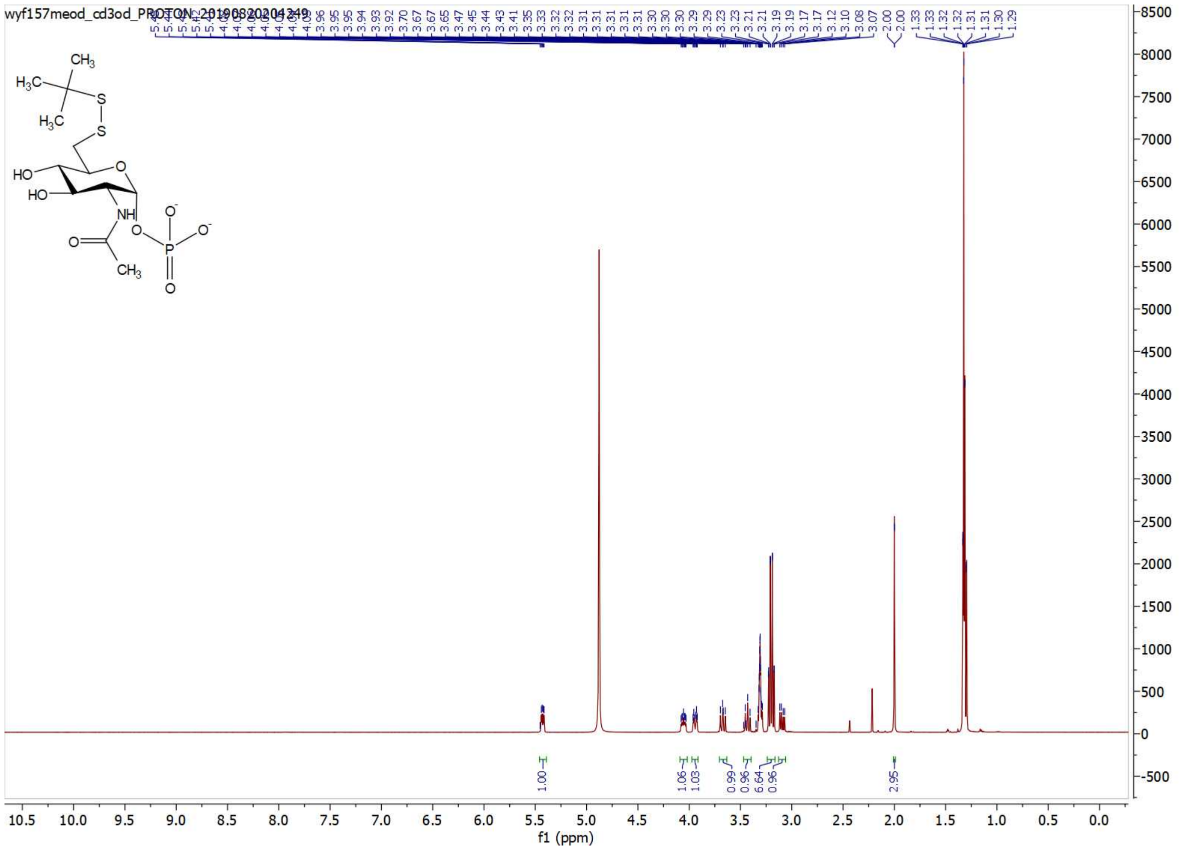

^{1}H NMR of 9; 600 MHz; MeOD

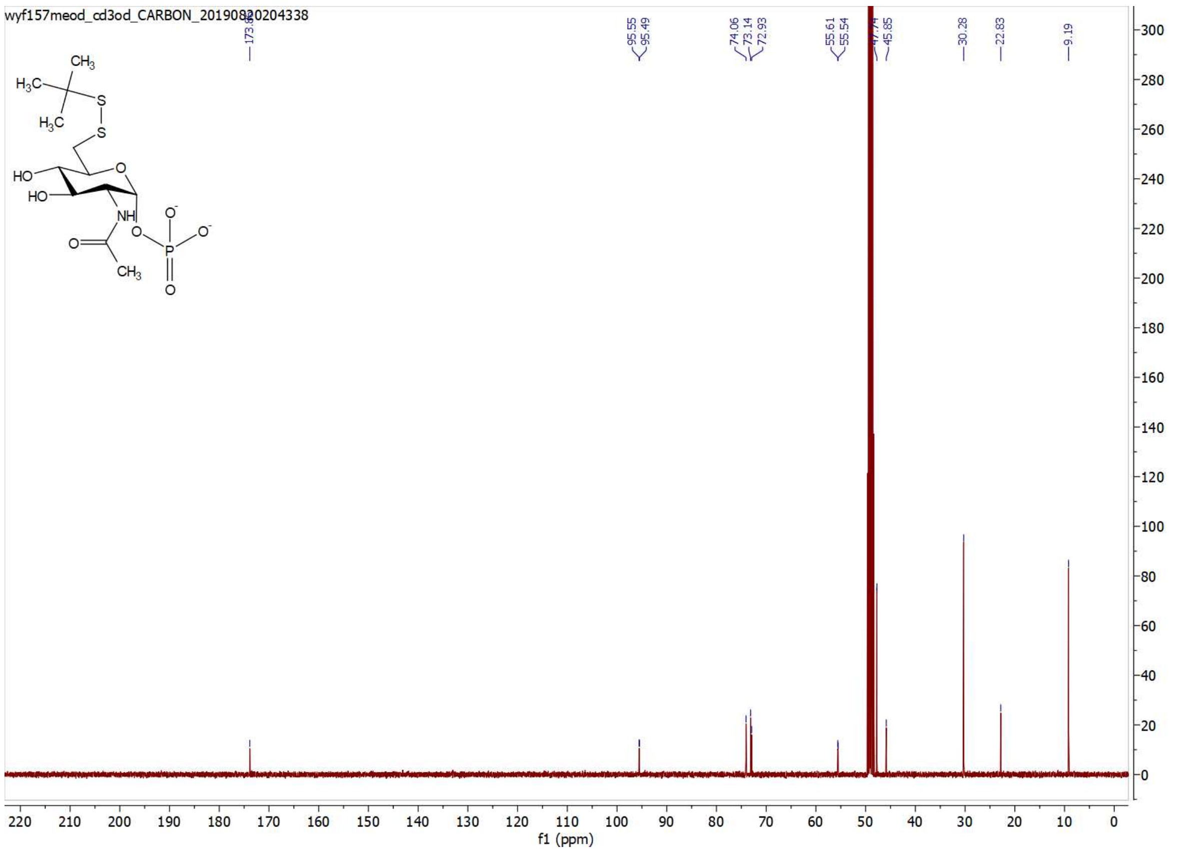

^{13}C NMR of 9; 150 MHz, MeOD

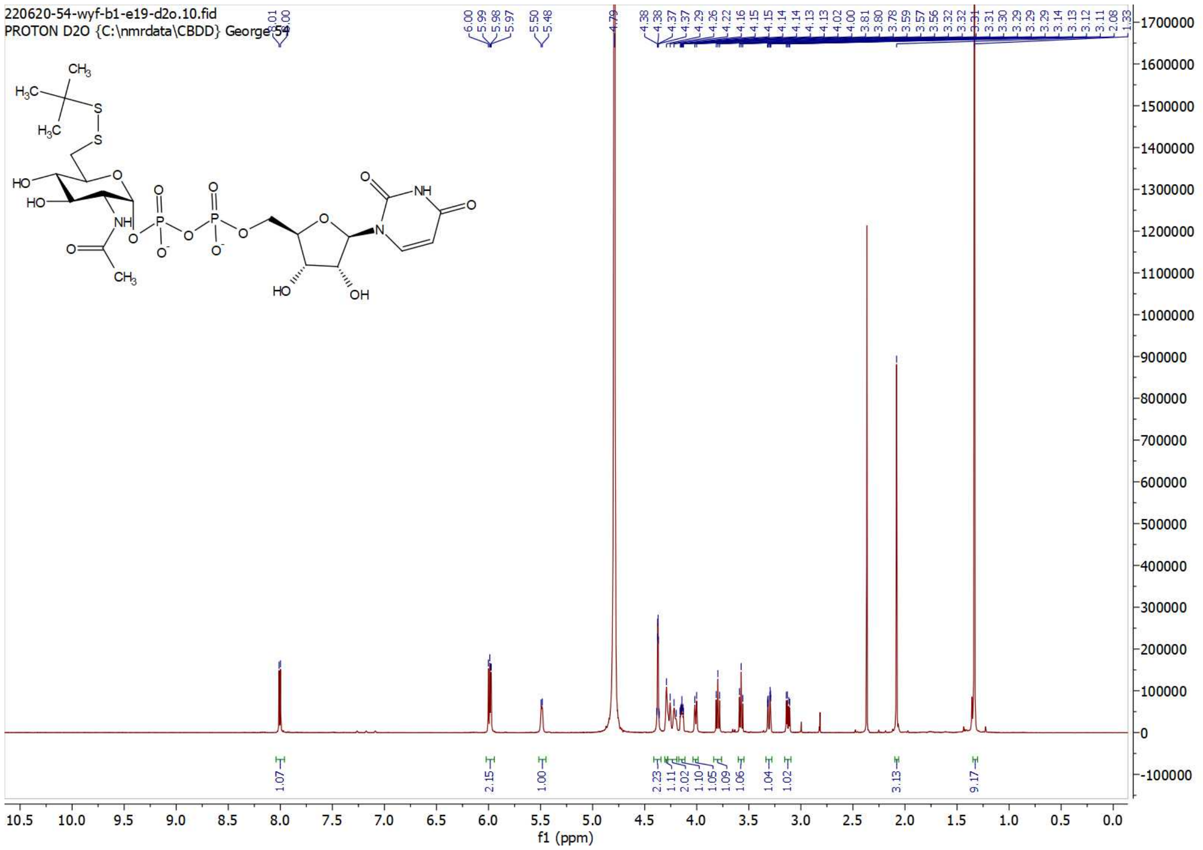

^{1}H NMR of 3; 600 MHz; MeOD

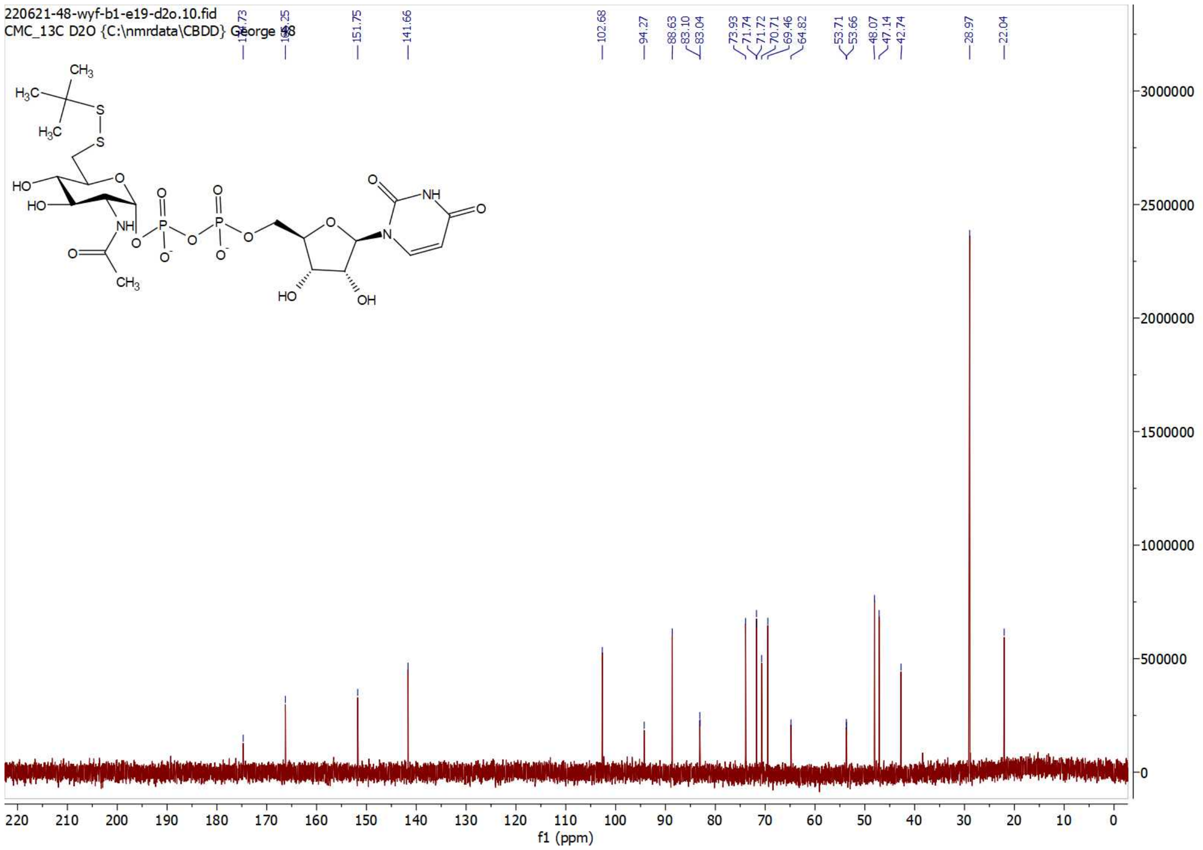

^{13}C NMR of 3; 150 MHz, MeOD

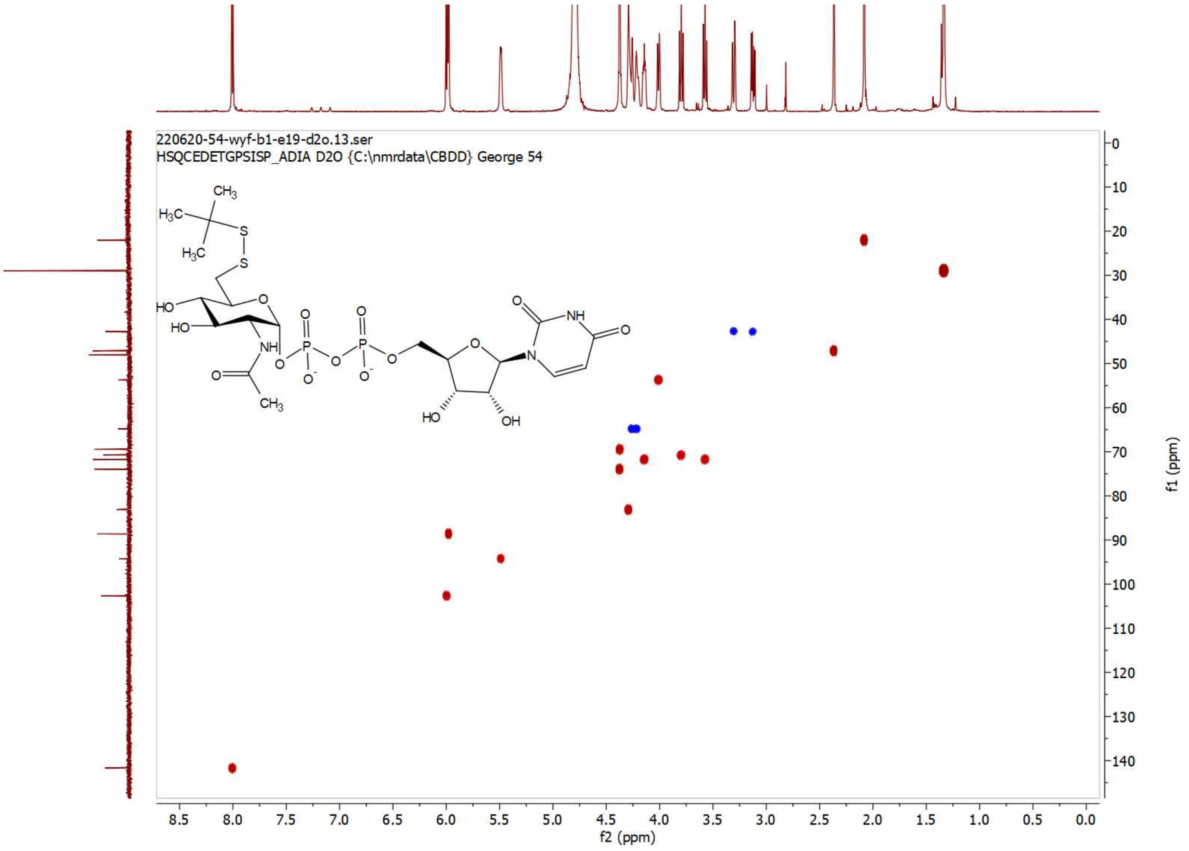

HSQC of 3; 600 MHz/150 MHz, MeOD

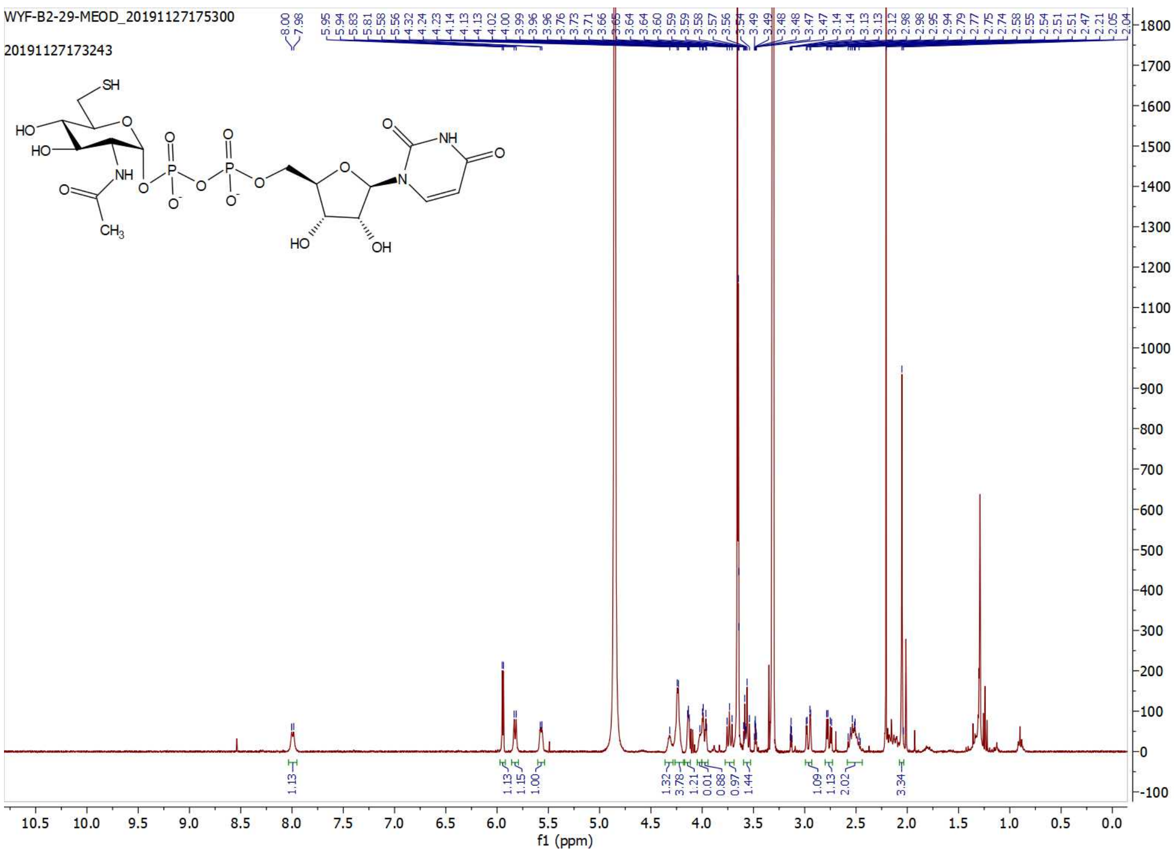

^{1}H NMR of 1; 600 MHz; MeOD

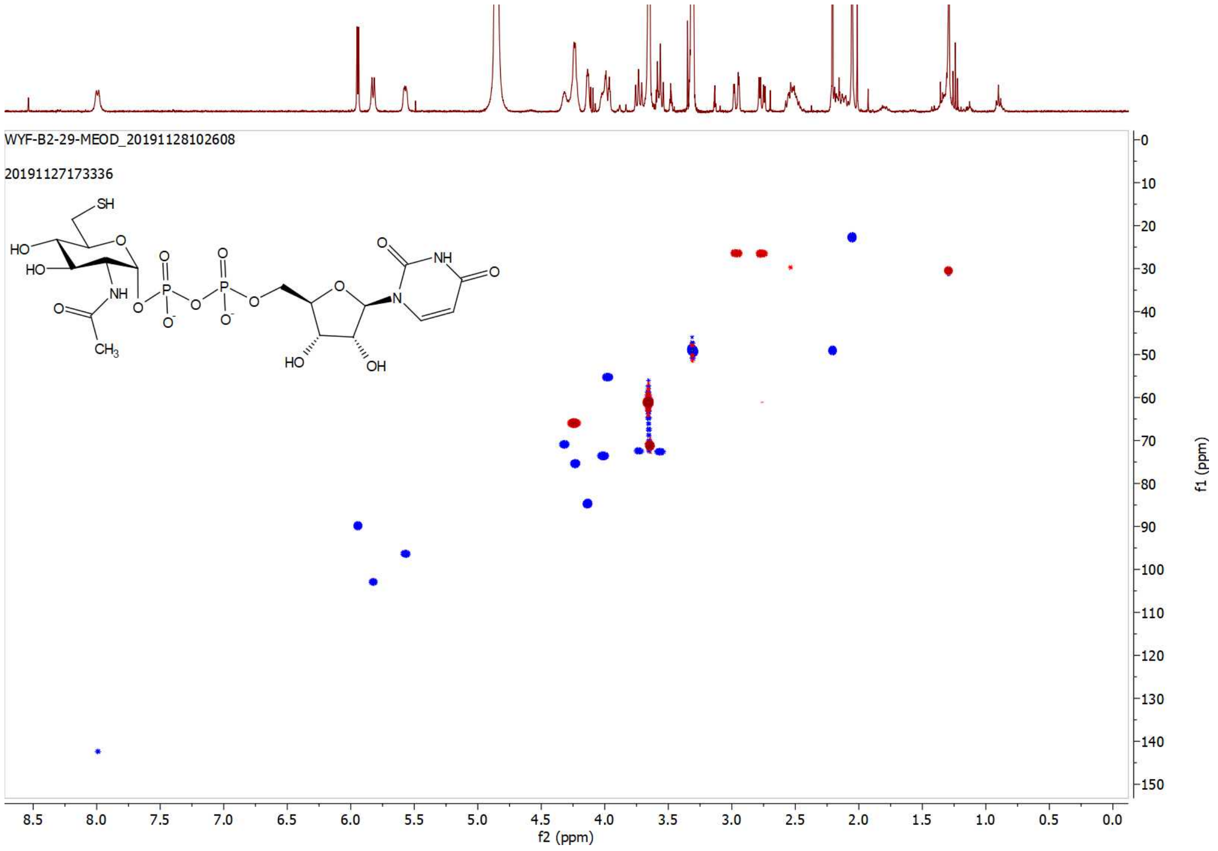

HSQC of 1; 600 MHz/150 MHz, MeOD

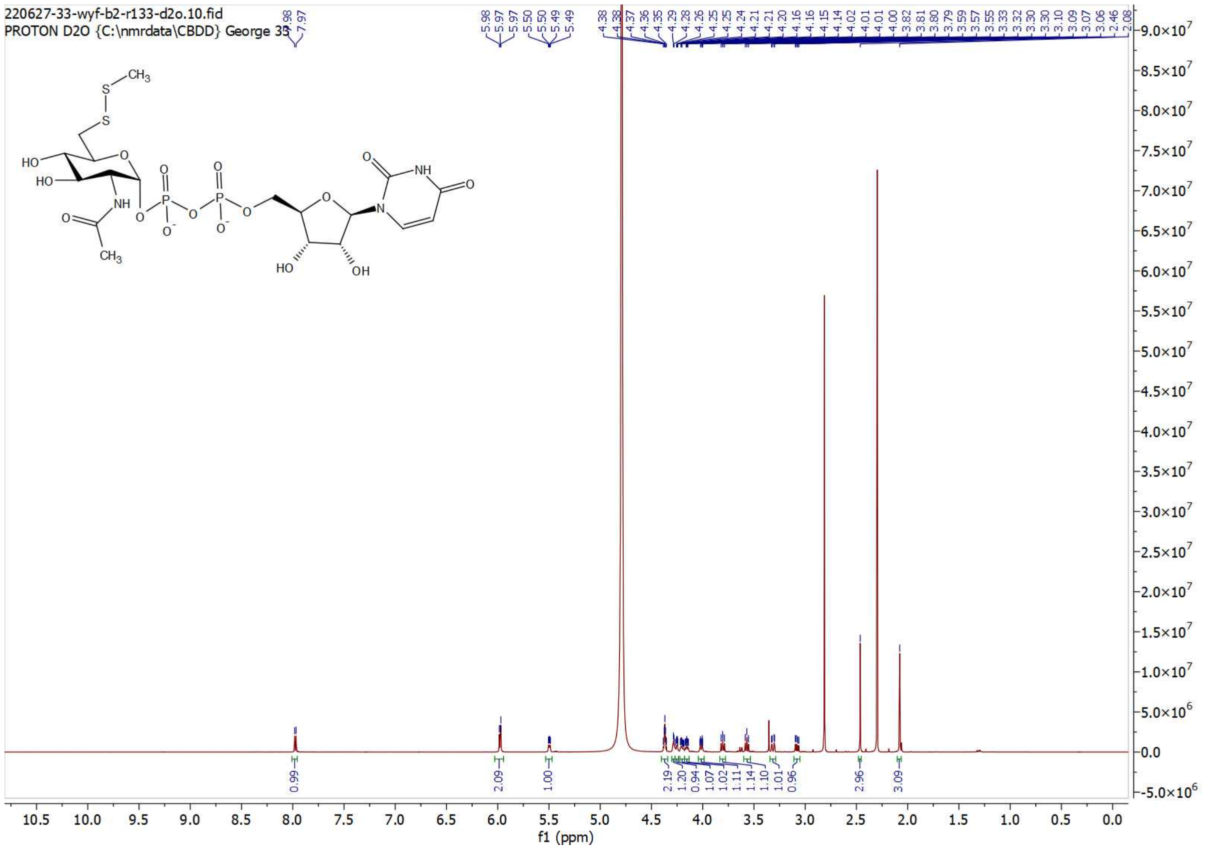

^{1}H NMR of 2; 600 MHz; MeOD

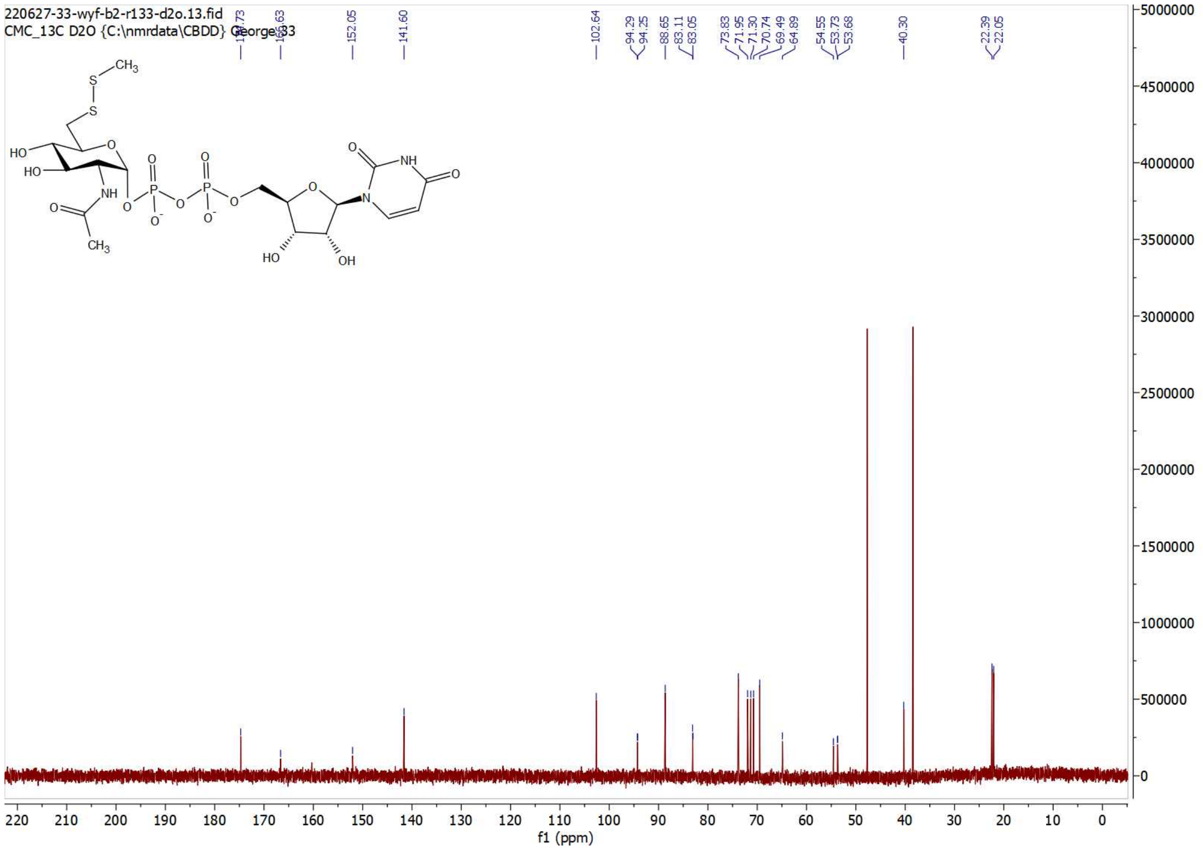

^{13}C NMR of 2; 150 MHz, MeOD

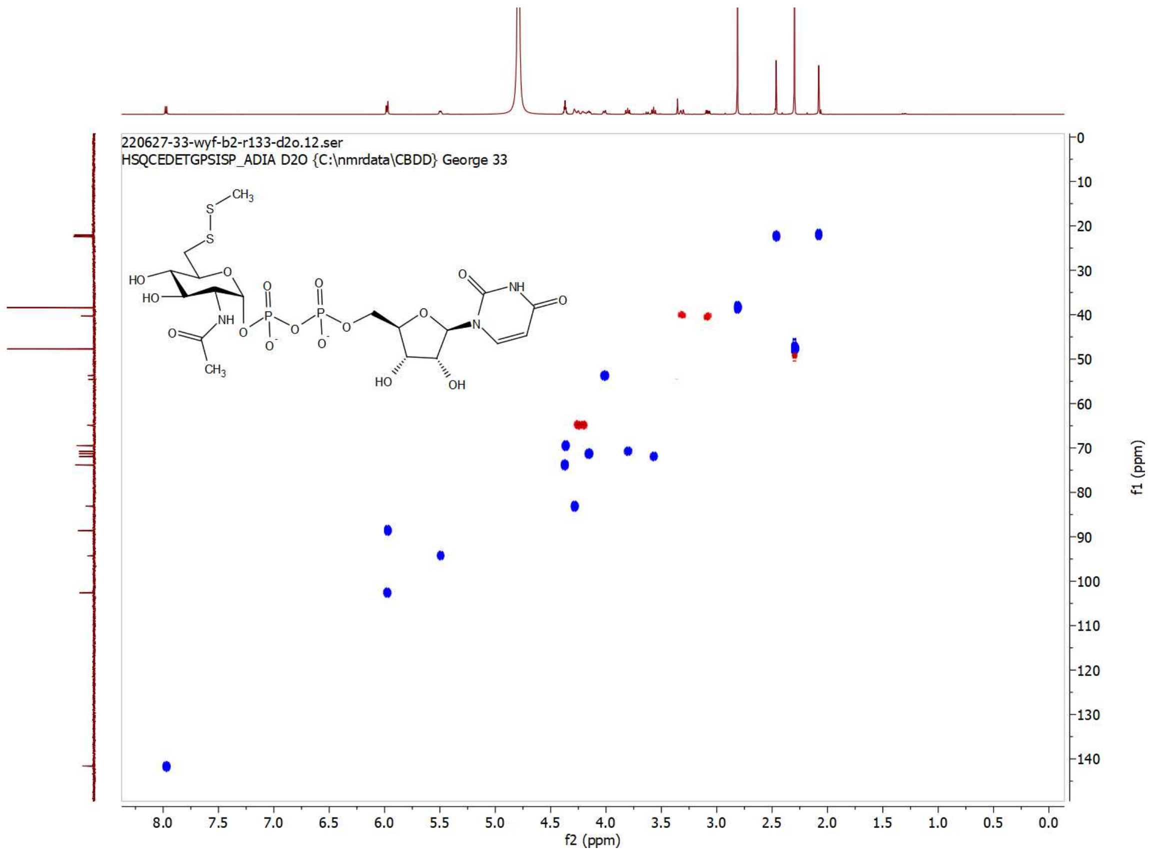

HSQC of 2; 600 MHz/150 MHz, MeOD

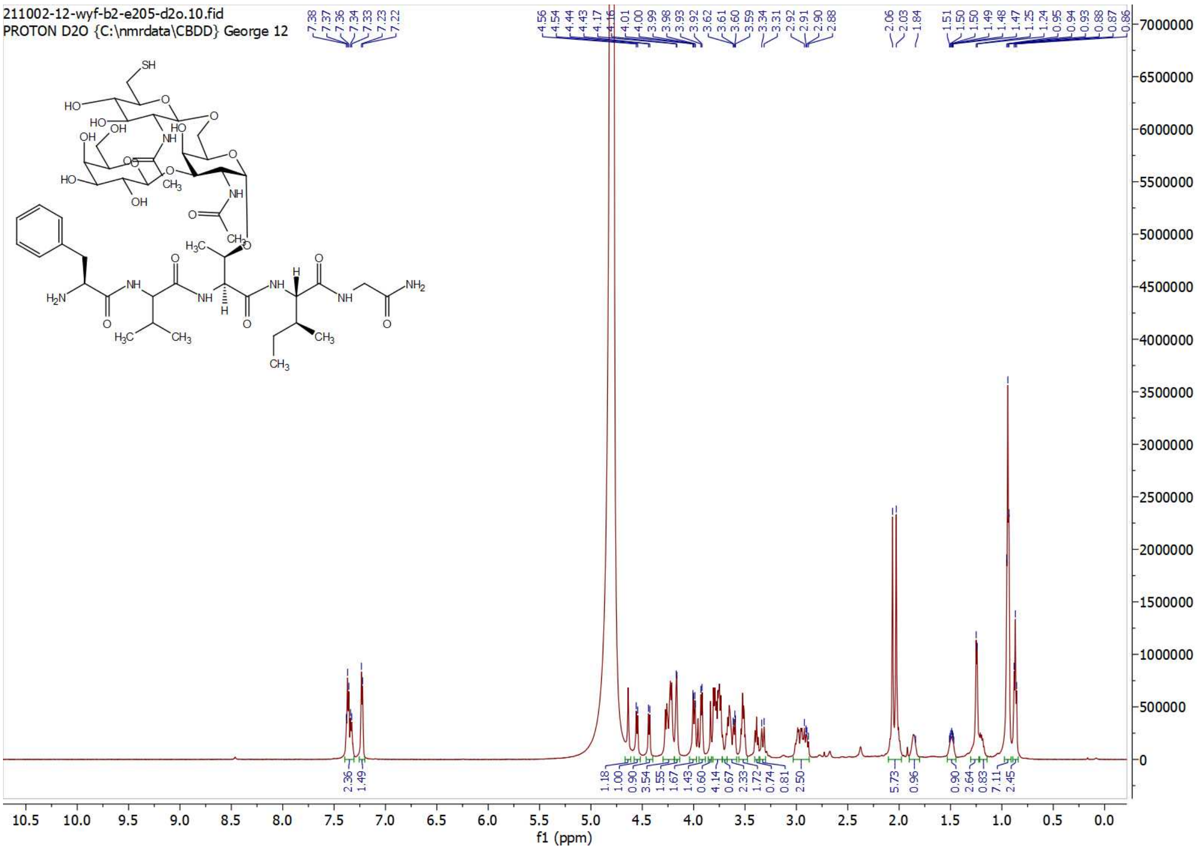

^{1}H NMR of 11a; 600 MHz; D_2O

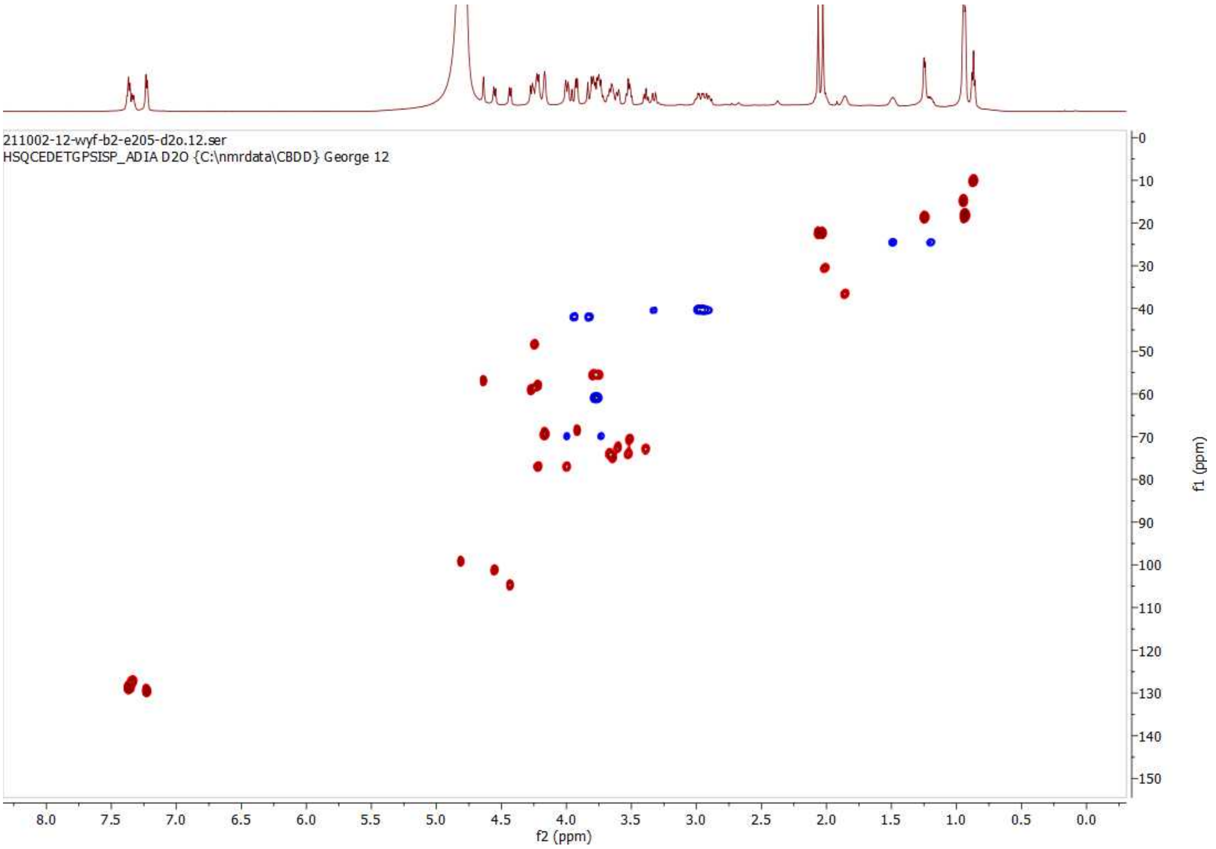

HSQC of 11a; 600 MHz/150 MHz, D_2O

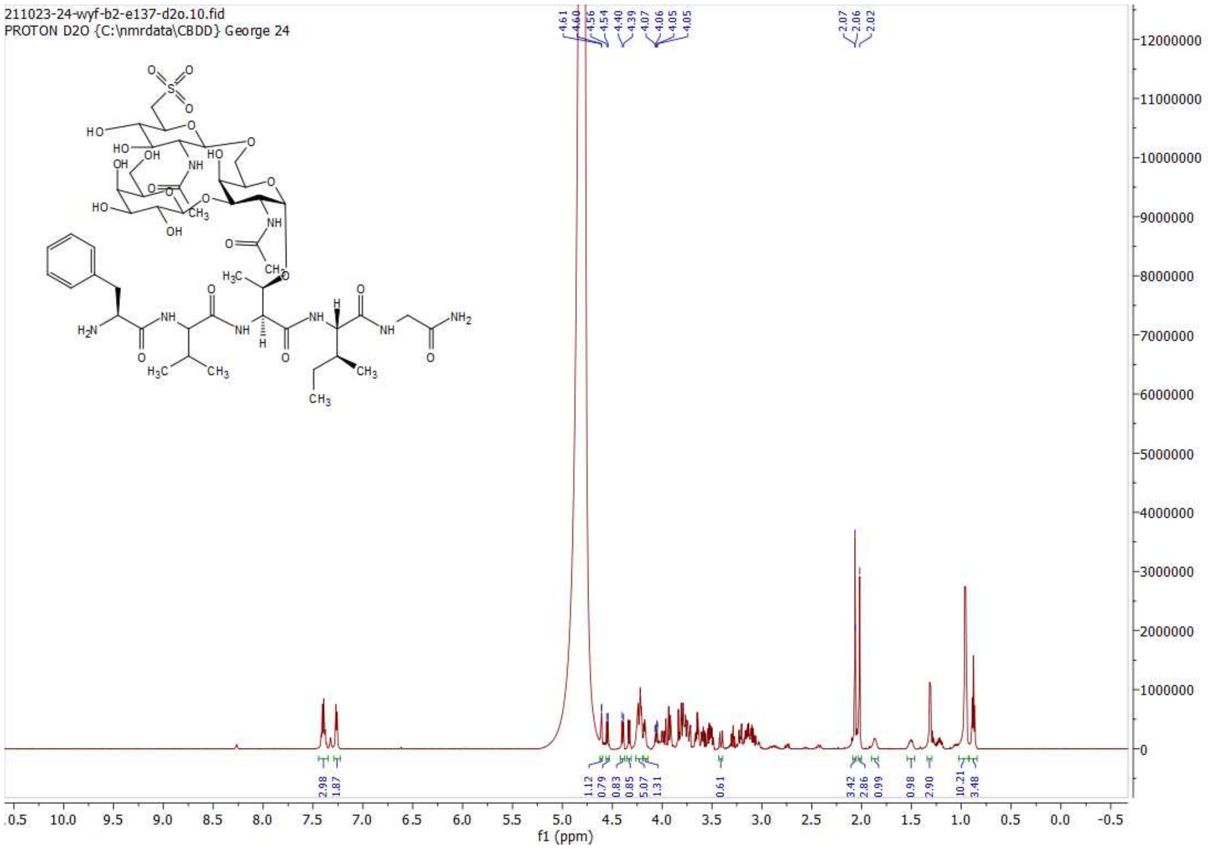

^{1}H NMR of 12; 600 MHz; D_2O

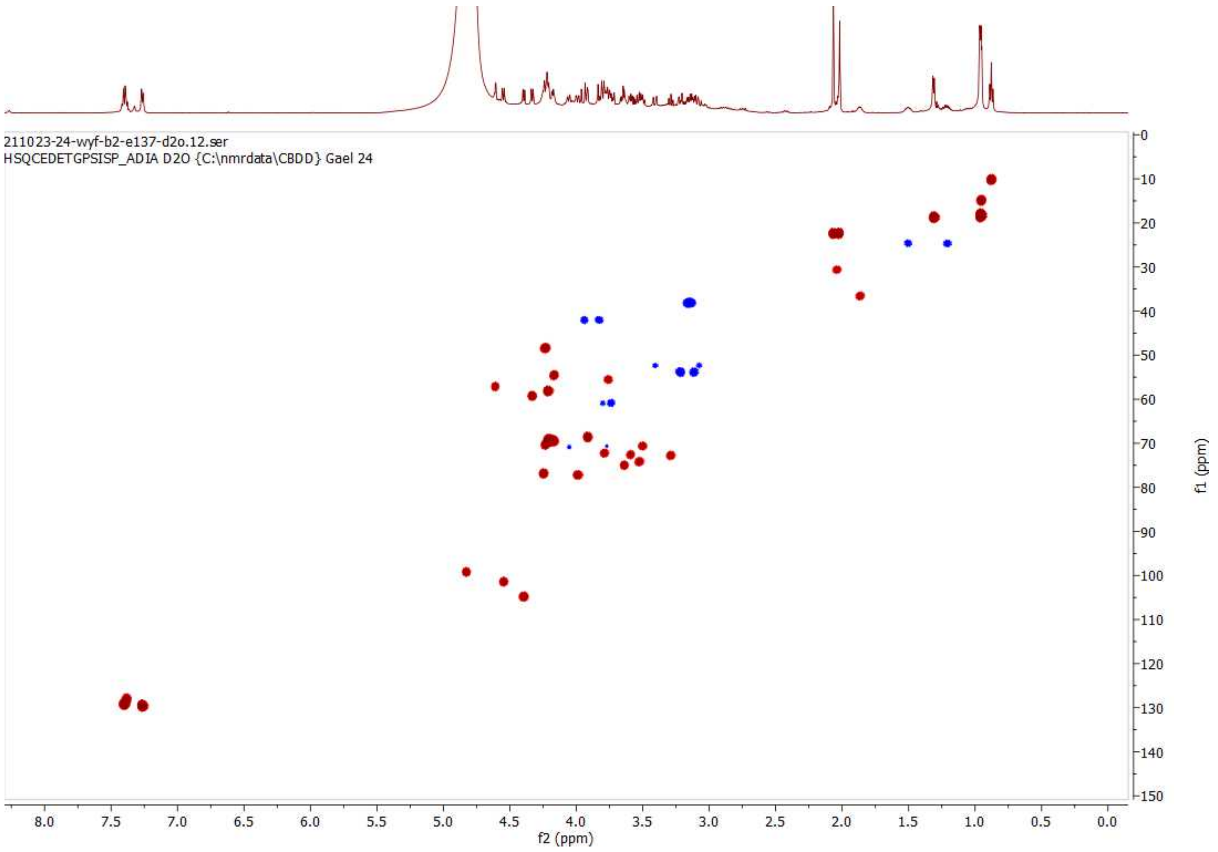

HSQC of 12; 600 MHz/150 MHz, D_2O

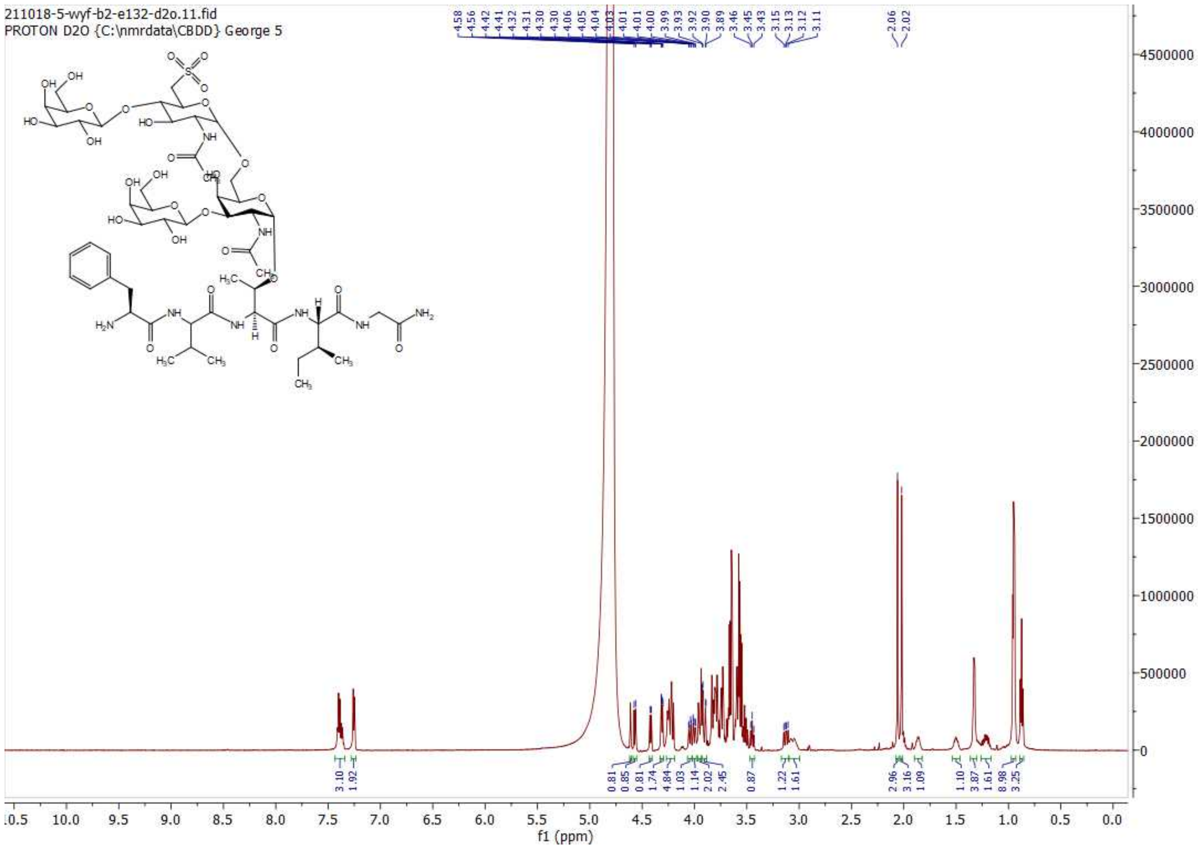

^{1}H NMR of 13; 600 MHz; D$_2$O

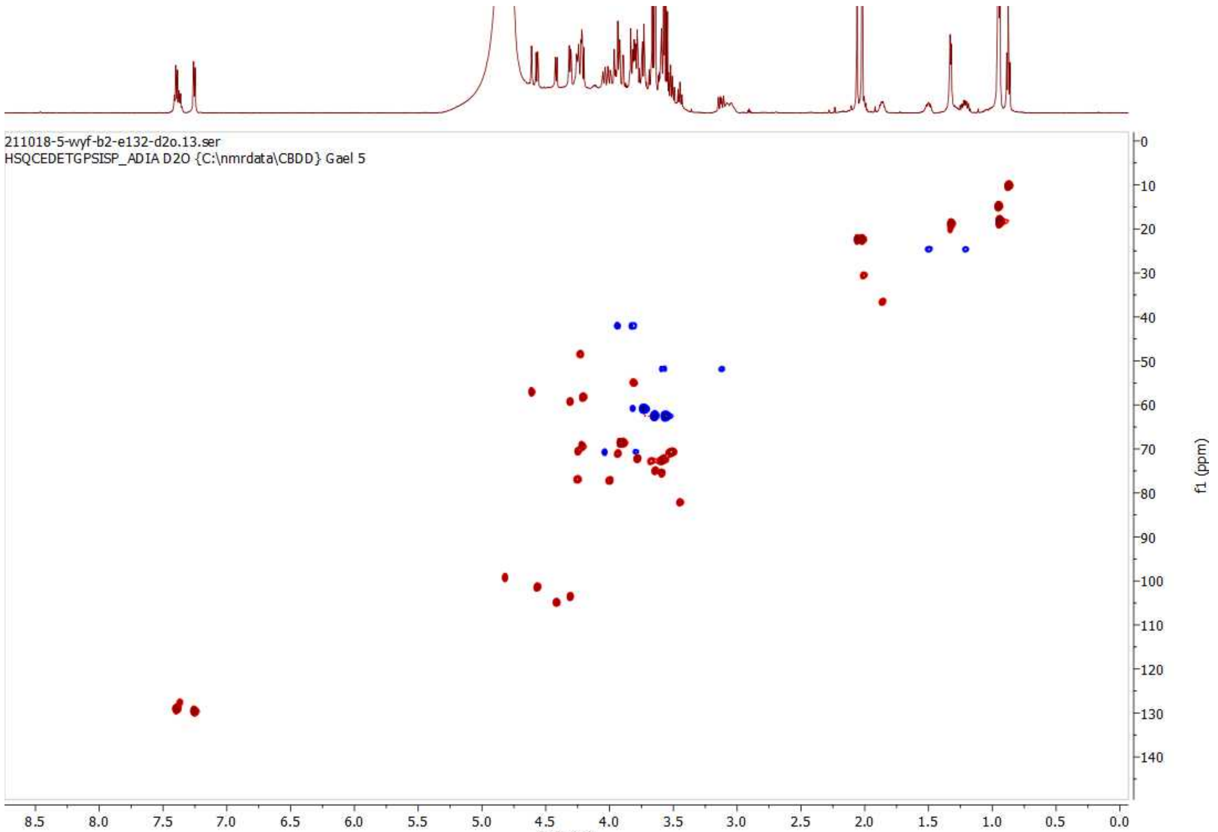

HSQC of 13; 600 MHz/150 MHz, D$_2$O

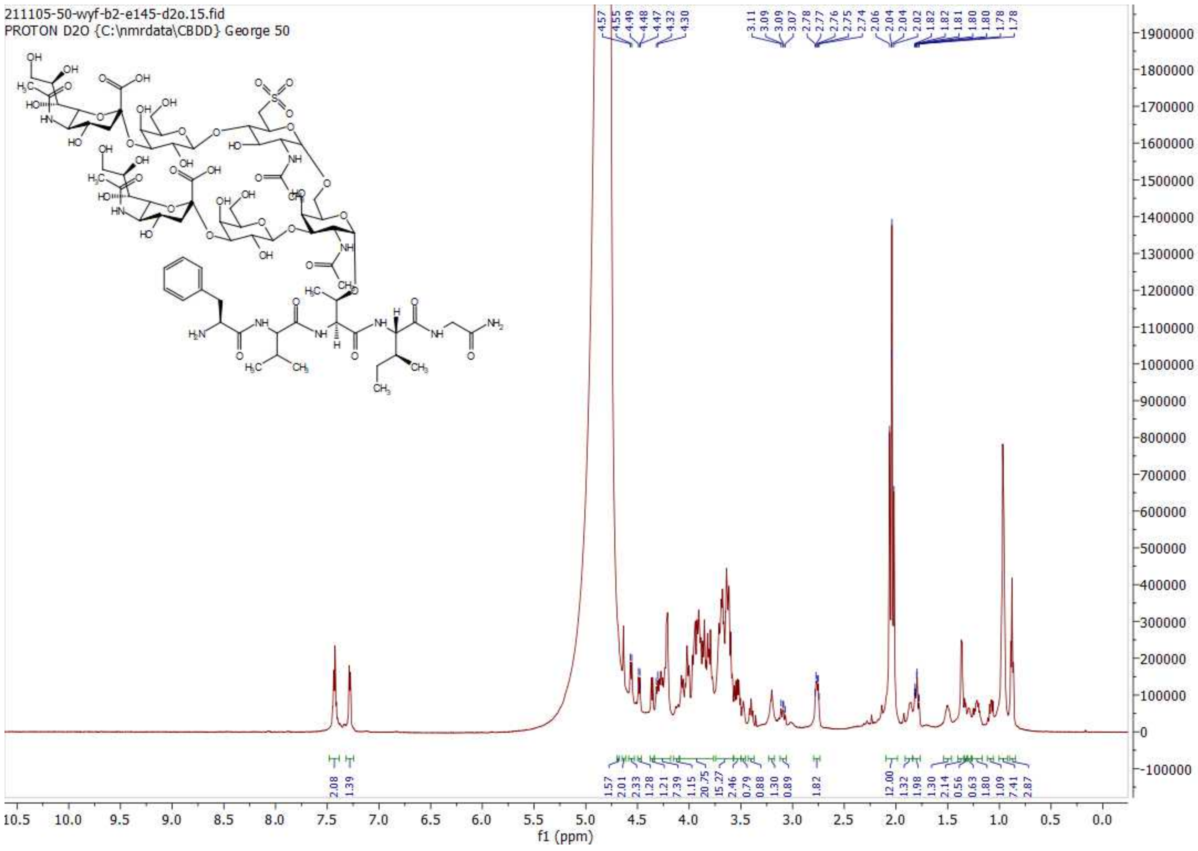

^{1}H NMR of 14; 600 MHz; D_2O

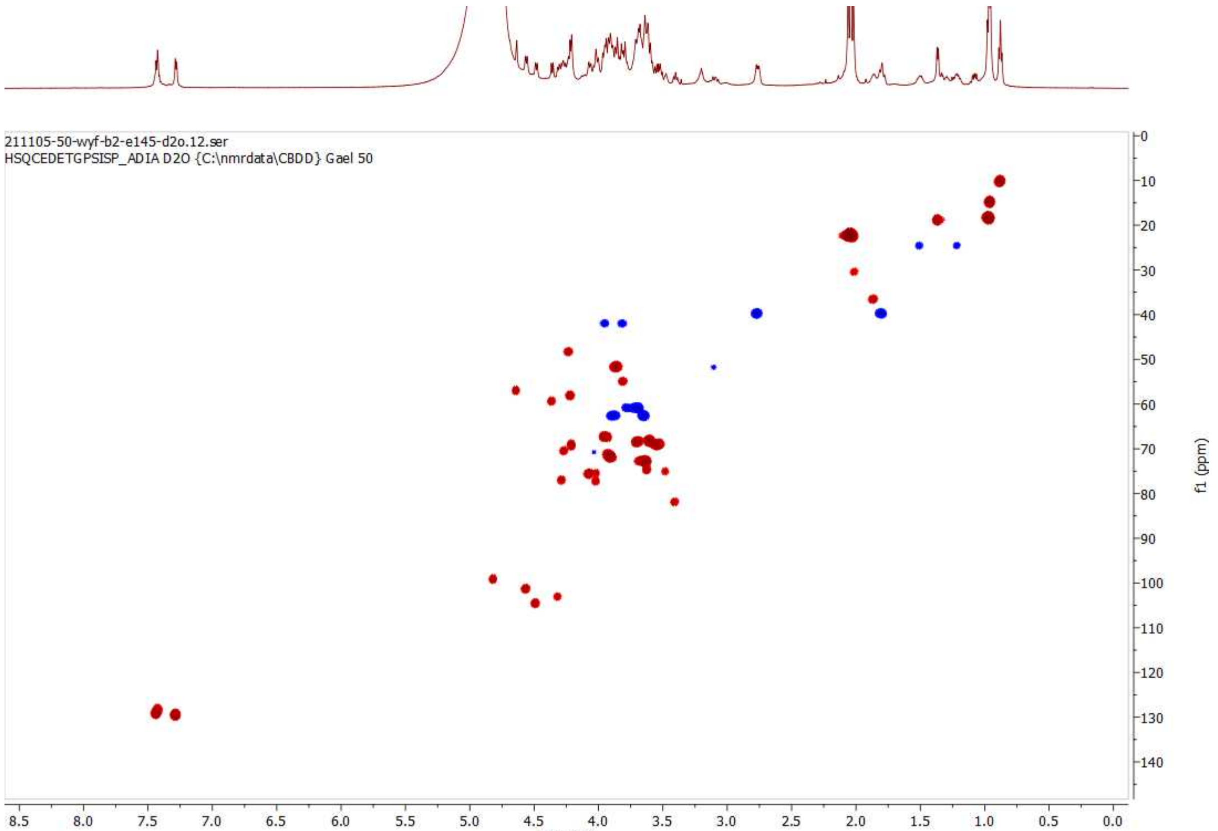

HSQC of 14; 600 MHz/150 MHz, D_2O

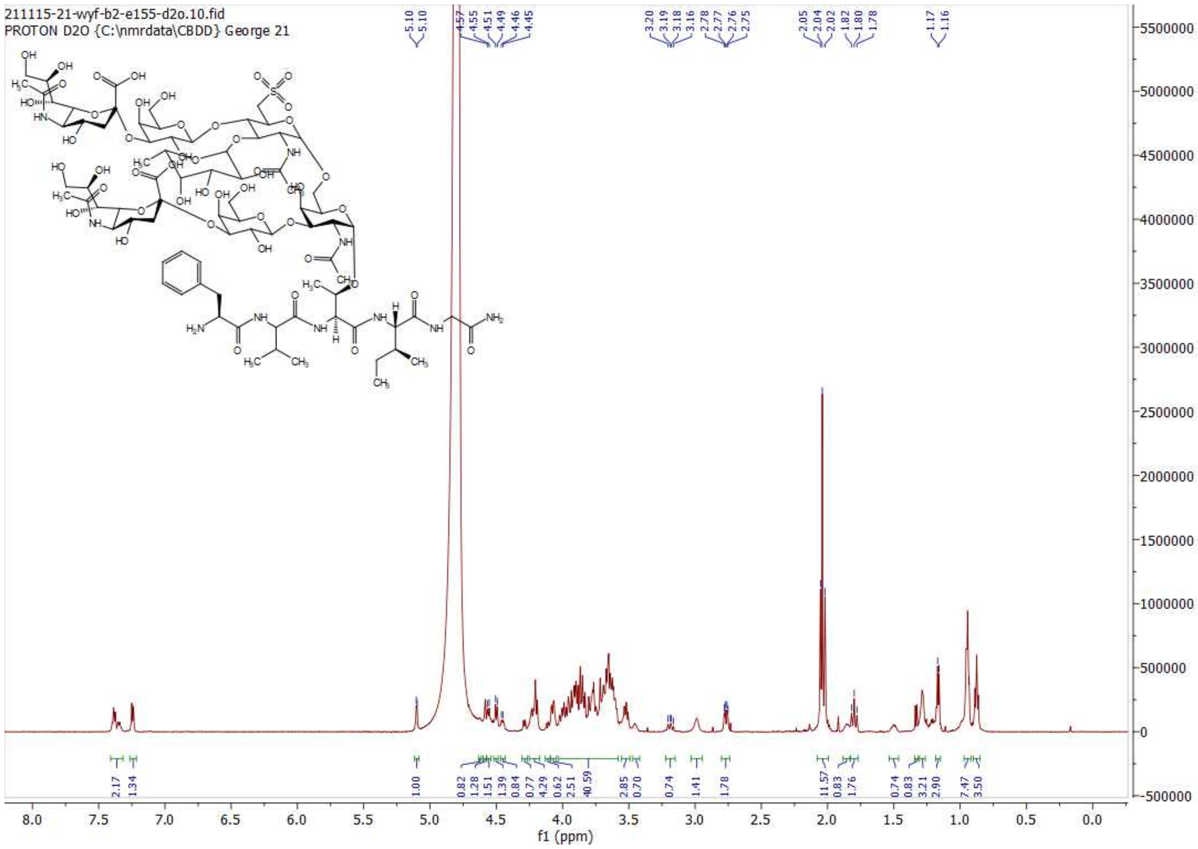

^{1}H NMR of 15; 600 MHz; D_2O**

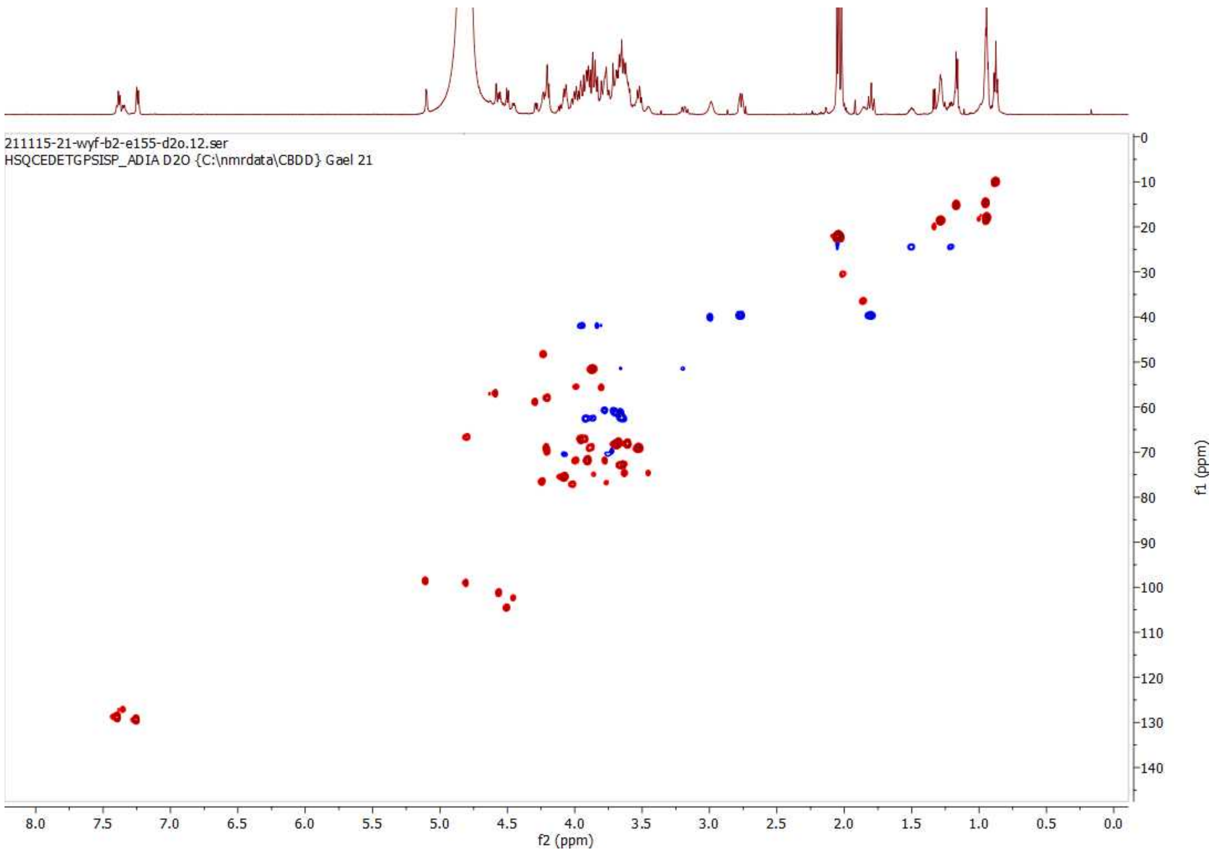

HSQC of 15; 600 MHz/150 MHz, D_2O

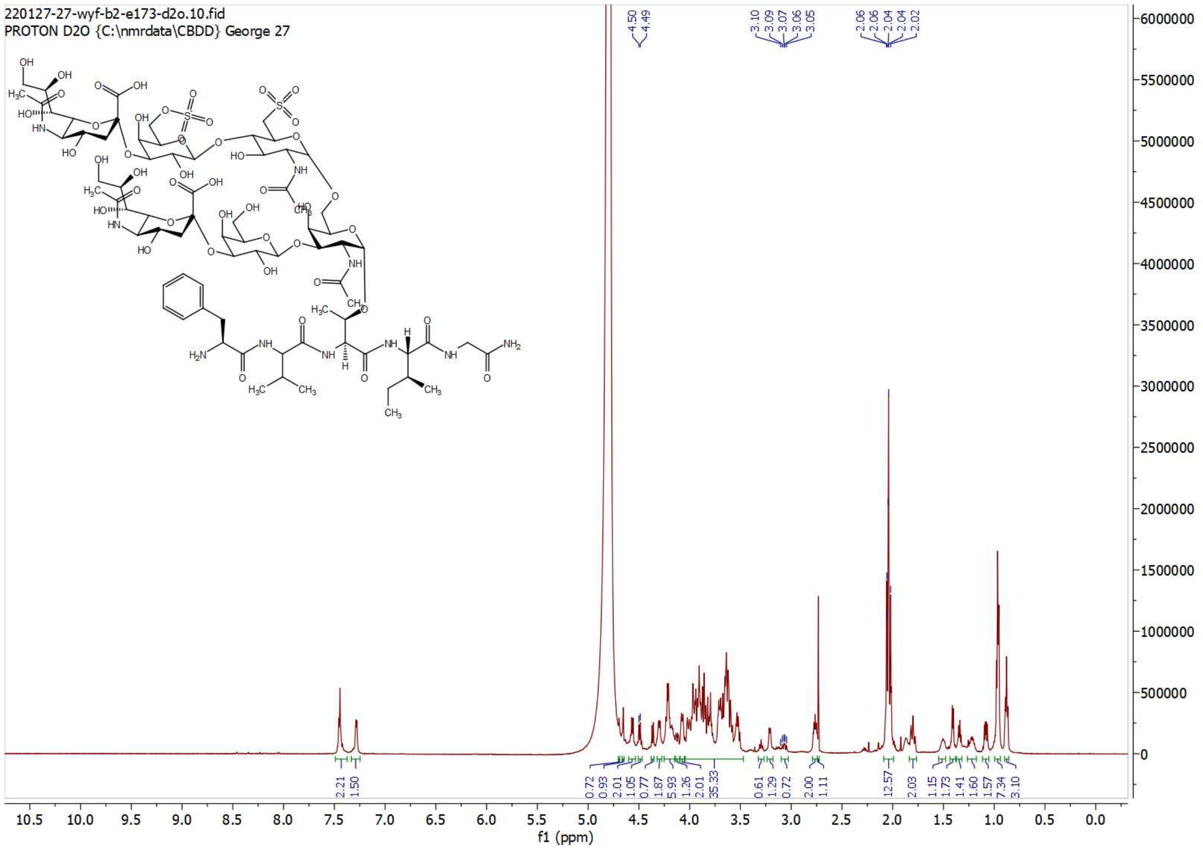

^{1}H NMR of 16; 600 MHz; D_2O

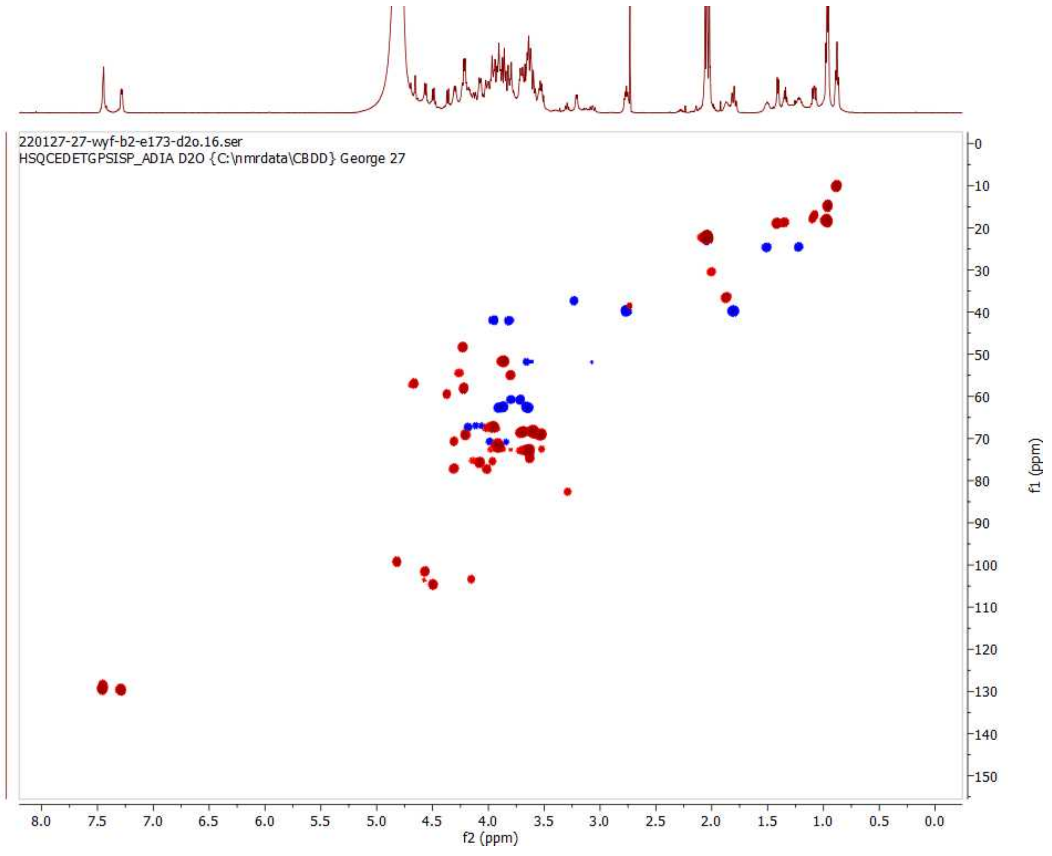

HSQC of 16; 600 MHz/150 MHz, D_2O

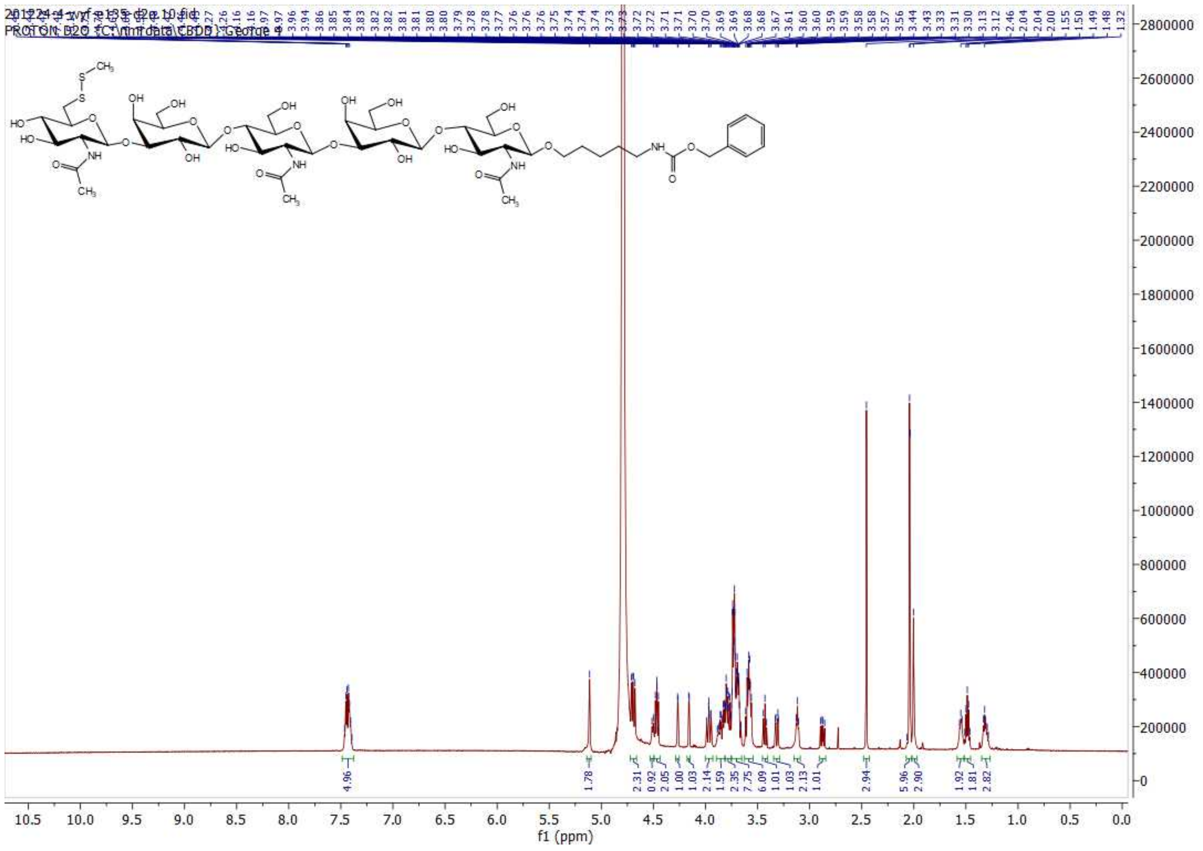

^{1}H NMR of 18b; 600 MHz; D_2O

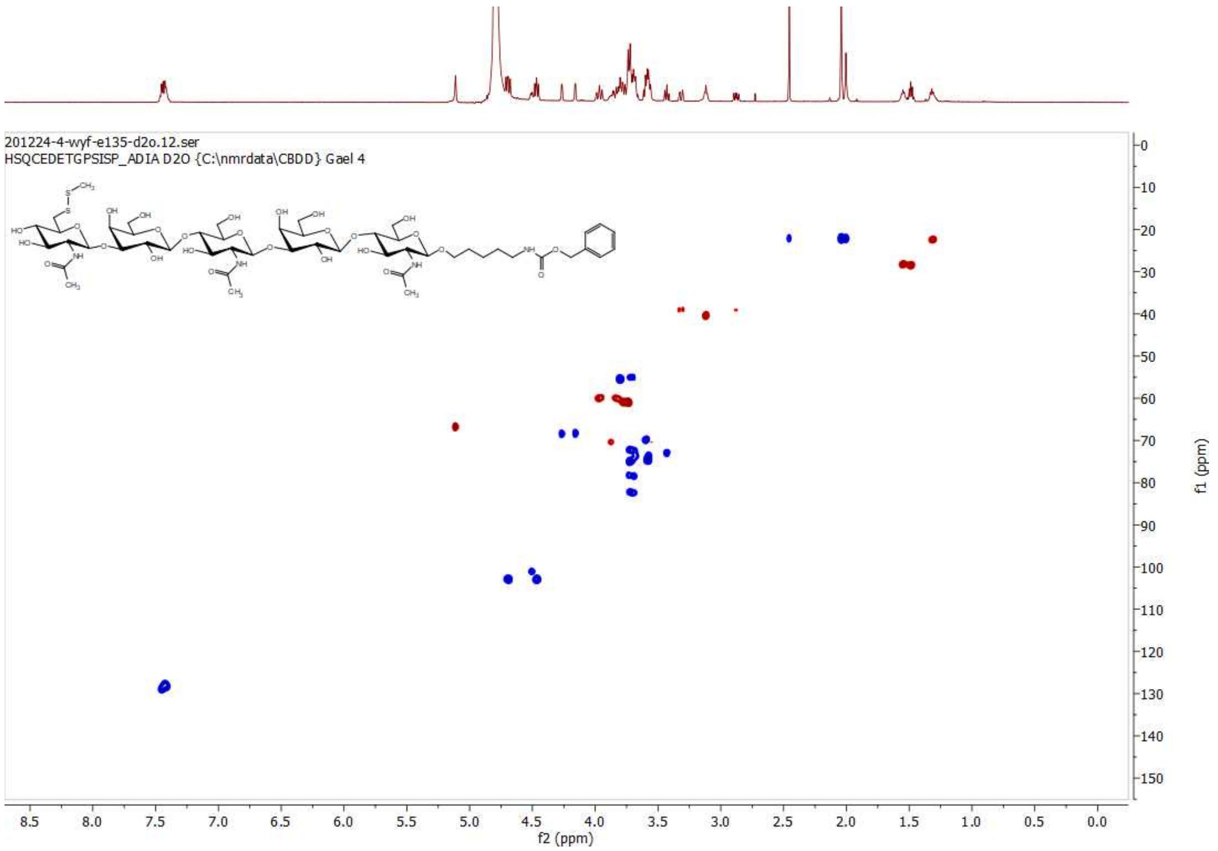

HSQC of 18b; 600 MHz/150 MHz, D_2O

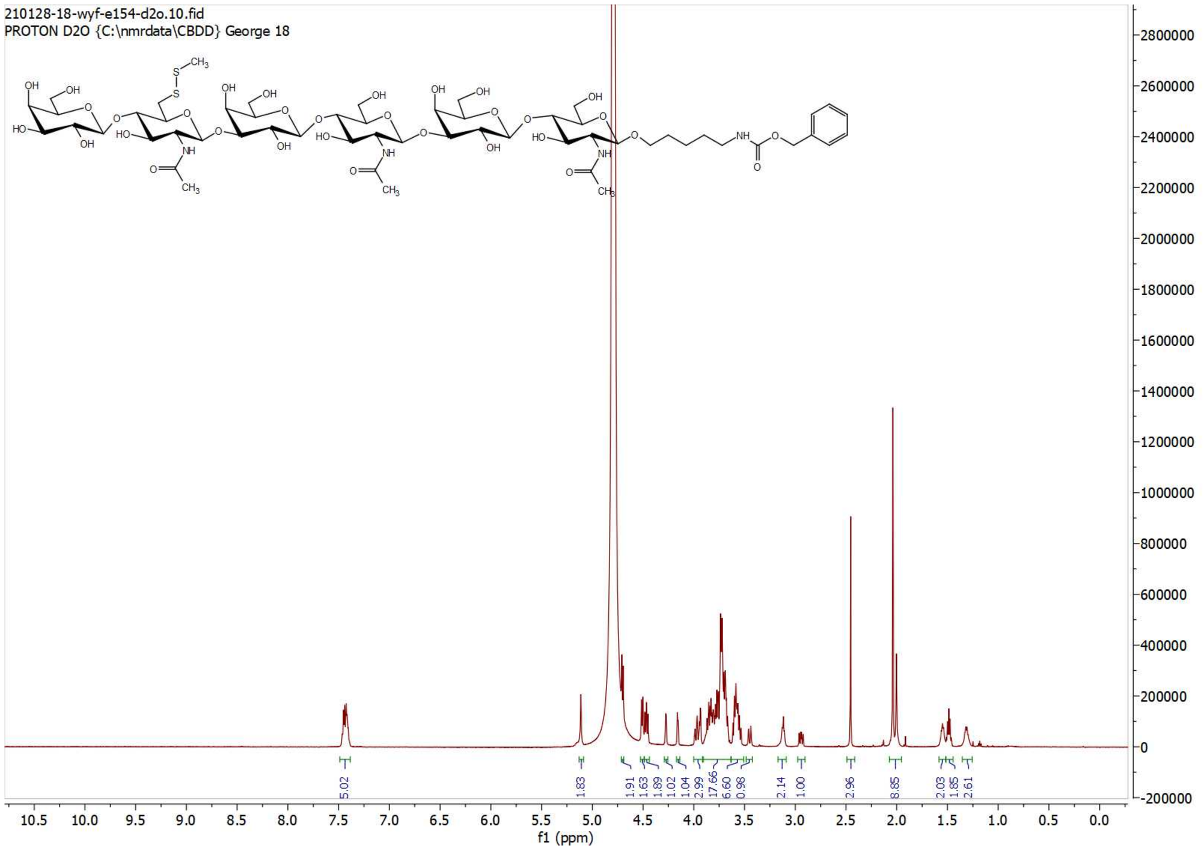

^{1}H NMR of 19; 600 MHz; D_2O

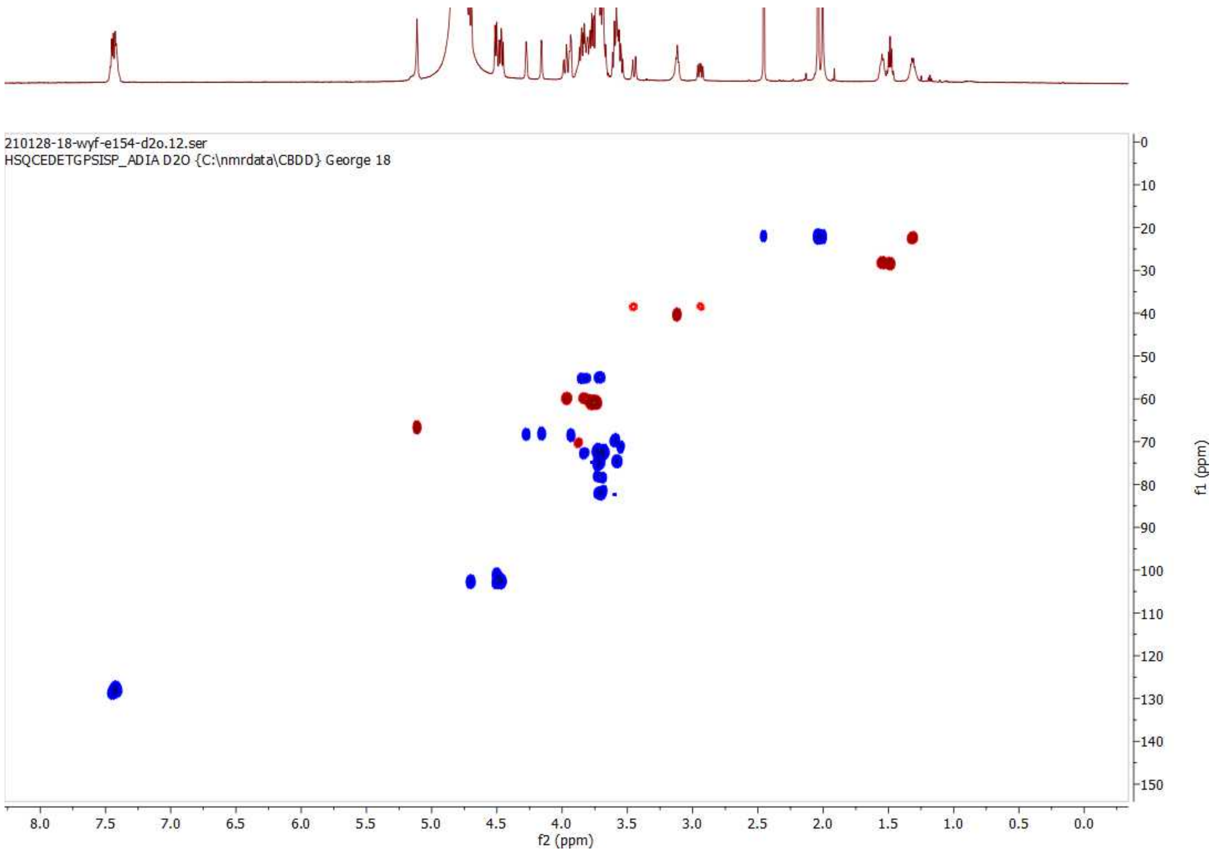

HSQC of 19; 600 MHz/150 MHz, D_2O

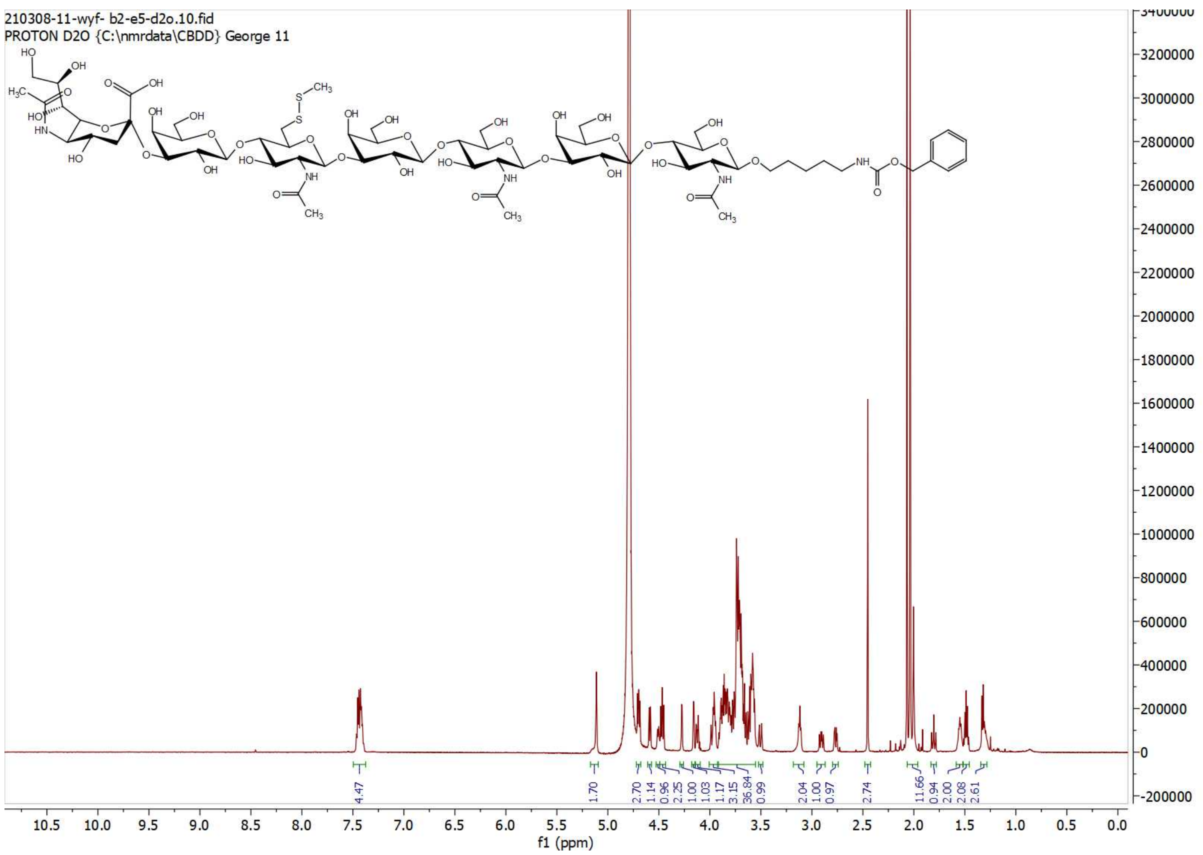

^{1}H NMR of 20; 600 MHz; D$_2$O

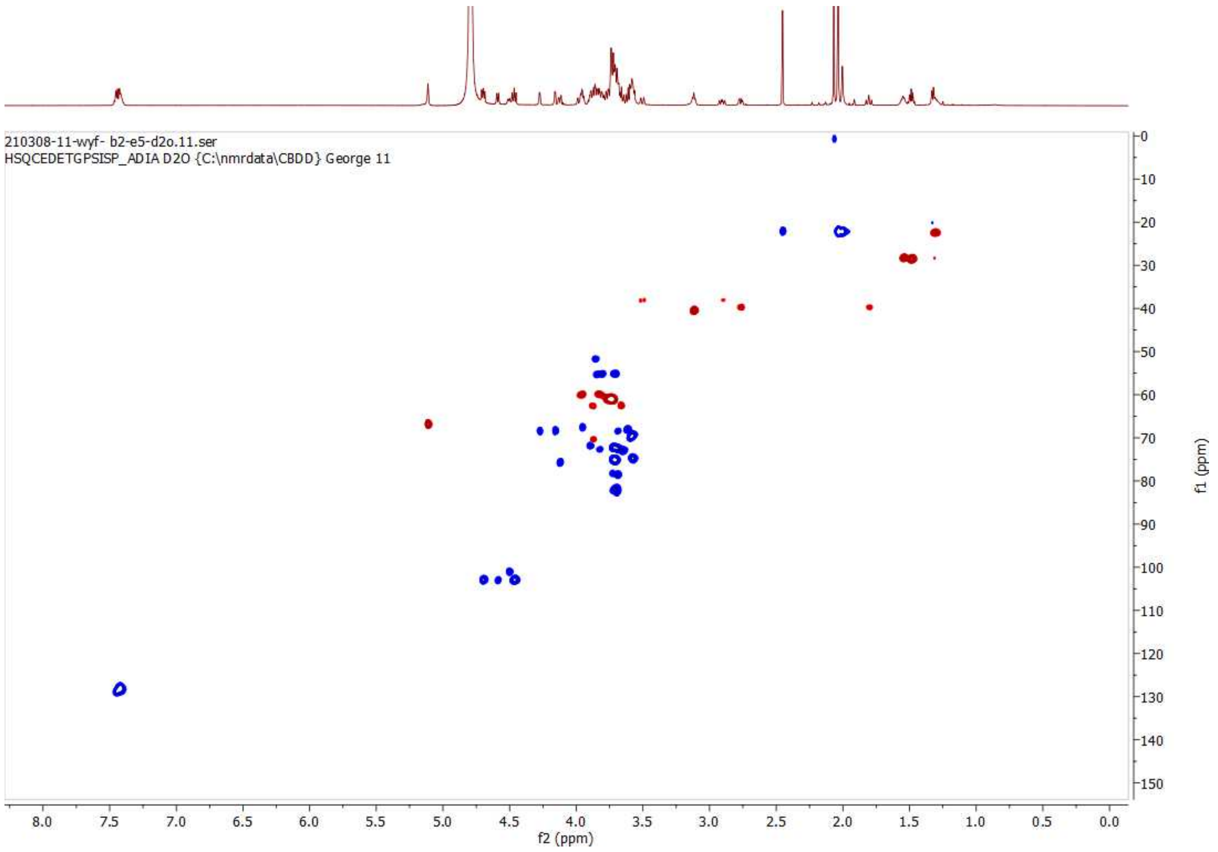

HSQC of 20; 600 MHz/150 MHz, D$_2$O

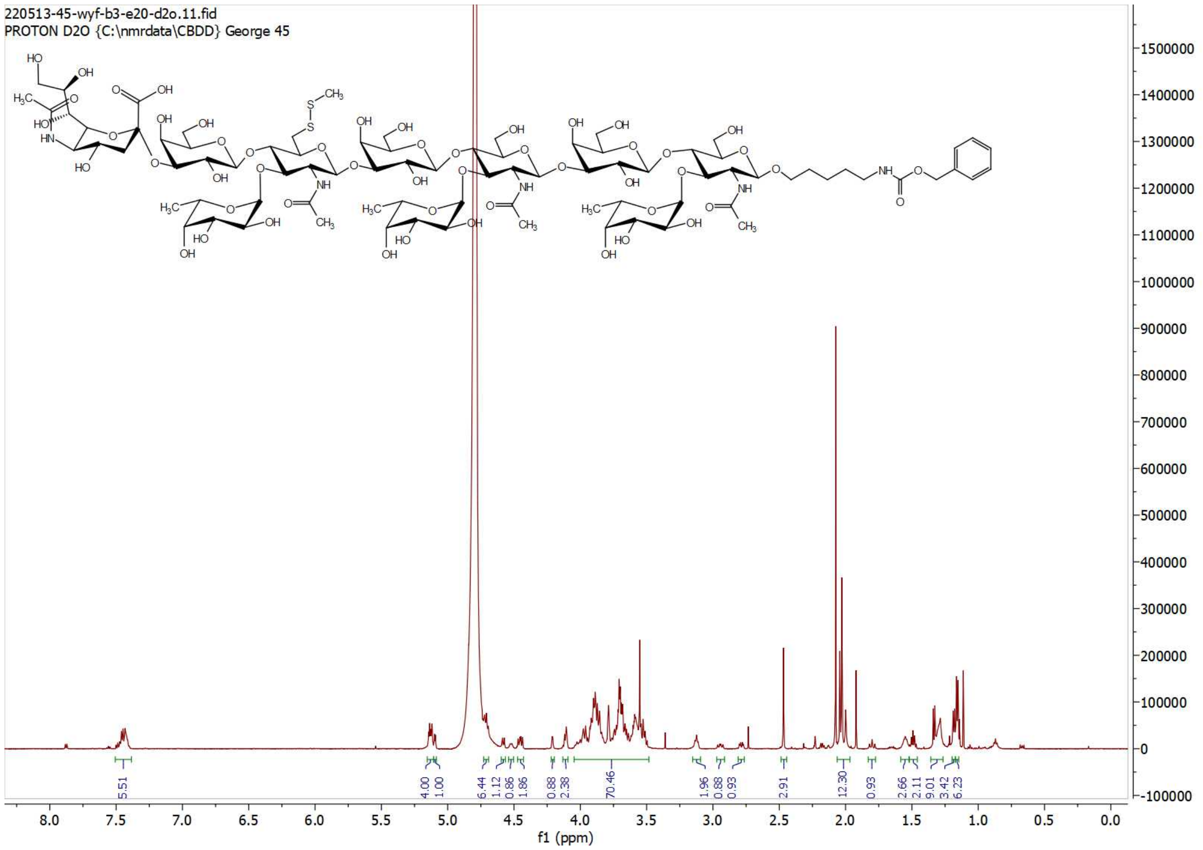

^{1}H NMR of 21; 600 MHz; D_2O

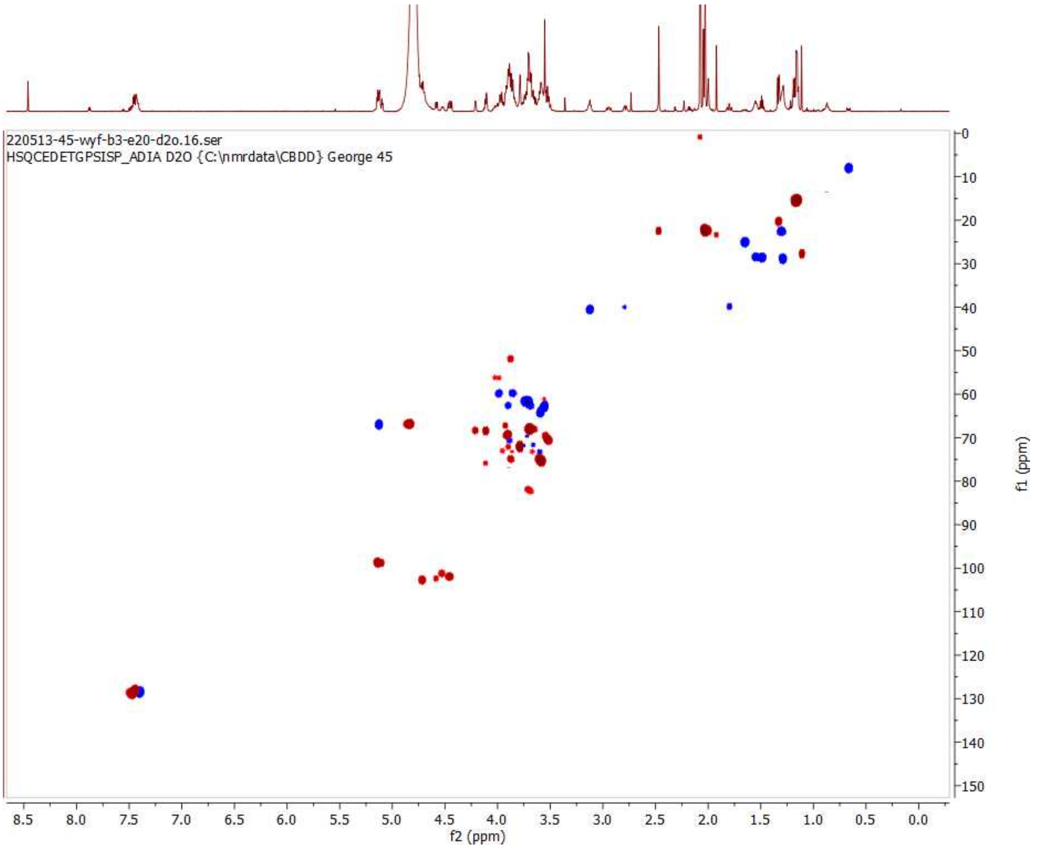

HSQC of 21; 600 MHz/150 MHz, D_2O

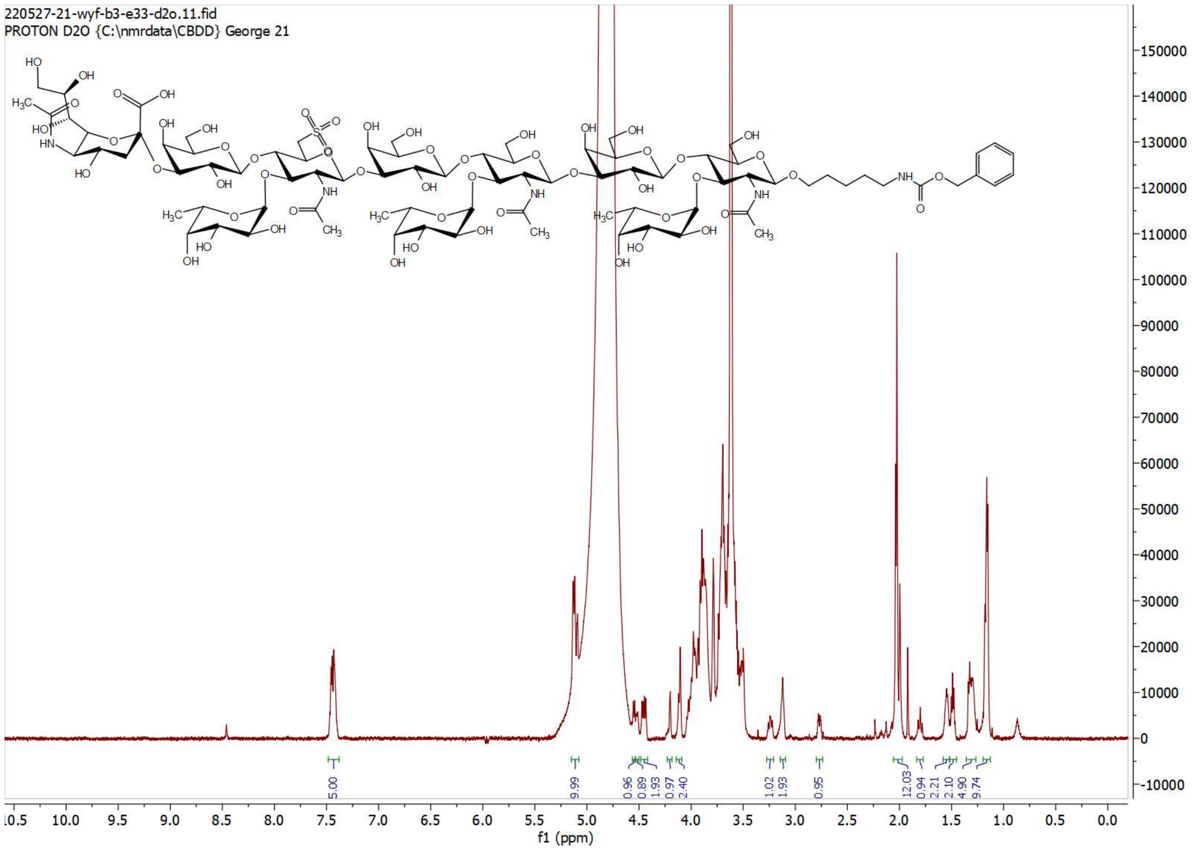

^{1}H NMR of 22; 600 MHz; D_2O

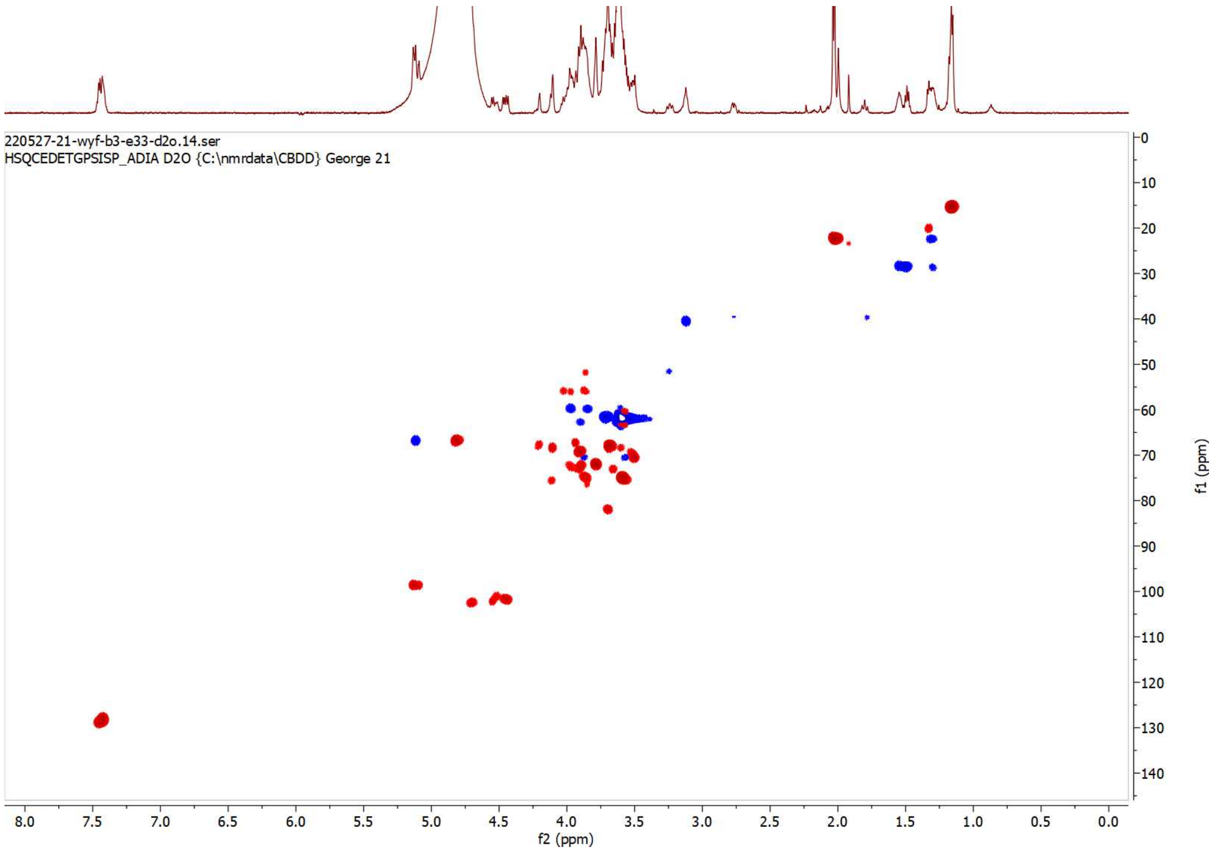

HSQC of 22; 600 MHz/150 MHz, D_2O

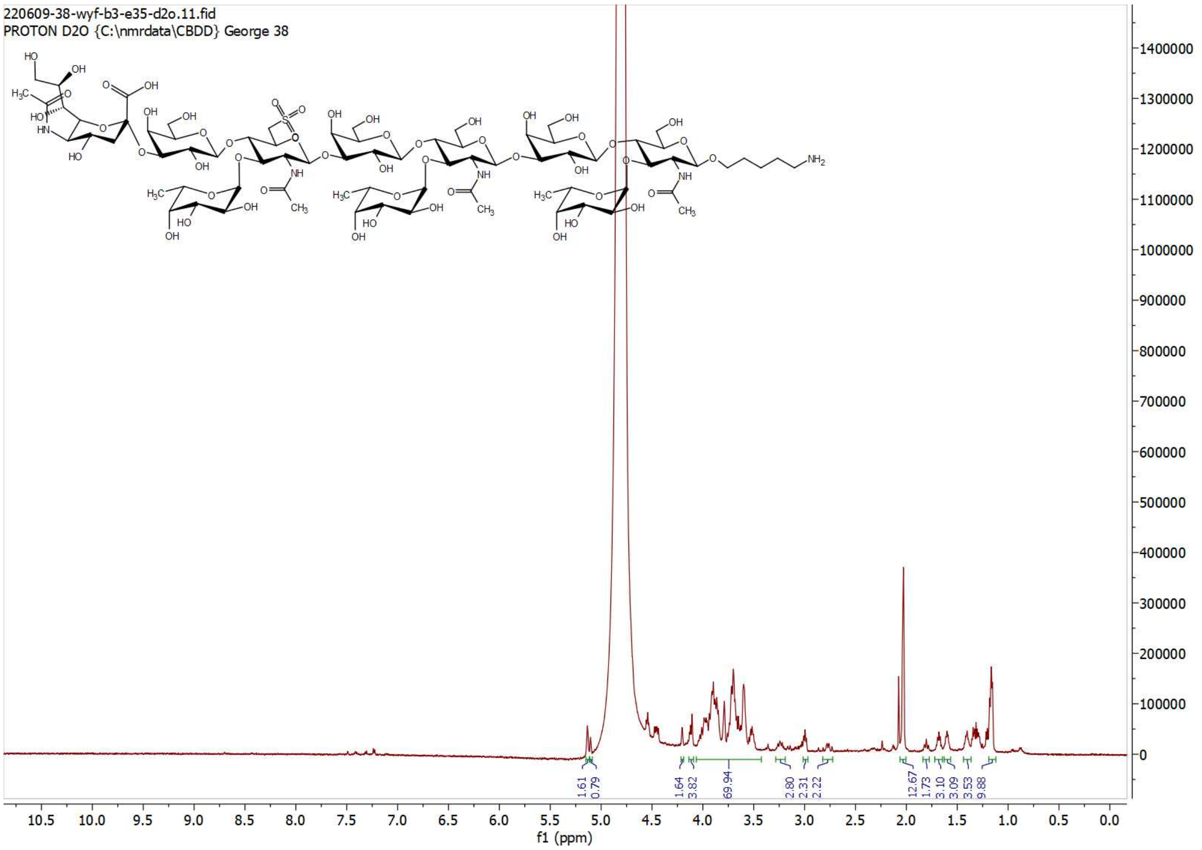

^{1}H NMR of 23; 600 MHz; D_2O

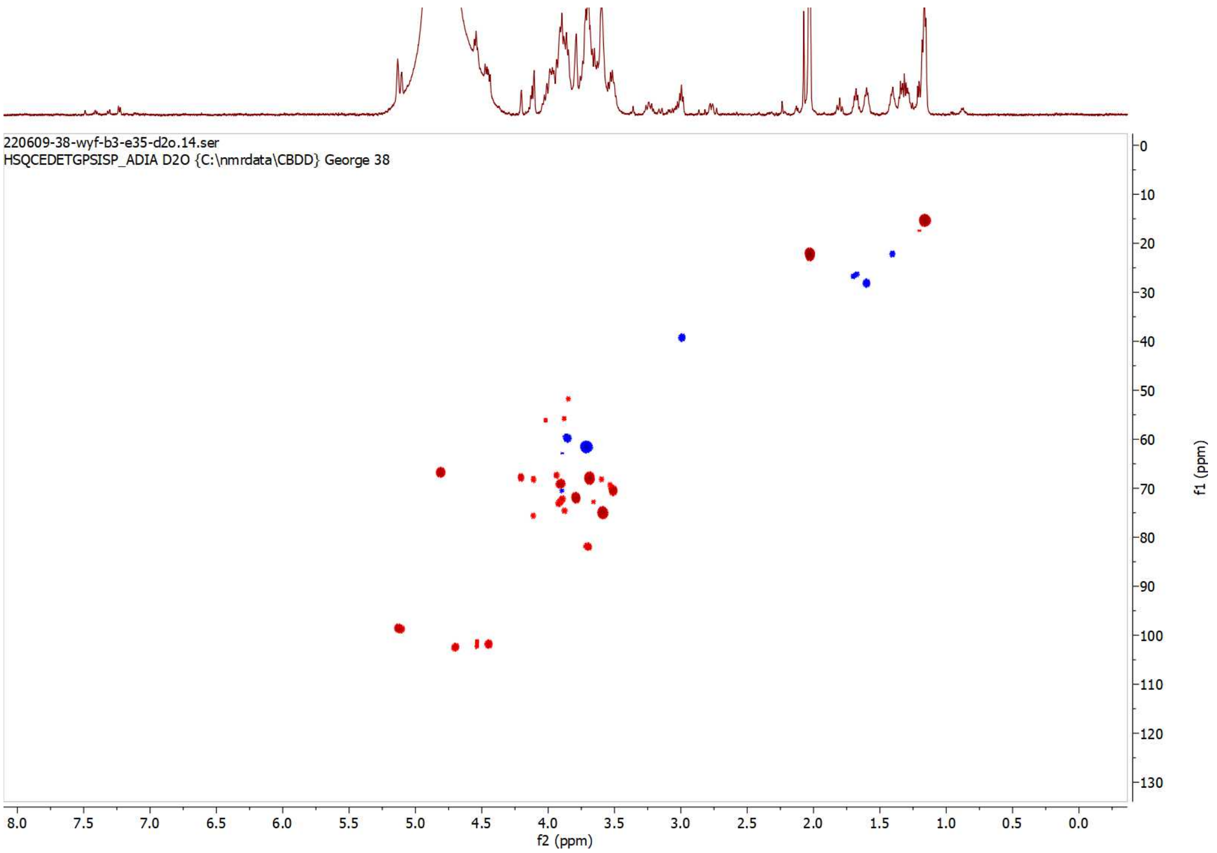

HSQC of 23; 600 MHz/150 MHz, D_2O

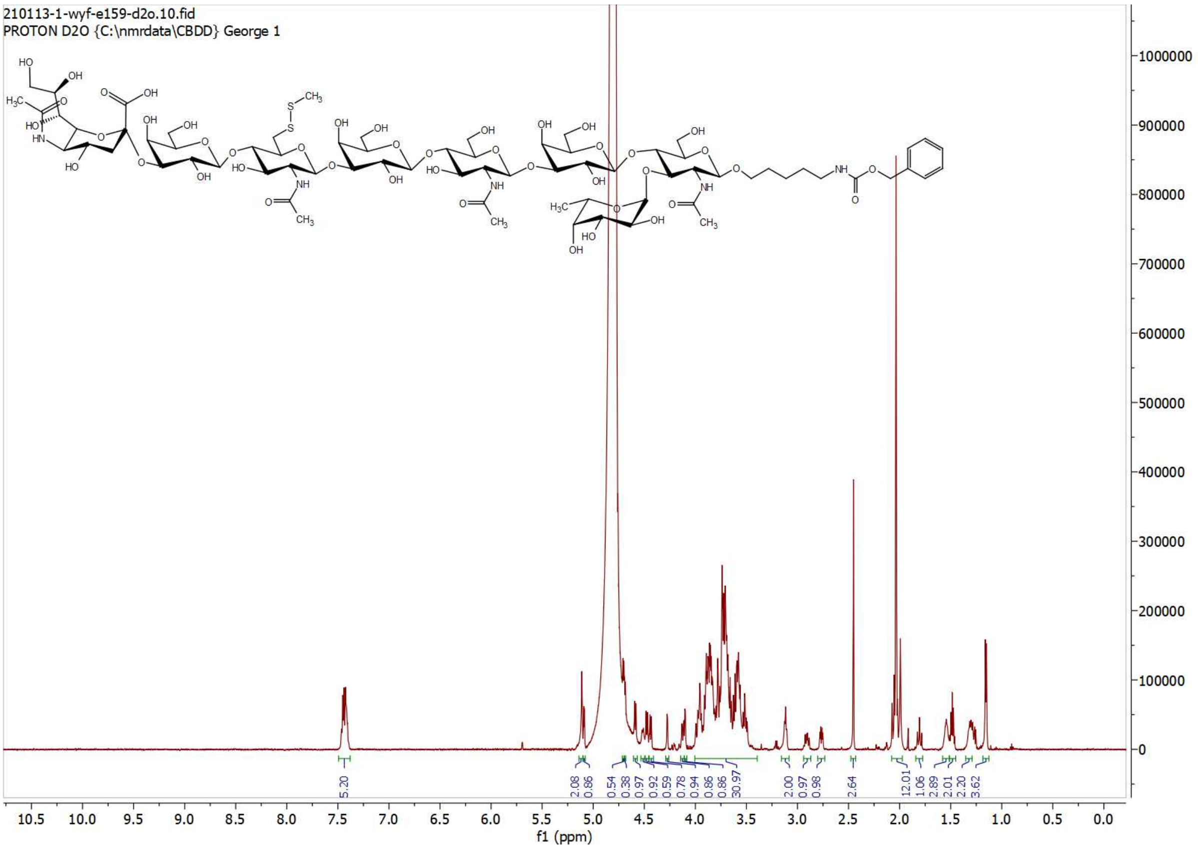

^{1}H NMR of 24; 600 MHz; D_2O

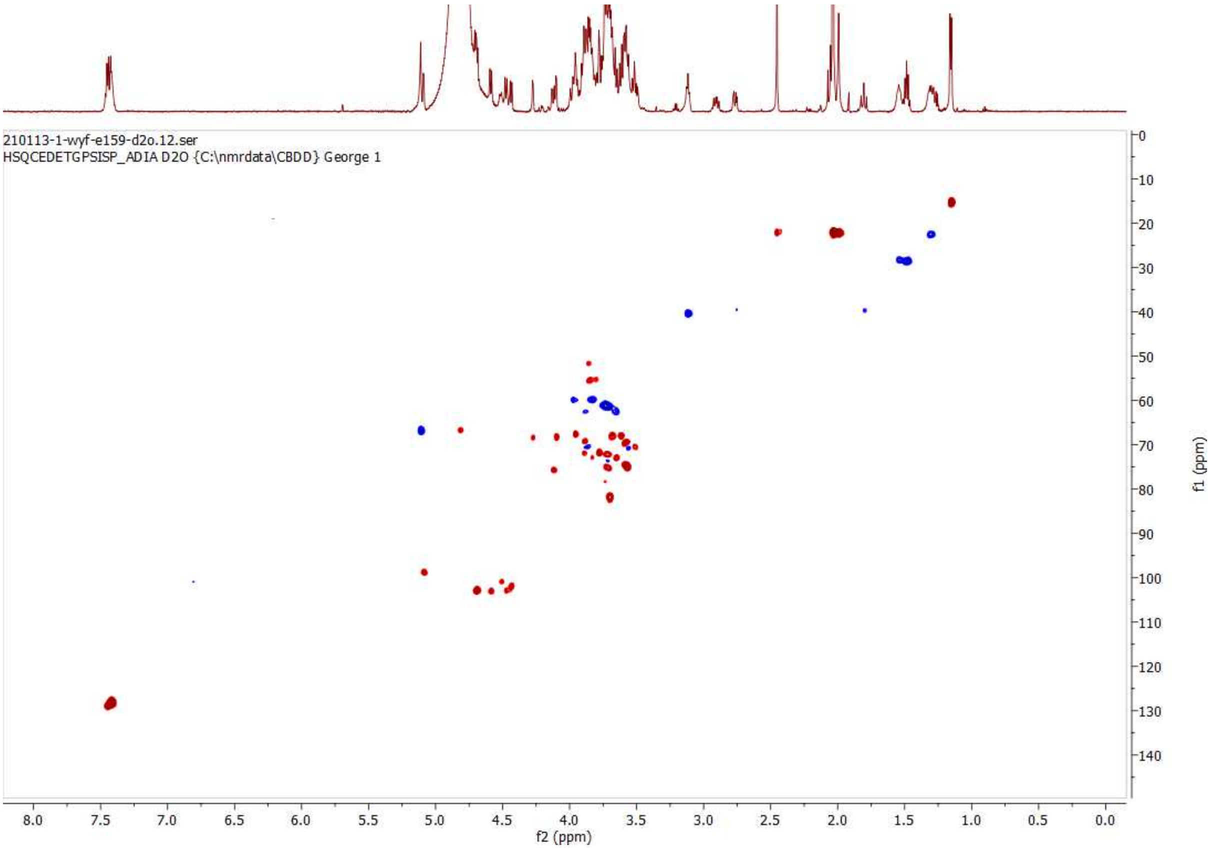

HSQC of 24; 600 MHz/150 MHz, D_2O

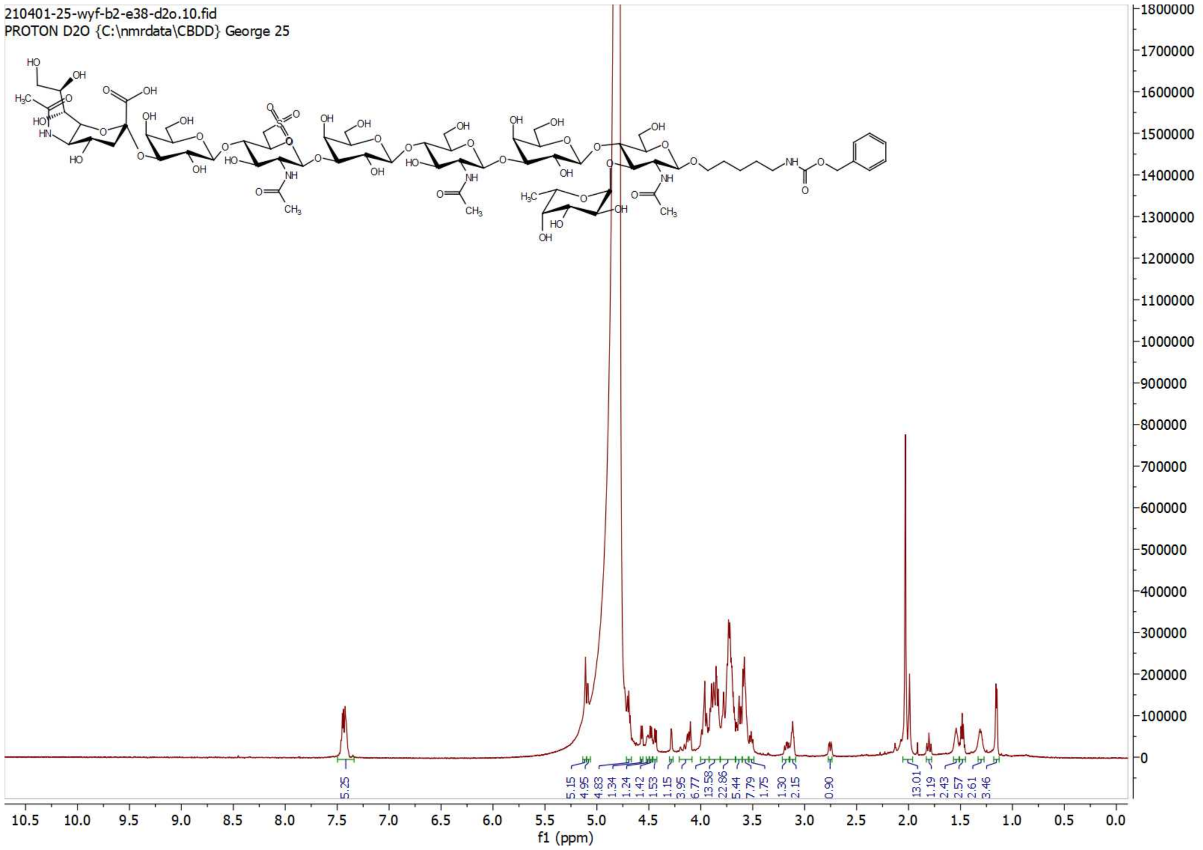

^{1}H NMR of 25; 600 MHz; D_2O

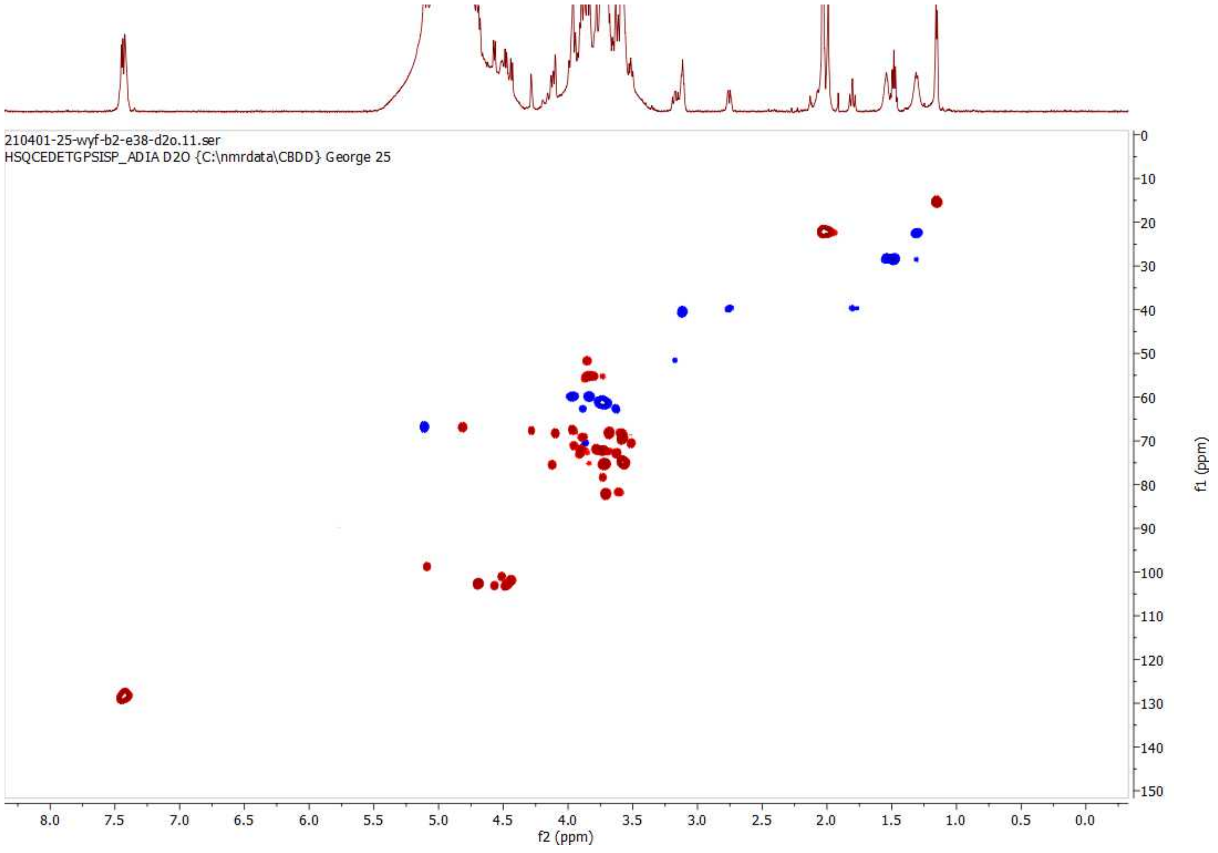

HSQC of 25; 600 MHz/150 MHz, D_2O

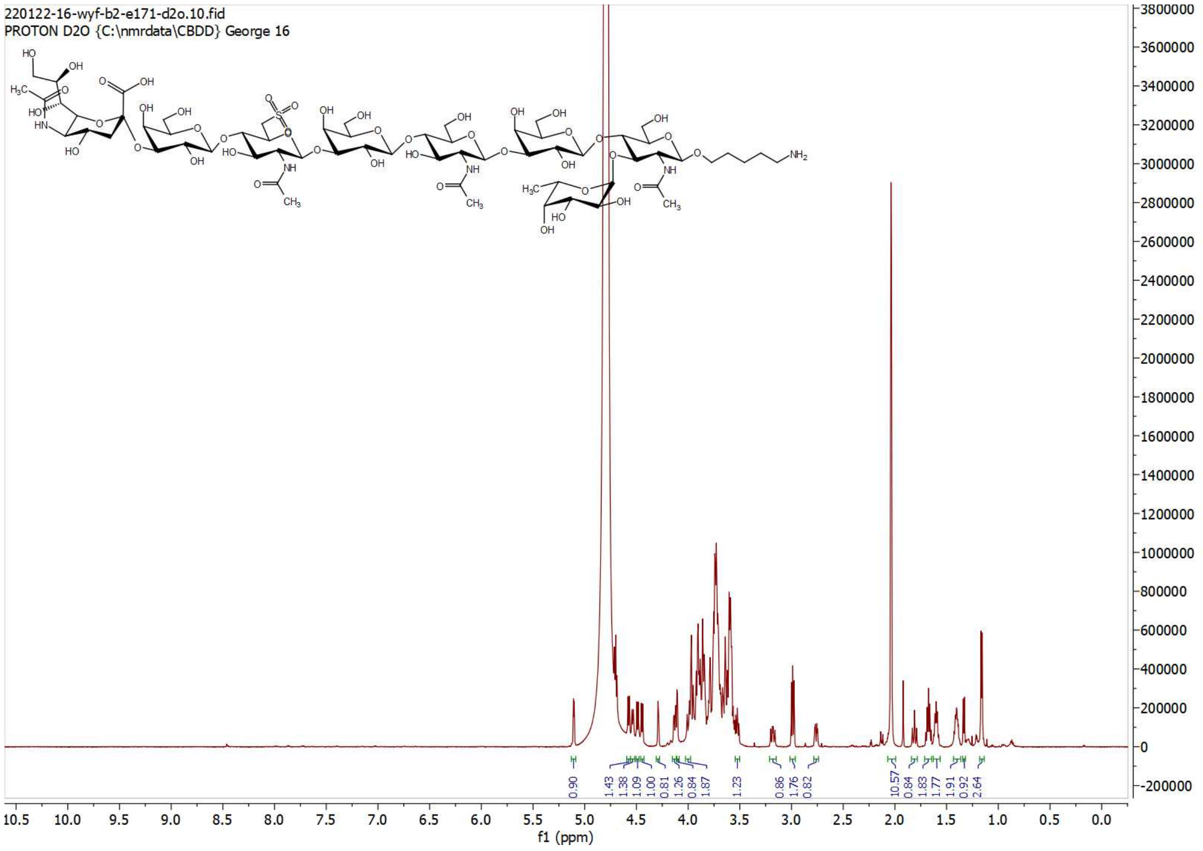

^{1}H NMR of 26; 600 MHz; D$_2$O

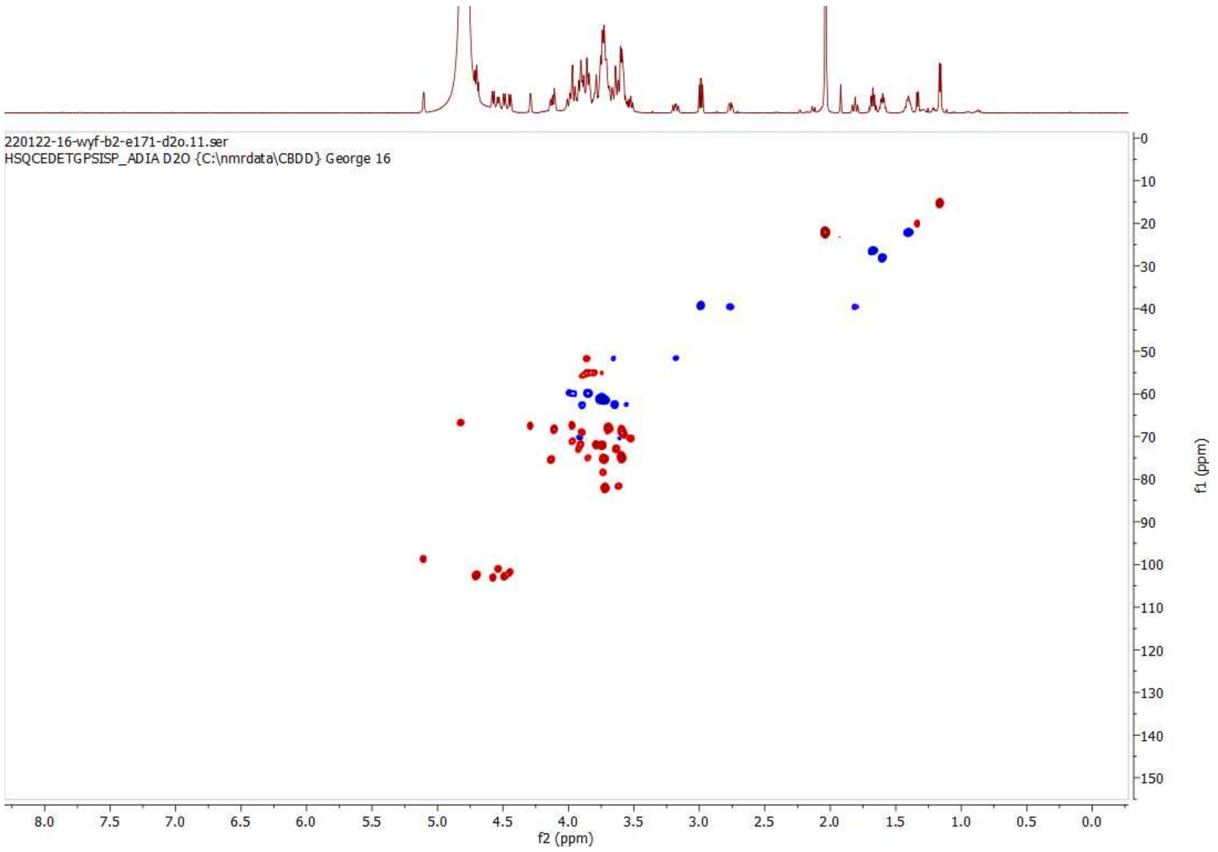

HSQC of 26; 600 MHz/150 MHz, D$_2$O
